# Criterion-Related Validity of Field-Based Methods and Equations for Body Composition Estimation in Adults: A Systematic Review

**DOI:** 10.1007/s13679-022-00488-8

**Published:** 2022-11-11

**Authors:** Nuria Marin-Jimenez, Carolina Cruz-Leon, David Sanchez-Oliva, José Jimenez-Iglesias, Israel Caraballo, Carmen Padilla-Moledo, Cristina Cadenas-Sanchez, Magdalena Cuenca-Garcia, José Castro-Piñero

**Affiliations:** 1 GALENO Research Group, Department of Physical Education, Faculty of Education Sciences, University of Cadiz, Puerto Real, Cadiz, Spain; 2 Instituto de Investigación e Innovación Biomédica de Cádiz (INiBICA), Cadiz, Spain; 3 Sports Science Faculty, University of Extremadura, 10071 Caceres, Spain; 4 PROFITH “PROmoting FITness and Health Through Physical Activity” Research Group, Sport and Health University Research Institute (iMUDS), Department of Physical Education and Sports, Faculty of Sport Sciences, University of Granada, Granada, Spain

**Keywords:** Body composition, Validation studies, Generalized equations, Obesity

## Abstract

***Purpose of Review*:**

Overweight and obesity are associated to health prognosis. Therefore, body composition assessment is an important health outcome, especially in adult population. We analyzed the criterion-related validity of existing field-based methods and equations for body composition estimation in adults aged 19–64 years.

***Recent Findings*:**

One hundred studies met inclusion criteria. The field-based methods, waist circumference (WC), body adiposity index (BAI), and body mass index (BMI) are valid to indicate body adiposity. Likewise, several equations, including the classical Durnin/Womersley equation, Jackson/Pollock equation (males), and Jackson, Pollock, and Ward equation (females), are valid to estimate total body fat mass or body fat percentage.

***Summary*:**

Anthropometric field methods can provide a simple, quick, and easy informative indicators of adiposity in adults. Classical equations, such as Durnin/Womersley equation, Jackson/Pollock equation, and Jackson, Pollock, and Ward equation, are still valid to estimate total body fat mass or body fat percentage in adult population. When choosing estimation equations, specific population characteristics, such as age, weight status, or race ethnicity, should be taken into account. (Trial Registration: Registered on PROSPERO (CRD42020194272)).

**Graphical abstract:**

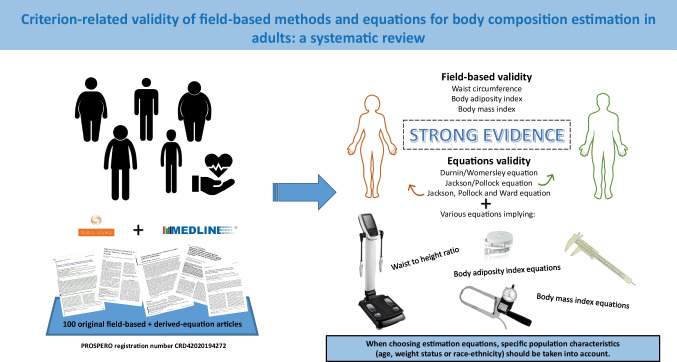

**Supplementary Information:**

The online version contains supplementary material available at 10.1007/s13679-022-00488-8.

## Introduction

Obesity is a pandemic that affects people from childhood to adulthood, being an important risk factor for cardiovascular disease, hypertension, diabetes mellitus, cancer, and premature death [1], in addition to entailing elevated healthcare costs [2] in aging. According to the World Health Organization, in 2016, 39% of adults were affected by overweight and 13% by obesity [3], and obesity will increase up to 20% by 2030 [1]. Therefore, body composition assessment is an important health outcome. Especially in adult population, maintaining adequate levels of body composition, such as reduced body fat or increased muscle and bone mass, is associated with a greater health prognosis [4].

Although in vivo body composition may be measured by “gold standard” such as air displacement plethysmography (ADP), dual-energy x-ray absorptiometry (DXA), deuterium oxide (D_2_O), magnetic resonance imaging (MRI), or underwater weighing (UWW), as a criterion method [5], these devices are limited to certain environments (such as clinical and laboratory setting), expensive, require specialized technicians, and take much time to be used. Therefore, it is necessary to provide the most valid techniques, as well as affordable devices and ease to use, for body fat assessment/distribution. Criterion-related validity refers to the extent to which a field-based test correlates with the criterion measure (i.e., the gold standard) [6]. In this sense, simple and accessible anthropometry assessments, also known as field-based methods (i.e., weight and height, body circumferences, or skinfold (SKF) thickness). are frequently used to determine body fat distribution not only in epidemiological research [5] but also in health-related environments, such as sport or nutrition fields (see Supplementary Fig. S1).

Likewise, body mass index (BMI) (weight/height squared) is also widely used as it is a determinant of unfavorable health consequences [7, 8]. The advantage of BMI is in its ease of calculation, requiring only the metrics of weight and height. However, BMI cannot distinguish between fat and lean mass, and reliance on measurements of BMI alone is still under discussion [7, 9]. For instance, athletes may have BMI values similar to those of an individual with obesity [10]. Another limitation is that different cutoffs are established for Caucasian/White, African American/Black, Hispanic, and the Asian and South Asian populations [10] since it underestimates the obesity risk in the latter. Finally, BMI seems to be also sex-dependent, showing a higher body fat percentage (%BF) in females than in males with the same BMI [11, 12]. Therefore, new indexes, such as body adiposity index (BAI) (hip circumference / (height)^1.5^ – 18) [13], have emerged to address these limitations.

Similarly, body circumferences, such as waist circumference (WC), hip circumference (HC), waist-to-hip circumference ratio (WHR) (waist circumference/hip circumference), and waist-to-height ratio (WHtR) (waist circumference/height), are simple methods to indicate abdominal adiposity and are widely associated with adverse health risks, such as cardiovascular disease or mortality in adults [9]. Finally, SKF have the advantage of providing localized information about the thickness of subcutaneous fat tissue. Therefore, different regression equations, based on SKF, were developed to calculate body fat mass and/or %BF [5]. Moreover, a great amount of estimation equations (based on others anthropometry measurements) have been also proposed. Since the 1970s, Durnin and Womersley generalized equations [14], Jackson and Pollock [15] (in male adults), and Jackson, Pollock, and Ward [16] (in female adults) equations have been widely used to estimate %BF, for their simplicity of calculation. However, a universal equation for predicting body composition cannot simply explain the variation of body types and shapes around the world (i.e., different race ethnicities and weight statuses). Additionally, it is still under discussion which anthropometric measurements or estimation equations are the most valid to calculate body fat mass and/or BF% [5].

Assessing body composition may be difficult in large epidemiological studies with limited access to advanced assessment methods. Therefore, it is of particular interest to practitioners to balance the practicality and feasibility of performing body composition measurements and equations in a non-laboratory setting. Our systematic review aimed to identify studies evaluating the criterion-related validity of existing field-based methods and equations for body composition estimation used in adults aged 19–64 years.

## Methods

The present review was registered in PROSPERO (registration number CRD42020194272) and the methodology applied followed the guidelines drawn in the Preferred Reporting Items for Systematic Reviews and Meta-Analysis (PRISMA) statement [17].

### Data Sources

Data were obtained from direct online access to and searches of the following biomedical bibliographic databases: MEDLINE (via PubMed) and Web of Science (all databases).

### Information Search

The search terms were defined as follows: Medical Subject Headings (MeSH) and the controlled medical vocabulary thesaurus developed by the US National Library of Medicine. The search terms used in the search strategy were related to the following topics: (i) *participants*, adult population (aged 19–64 years); (ii) *validity terms*, criterion-related validity, validity, validation, estimation, prediction, and cross-validation; and (iii) *body composition assessment*, WC, neck circumference (NC), HC, SKF, BAI, bioelectrical impedance analysis (BIA), BMI, fat mass index, WHR, and WHtR. The equation for the final search was developed for use in the MEDLINE database, via PubMed, using Boolean connectors. The same strategy was adapted to Web of Science (the final equations are displayed in Supplementary Material S1). The search was carried out from the first available date, in accordance with the characteristics of each database, until November 2021, and was completed by examining the bibliographic references of the selected articles.

### Eligibility Criteria

The inclusion criteria for this systematic review were (i) age: adults (19–64 years old). During the review, we faced the problem that some studies sampled adults and older adults or adults and adolescents together. In these cases, we observed whether these studies performed stratified analyses by age groups, isolating the adult population from the rest; if so, the study was included and information concerning the adult population was reported. In contrast, when the authors analyzed the whole sample together, we only included the study if the age of the sample was predominantly within our study age range; (ii) participants, the study population was based on a general healthy population, who did not present any injury, physical and/or mental disabilities, irrespective of BMI, diabetes, or other cardiovascular risks (i.e., hypertension, hypercholesterolemia, lipid profiles, glucose levels, insulin sensitivity); (iii) study design, original studies; (iv) language, articles only published in English or Spanish and; and (v) topic, studies examining the criterion-related validity of field-based body composition methods. Studies comparing field-based body composition methods with a non-gold standard criterion were excluded. Likewise, studies that analyzed the criterion-related validity of tests designed for exclusive use in sports or clinical settings were not included.

### Study Selection

**Fig. 1:**
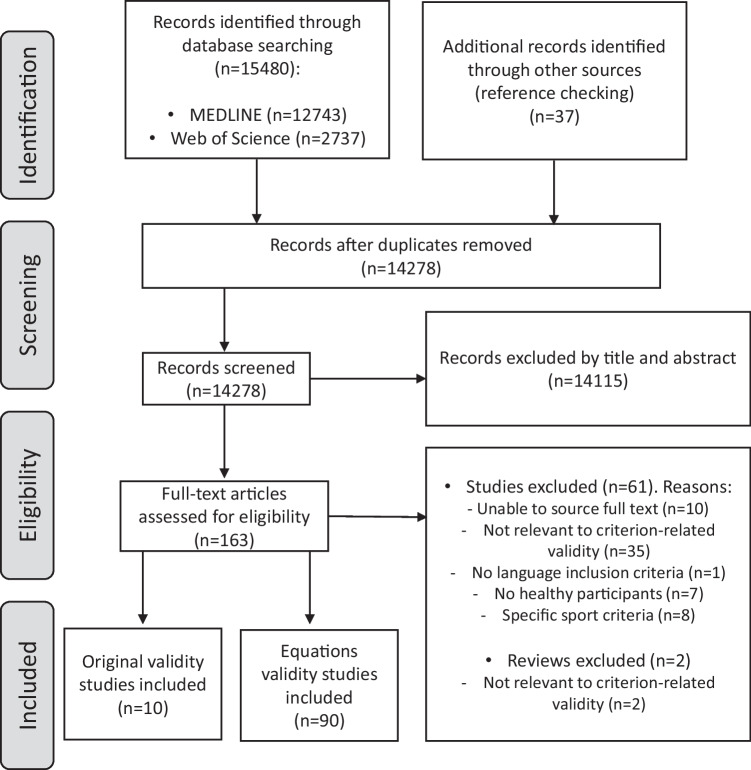
Flow chart of retrieved and selected articles

Two researchers (NMJ and CCL) independently assessed titles and abstracts of the articles retrieved by the search strategy for eligibility, after checking for duplicates. Then, the full texts of the selected articles were acquired, and the same two researchers independently screened them to determine whether to include the article based on the inclusion criteria. When no consensus was reached between both researchers, a third researcher (JCP) made the final decision about inclusion. Reasons for exclusion of identified articles were recorded (see Supplementary Material S2 and Fig. 1).

### Data Extraction

Two researchers (NMJ and CCL) independently extracted the following information from each eligible original study according to standardized form: (i) author’s name; (ii) participants’ characteristics (i.e., sex, number, BMI status, and race ethnicity); (iii) age of participants; (iv) filed-based methods or estimation equations; (v) criterion measure (gold standard); (vi) statistical methods; (vii) results; and (viii) conclusions. Disagreements in the extracted data were discussed between researchers until a consensus was reached. Due to the heterogeneity of statistical methods within the original studies selected, the high number of tests included, and the limited number of studies per test, a meta-analysis was not conducted.

When the main statistical analysis was linear regression, the strength of the validity of each selected study was classified as follows: 0.00–0.25, very low; 0.26–0.49, low; 0.50–0.69, moderate; 0.70–0.89, high; and 0.90–1.00, very high [18] (see Supplementary Tables S4 and S5).

### Criteria for Risk of Bias Assessment

An assessment of the risk of bias in selected original studies and systematic reviews was made for each eligible study by two researchers (NMJ and CCL), independently. Discrepancies were solved in a consensus meeting. The inter-rater agreement for the selected studies was 96% (Kappa coefficient = 0.96) and 100% agreement after consensus meeting.

The assessing risk of bias criteria in original studies was determined according to quality assessment list employed by Castro-Piñero et al. [19], which include the following three criteria: (i) number of participants; (ii) description of the study population; and (iii) statistical analysis (see Supplementary Table S1). Each criterion was rated from 0 to 2, 2 being the best score. For all studies, a total score was calculated by counting up the number of positive items (a total score between 0 and 6). Studies were categorized as very low quality (0–2), low quality (3–4), and high quality (5–6) (see Supplementary Tables S2 and S3). Only high-quality studies were analyzed to construct levels of evidence.

### Levels of Evidence

Three levels of evidence [19] were constructed: (1) *strong evidence*, consistent findings in three or more high-quality studies; (2) *moderate evidence*, consistent findings in two high-quality studies; and (3) *limited evidence*, consistent findings in multiple low-quality studies, inconsistent results found in multiple high-quality studies, or results based on one single study. The degree of the criterion-related validity of the field-based methods and estimation equations will be discussed for those tests on which we have found strong or moderate evidence that the test is (or not) valid.

## Results

In total, 15,517 references were found, 12,743 in MEDLINE and 2737 in the Web of Science. Additionally, 37 records were identified through other sources (i.e., reference checking). After removing duplicates and applying inclusion and exclusion criteria, *100* studies were selected. Of them, *10* as field-based studies and *90* as estimation equations studies (see Fig. 1).

### Risk of Bias Within Studies

Of the 100 original studies included in the present systematic review, one field-based study [20] and 12 estimation equations studies [21–32] were classified as low quality (a total score of 3 and 4). A total of 87 original studies were classified as high-quality (a total score higher than 4) (see Supplementary Tables S2 and S3).

### Characteristics of Included Studies

The studies included a highly variable number of participants, from 30 to 18,198. A total of 59,161 female and 43,931 male adults were included (average age of 39.9 years). BMI status ranges from normal weight to obese class 3. Non-standard groupings were used to classify participants, such as Caucasian/White [7, 11, 12, 20, 25, 28, 30, 33–67] (*n* = 43), African American/Black [7, 11, 13, 34, 35, 38–42, 44, 46, 53, 57, 58, 60, 61, 66–68] (*n* = 20), Asian [38, 40, 44, 50, 58, 62, 69–77] (*n* = 15), Mexican American/Hispanic [7, 13, 34, 39–42, 44, 46, 53, 61, 78, 79] (*n* = 13), African Indian [80–82] (*n* = 3), Brazilian [83, 84] (*n* = 2), Colombian [85, 86] (*n* = 2), Anglo-Celtic Australian [87] (*n* = 1), Chinese Australian [88] (*n* = 1), Australian [89] (*n* = 1), European American[90] (*n* = 1), Syrian [91] (*n* = 1), Guatemalan [92] (*n* = 1), Polynesian [65] (*n* = 1), Chilean [93] (*n* = 1), Canadian [94] (*n* = 1), Portuguese [95] (*n* = 1), Tasmanian [96] (*n* = 1), or Swedish [97] (*n* = 1). Whenever possible, these race ethnicities have been grouped into four large groups [98] (i.e., Caucasian/White, African American/Black, Hispanic, Asian) to discuss findings. However, in other cases. race ethnicity or native country has not been specifically reported [14–16, 21–24, 26, 27, 29, 31, 32, 99–110] (*n* = 24).

One field-based study [111] and 10 estimation equations studies [7, 42, 49, 52, 54, 86, 93, 99, 101, 112] were published in the last 5 years (see Supplementary Tables S4 and S5).

### Criterion-Related Validity of Field-Based Body Composition Estimation in Adults

The results of validity of field-based body composition estimation in adults can be seen in the Supplementary Material S3 and Supplementary Table S4. According of these results, we established the levels of evidence (see Table 1).

**Table 1:**
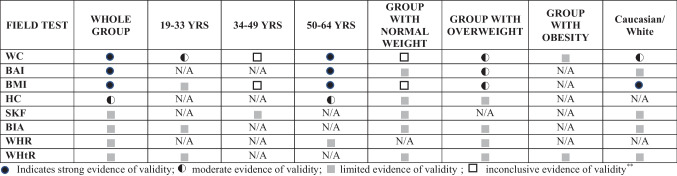
Levels of evidence of field-based body composition estimation studies in adults

*HC* hip circumference, *WC* waist circumference, *SKF* skinfolds, *BAI* body adiposity index, *BIA* bioelectrical impedance analysis, *BMI* body mass index, *WHR* waist-to-hip ratio, *WHtR* waist-to-height ratio, *N/A* not applicable

*Limited evidence of validity derived from one single study; **Inconclusive evidence of validity derived from inconsistent results

Regarding the whole group, (a) strong evidence indicated that WC, BAI, and BMI are valid to estimate body adiposity; (b) moderate evidence showed that HC is valid to estimate body adiposity; and (c) limited evidence was found for SKF, BIA, WHR, and WHtR.

### Age Groups

Regarding age-specific groups, the mean age was 44.6 years old. To compare the results, three tertiles were constructed (*first group*, 19–33; *second group*, 34–49; and *third group*, 50–64 years old). According of these results, we established the levels of evidence (see Table 1).

In *the 19–33-year-old group*, (a) moderate evidence showed that WC is valid to estimate body adiposity; (b) there was limited evidence about the validity of WHtR, BIA, and BMI. *In the 34–49-year-old group*, (a) inconclusive evidence was found for WC and BMI, and there was limited evidence about the validity of SKF. *In the 50–64-year-old group*, (a) strong evidence indicated that WC, BMI, and BAI are valid to estimate total body adiposity; (b) moderate evidence showed that HC is valid to estimate total body adiposity; and (c) there was limited evidence about the validity of WHR.

### Weight Status Groups

Regarding BMI status, we observed a mean BMI of 25.7 kg/m^2^. Participants were classified by BMI status, according to the World Health Organization [10], in three groups: normal weight, ≥ 18.5–24.9 kg/m^2^; overweight, 25–29.9 kg/m^2^; and obesity ≥ 30 kg/m^2^. According of these results, we established the levels of evidence (see Table 1).

For *group with normal weight*, (a) inconclusive evidence was found for WC and BMI; (b) there was limited evidence about the validity of HC, SKF, BAI, BIA, and WHtR. For *group with overweight*, (a) moderate evidence showed that WC, BMI, and BAI are valid to estimate body adiposity; (b) there was limited evidence about the validity of HC, BIA, WHR, and WHtR. For *group with obesity*, (a) there was limited evidence about the validity of WC and WHtR.

### Race Ethnicity Groups

Race ethnicity was classified, in an attempt to discuss the findings of the present systematic review, following the latest report for medical and science journals [98]. According of these results, we established the levels of evidence (see Table 1).

In *Caucasian/White* adults, (a) strong evidence indicated that BMI is valid to estimate body adiposity; (b) moderate evidence showed that WC is valid to estimate body adiposity; and (c) there was limited evidence about the validity of SKF, BAI, BIA, and WHtR.

Due to heterogeneity found in other races ethnicities, no further level of evidence could be established.

### Criterion-Related Validity of Estimation Equations for Body Composition in Adults

The results of validity of estimation equations for body composition in adults can been seen in the Supplementary Material S and Supplementary Table S5. According to these results, we constructed the levels of evidence (see Table 2).

**Table 2:**
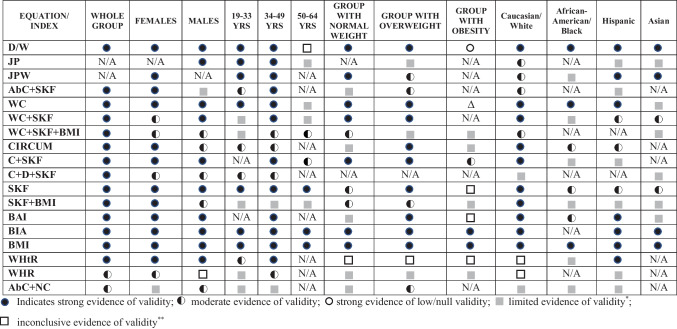
Levels of evidence of estimation equations studies in adults

*D/W* Durnin/Womersley equation, *JP* Jackson/Pollock equation, *JPW* Jackson, Pollock, and Ward equation, *AbC* + *NC* equations combining abdominal circumference + neck circumference, *AbC* + *SKF* equations combining abdominal circumference + skinfolds, *WC* equations based on waist circumferences, *WC* + *SKF* equations combining waist circumference + skinfolds, *WC* + *SKF* + *BMI* equations combining waist circumference + skinfolds + body mass index, *Circum* equations combining various circumferences, *C* + *SKF* equations combining circumferences + skinfolds, *C* + *D* + *SKF* equations combining circumferences + diameters + skinfolds, *SKF* equations implying sum of skinfolds, *SKF* + *BMI* equations combining skinfolds + body mass index, *BAI* body adiposity index equations, *BIA* bioelectrical impedance analysis equations, *BMI* body mass index equations, *WHR* waist-to-hip ratio equations, *WHtR* waist-to-height ratio equations, *N/A* not applicable

*Limited evidence of validity derived from one single study; **Inconclusive evidence of validity derived from inconsistent results

Regarding the whole group, (a) strong evidence indicated that Durnin/Womersley equation, BMI equations, BIA equations, equations implying sum of SKF, equations combining circumferences + SKF, equations based on WC, equations combining WC + SKF, BAI equations, equations combining various circumferences, WHtR equations, equations combining AbC + SKF, equations combining WC + SKF + BMI, equations combining circumferences + diameters + SKF, and equations combining SKF + BMI are valid to estimate total body fat mass or %BF; (b) moderate evidence showed that equations combining AbC + NC, and WHR are valid to estimate total body fat mass or %BF.

### Sex Groups

The levels of evidence in *female adults* were (a) strong evidence indicated that Durnin/Womersley equation; Jackson, Pollock, and Ward equation; BMI equations; BIA equations; equations combining circumferences + SKF; equations implying sum of SKF; BAI equations; equations based on WC; equations combining various circumferences; equations combining AbC + SKF; and equations combining SKF + BMI, and WHtR equations are valid to estimate total body fat mass or %BF; (b) moderate evidence showed that equations combining WC + SKF, equations combining WC + SKF + BMI, equations combining circumferences + diameters + SKF, and WHR equations are valid to estimate total body fat mass or %BF; and (c) there was limited evidence about the validity of equations combining AbC + NC. *In male adults*, (a) strong evidence indicated that Durnin/Womersley equation, Jackson/Pollock equation, BMI equations, equations implying sum of SKF, BIA equations, equations based on WC, equations combining circumferences + SKF, BAI equations, equations combining WC + SKF, and WHtR equations are valid to estimate total body fat mass or %BF; (b) moderate evidence showed that equations combining AbC + NC, equations combining WC + SKF + BMI, equations combining various circumferences, equations combining circumferences + diameters + SKF, and equations combining SKF + BMI are valid to estimate total body fat mass or %BF; (c) there was limited evidence about the validity of equations combining AbC + SKF (derived from a single study); and (d) inconclusive evidence was found for WHR equations (see Table 2).

### Age Groups

The levels of evidence in *the 19–33-year-old group* were (a) strong evidence indicated that Durnin/Womersley equation; Jackson/Pollock equation; Jackson, Pollock, and Ward equation; BIA equations; equations implying sum of SKF; BMI equations; and equations based on WC are valid to estimate total body fat mass or %BF; (b) moderate evidence showed that equations combining AbC + SKF, equations combining various circumferences, equations combining circumferences + diameters + SKF, and WHtR equations are valid to estimate total body fat mass or %BF; and (c) there was limited evidence about the validity of equations combining AbC + NC, equations combining WC + SKF, equations combining WC + SKF + BMI, equations combining circumferences + SKF, equations combining SKF + BMI, BAI equations, and WHR equations (derived from a single study). *In the 34–49-year-old group*, (a) strong evidence indicated that Durnin/Womersley equation; Jackson/Pollock equation; Jackson, Pollock, and Ward equation; BMI equations; equations combining circumferences + SKF; equations implying sum of SKF; BIA equations; BAI equations; equations based on WC; equations combining AbC + SKF; equations combining WC + SKF; and WHtR equations are valid to estimate total body fat mass or %BF; (b) moderate evidence showed that equations combining WC + SKF + BMI, equations combining various circumferences, equations combining circumferences + diameters + SKF, and WHR equations are valid to estimate total body fat mass or %BF; and (c) there was limited evidence about the validity of equations combining AbC + NC and equations combining SKF + BMI (derived from a single study). In the 50–64-year-old group, (a) strong evidence indicated that BMI equations, equations implying sum of SKF, and BIA equations are valid to estimate total body fat mass or %BF; (b) moderate evidence showed that equations combining WC + SKF + BMI and equations combining circumferences + SKF are valid to estimate total body fat mass or %BF; (c) there was limited evidence about the validity of Jackson/Pollock equation, equations combining WC + SKF, equations combining SKF + BMI, and equations based on WC (derived from a single study); and (d) inconclusive evidence was found for Durnin/Womersley equation (see Table 2).

### Weight Status Groups

The levels of evidence in the *group with normal weight* were (a) strong evidence indicated that Durnin/Womersley equation; Jackson, Pollock, and Ward equation; BMI equations; BIA equations; equations based on WC; equations combining WC + SKF; and equations combining circumferences + SKF are valid to estimate total body fat mass or %BF; (b) moderate evidence showed that equations combining WC + SKF + BMI, equations implying sum of SKF, and equations combining SKF + BMI are valid to estimate total body fat mass or %BF; (c) there was limited evidence about the validity of equations combining AbC + NC, equations combining AbC + SKF, equations combining various circumferences, BAI equations, and WHR equations (derived from a single study); and (d) inconclusive evidence was found for WHtR equations. *Group with overweight*, (a) strong evidence indicated that Durnin/Womersley equation, BMI equations, BIA equations, equations combining circumferences + SKF, equations based on WC, equations combining WC + SKF, equations combining various circumferences, BAI equations, and equations implying sum of SKF are valid to estimate total body fat mass or %BF; (b) moderate evidence showed that Jackson, Pollock, and Ward equation, equations combining AbC + NC, equations combining AbC + SKF, and equations combining SKF + BMI are valid to estimate total body fat mass or %BF; (c) there was limited evidence about the validity of Jackson/Pollock equation, equations combining WC + SKF + BMI, and WHR equations (derived from a single study); and (d) inconclusive evidence was found for WHtR equations. *Group with obesity*, (a) strong evidence indicated that Durnin/Womersley equation is not valid to estimate total body fat mass or %BF; BMI equations and BIA equations are valid to estimate total body fat mass or %BF; (b) moderate evidence showed that equations combining circumferences + SKF are valid to estimate total body fat mass or %BF; (c) there was limited evidence about the validity of equations combining SKF + BMI, WHR equations, equations combining WC + SKF + BMI, and equations combining various circumferences (derived from a single study); and (d) *inconclusive evidence* was found for BAI equations, equations based on WC, equations implying sum of SKF, and WHtR equations (see Table 2).

### Race Ethnicity Groups

The levels of evidence in *Caucasian/White adults* were (a) strong evidence indicated that Durnin/Womersley equation, BMI equations, equations combining circumferences + SKF, equations implying sum of SKF, equations based on WC, equations combining various circumferences, equations combining WC + SKF, equations combining SKF + BMI, BIA equations, and BAI equations are valid to estimate total body fat mass or %BF; (b) moderate evidence showed that Jackson/Pollock equation, Jackson, Pollock, and Ward equation; equations combining AbC + SKF; and equations combining WC + SKF + BMI are valid to estimate total body fat mass or %BF; (c) there was limited evidence about the validity of equations combining AbC + NC and equations combining circumferences + diameters + SKF (derived from a single study); and (d) inconclusive evidence was found for WHR equations and WHtR equations. *In African American/Black adults*, (a) strong evidence indicated that Durnin/Womersley equation, BMI equations, and equations based on WC are valid to estimate total body fat mass or %BF; (b) moderate evidence showed that equations combining various circumferences, equations implying sum of SKF, and BAI equations are valid to estimate total body fat mass or %BF; and (c) there was limited evidence about the validity of Jackson, Pollock, and Ward equation, equations combining AbC + NC, equations combining WC + SKF, equations combining circumferences + SKF, equations combining circumferences + diameters + SKF, equations combining SKF + BMI, and WHtR equations (derived from a single study). *In Hispanic adults*, (a) strong evidence indicated that Durnin/Womersley equation; Jackson, Pollock, and Ward; equations based on WC; BIA equations; BAI equations; BMI equations; and WHtR equations are valid to estimate total body fat mass or %BF; (b) moderate evidence showed that equations combining WC + SKF, equations combining various circumferences; and equations implying sum of SKF; and (c) there was limited evidence about the validity of Jackson/Pollock equation, equations combining AbC + NC, equations combining AbC + SKF, equations combining circumferences + SKF, equations combining SKF + BMI, and WHR equations (derived from a single study). *In Asian adults*, (a) strong evidence indicated that Durnin/Womersley equation; Jackson, Pollock, and Ward equation; BIA equations; and BMI equations are valid to estimate total body fat mass or %BF; (b) moderate evidence showed that equations combining WC + SKF and equations implying sum of SKF are valid to estimate total body fat mass or %BF; (c) there was limited evidence about the validity of Jackson/Pollock equation, equations based on WC, equations combining WC + SKF + BMI, equations combining circumferences + SKF, equations combining SKF + BMI, and BAI equations (derived from a single study). Due to heterogeneity found in other race ethnicities, no further level of evidence could be established (see Table 2).

## Discussion

As obesity data is rising, practitioners and researchers are more frequently interested in evaluating body composition to better manage it, improve treatment decisions, and optimize health outcomes. Therefore, we have systematically examined the validity of different field-based methods and equations for body composition estimation in the adult population that may provide accurate results, minimizing prediction error, compared with “gold standard” methods.

The main findings of this systematic review were (a) strong evidence indicated that WC, BAI, and BMI are valid to estimate body adiposity; (b) in estimation equation studies, strong evidence indicated that Durnin/Womersley equation, Jackson/Pollock equation (males), and Jackson, Pollock, and Ward equation (females), BMI equations, BIA equations, equations implying sum of SKF, equations combining circumferences + SKF, equations based on WC, equations combining WC + SKF, BAI equations, equations combining various circumferences, WHtR equations, equations combining abdominal circumference + SKF, equations combining WC + SKF + BMI, equations combining circumferences + diameters + SKF, and equations combining SKF + BMI are valid to estimate total body fat mass or %BF.

The findings of this systematic analysis are largely heterogeneous. The methods to estimate body composition, both field-based methods and criterion methods, and the estimation equations used in the studies are also variable, further contributing to the heterogeneity of the collected data. Thus, it is still challenging to establish valid methods for the field assessment of human body composition due to specific differences in age, sex, race ethnicity, health status, weight status, etc. Despite the difficulty in drawing meaningful conclusions from this data set, the most relevant findings (i.e., those with moderate and strong evidence of validity), including main advantages and limitations, are discussed below (see Supplementary Table S6).

### Criterion-Related Validity of Field-Based Body Composition Estimation in Adults

The measurement of field-based body composition is very easy and cost-effective and, therefore, frequently used in epidemiologic studies. Numerous obesity indexes have been applied to characterize obesity, being BMI by far the most common approach in clinical practice. The main advantage of using BMI as an overall index of adiposity is its predictive power for clinical outcomes, such as cardiovascular mortality [113]. However, despite its worthiness, BMI can vary between individuals, making it an insufficient method to measure health-related body composition, as increased muscle mass and weight may falsely increase its BMI, as in athlete population. Moreover, it is also known its limitations regarding age, sex, and race ethnicity [113]. Although the same BMI classification is used for both female and male adults, at the same BMI, females tend to have more body fat than males and to have abdominal obesity (WC ≥ 88 cm in females or ≥ 102 cm in men) [10], especially during the stages of pregnancy or menopause. Regarding age, at the same BMI, older people tend to have more body fat than younger adults [10]. Likewise, different cutoffs are established for White, Hispanic, and Black individuals and the Asian and South Asian populations [10]. For the first group, a BMI > 25.0 kg/m^2^ is defined as overweight, while in the second group, overweight status is pointed out from 23.0 kg/m^2^ [10]. Thus, at the same BMI, White/Hispanic have more body fat than Blacks, and Asians have more body fat than do Whites [10].

Therefore, other indexes have emerged trying to overcome these limitations, such as BAI [13], WC, WHR, or WHtR. The main advantage of these indexes is the ease and speed of measurement (just needing a non-elastic tape) and calculation, being suitable for clinical settings and epidemiological studies. Moreover, all of them imply central adiposity measurement, which provides independent and additive information to BMI to predict morbidity and mortality risk [9]. Likewise, height may influence the fat distribution, being more accurate than BMI (a decrease in height will increase BMI without an increase in fat mass) [96], and short stature is associated with an increased risk of cardiometabolic health complications [9]. Notwithstanding, height is only marginally associated with WC, and WHtR seems less useful as, in adults, the height is generally fixed and the value can only be changed by changes in WC [9]. One disadvantage of BAI is that HC by itself does not seem to be a good estimator of %BF [59, 75]. Therefore, although strong evidence has been found, it is possible that other studies failed to find validity based on this fact. Thereby, some caution is still warranted when using this novel index as a measure of body composition alone. Similarly, and regarding WHR, the relocation of fat to a more central distribution and loss of muscle mass in the gluteal region will increase WC and WHR without increasing fat mass [96]. For these reasons, it seems that the simple measurement of WC can provide relevant clinical information in the management of obesity [114]. Indeed, among the numerous existing circumference measurements, WC is the most used. WC correlates with total body fat and is often used as a measure of central obesity [114]. In addition, WC can be especially useful for stratifying high-risk people classified as overweight or with obesity class 1 and is also a key part of the definition of metabolic syndrome [114]. The main drawback of WC is that the measure may be influenced by the timing of measurement (e.g., preprandial and postprandial period), and it requires the individual to remove clothing to obtain a more accurate measurement, so some people may be uncomfortable with this measurement [115]. Furthermore, population-specific cutoff points have been proposed [116].

Finally, the BIA device, which can be considered as a field method, is also widely used in clinical, research and field settings, due to its relatively low cost and portable. However, BIA presents some bias, due to under or overestimation in body fat [111], especially in measuring individuals with severe obesity [117].

In summary, the field approach to body composition assessment highlights it applicability in non-laboratory settings, with WC being the most commonly, quickest, simplest, and economical method.

### Criterion-Related Validity of Estimation Equations Body Composition Assessment in Adults

In addition to field-based methods, estimation equations help implement information about an individual’s body composition. Consequently, the most accurate estimation is the clinical importance. However, due to typical time and resource constraints, a simplified estimation equation is advisable for practical implications.

In early 1951, Brozek and Keys [104] published the first valid equations for men aged 18–55 years derived from SKF and body density (with UWW as the criteria method), although the SKF chosen were not ideal and their formula has not been widely used. Since body fat needed to be determined from body density, some representative authors, such as Siri [118] and Brozek [119], calculated formulas to estimate %BF. They obtained similar results in their conversion formulas (the difference between equations is less than 1% BF error of estimation).

To date, the generalized equations for predicting body fat from the sum of SKF, proposed by Durnin/Womersley [14] in 1974 for females and males aged 16–72 years; by Jackson/Pollock [15] in 1978 for males aged 18–61 years and; and by Jackson, Pollock, and Ward [16] in 1980 for females aged 18–55 years, are still widely used (see Supplementary Tables S7 and S8). Durnin/Womersley [14] tested different regression equations, based on the sum of one to four SKF. They assumed that there is little error using one, two, or three measured SKF, although the error can be reduced by using the sum of four SKF (*r* = 0.84–0.95). In fact, the Durnin/Womersley sum of four SKF equations is the most used [21, 24, 25, 30, 37, 39–41, 45, 50, 65, 68, 69, 73, 74, 77, 80, 85–88, 99–103]. Likewise, Jackson/Pollock [15] and Jackson, Pollock, and Ward[16] established models with the sum of three, four, and seven SKF, concluding that all of them were almost equally valid (*r* = 0.91–0.92 and *r* = 0.85–0.87 for male and females adults, respectively). This validity among different SKF sums was supported by later studies [30, 38, 82]. Therefore, when time is a handicap (i.e., in epidemiological studies or clinical settings), it seems that the most feasible is to apply one SKF (Durnin/Womersley) [14] or the sum of three-four SKF (Jackson/Pollock or Jackson, Pollock, and Ward)[15, 16].

Particularly, these equations showed high validity in general adult population [14–16, 30, 37–41, 50, 52, 55, 63, 65, 69, 73, 77, 80, 82, 84–86, 88, 100, 101, 103]. Only a few studies found low validity in these equations [39, 87, 100, 102]. Two of them found low validity in females with overweight [39] and adults with obesity [39, 100], in addition to Wattanapenpaiboon et al. [87], who suggest that Durnin/Womersley equation tends to increasingly underestimate %BF with increasing subcutaneous fat, being aware of some methodological limitations in adults with overweight or obesity, especially in females. Shafer et al. [102] reached the same conclusion regarding overestimation in female adults, although they found low validity even in adults with normal weight. Moreover, these seem to need a reevaluation within different race/ethnicity groups [40, 85, 86] or body fat status [30, 39]. Accordingly, although these equations are widely applicable, the SKF method has some drawbacks. Principally, SKF results may be affected by the expertise of the technician, the type of caliper used (e.g., most economic calipers have less scale precision), and some individual factors (e.g., adults with overweight or obesity, sex, or race/ethnicity).

Since then, several new equations based on anthropometric measurements or BIA analysis are still emerging and re-evaluated, resulting in an endless demand for comparisons between them, since regression equations seem to be population-specific, especially regarding sex, age, body fat status, or race ethnicity [40, 53, 65, 68, 74, 82, 109]. Specifically, BMI alone cannot be either generalized among different race ethnic groups [11, 13, 58, 80] and is age- and sex-dependent [11, 12, 57, 109]. Therefore, BMI equations used in combination with SKF [53, 62, 63] or WC and SKF [40, 45, 63, 81, 112] have been developed to improve its accuracy to estimate %BF. Regarding BAI [13] and despite race ethnicity or weight status differences, the studies in this systematic review concluded that BAI equations are more accurate than BMI alone to estimate %BF [7, 13, 59, 75, 84, 90]. Finally, although BIA is a method of easy applicability (as a field method), some studies have found that it underestimates or overestimates the estimation of %BF, mainly caused by derived equations based on race ethnicity, or specific calibration machine [80, 88, 94].

Finally, it seems that other lifestyle characteristics are rising to create the most specific equation to predict %BF. In 2001, Tucker et al. [43] developed and prediction equation that included diet and fitness and exercise questions, showing high validity (*r* = 0.89, *p* < 0.05). Similarly, Zanovec et al. [67] examined the role of physical activity in prediction equations based on BMI, suggesting that physical activity relied on the more precise and accurate equation to estimate %BF (in addition to sex and race ethnicity).

Nowadays, these equations are still being refined. In fact, Lee et al. [76] developed four equations where including from the simplest (i.e., age, height, weight, and WC) to the most complete (i.e., age, height, weight, WC, serum creatinine level, physical activity, smoking habit, and alcohol use), and unsurprisingly, they found the last one the most valid (*r* = 0.91, *p* < 0.001). Therefore, collecting information on lifestyle behavior should be worthy in designing a more efficient individual prediction equation.

The previous discussions have made clear that valid body composition estimation equations are applicable in clinical and epidemiological research and in health-related environments. Where possible, individualization is desirable. Otherwise, the generalized equation, such as the most simple versions of the Durnin/Womersley equation (one to three SKF), the Jackson/Pollock equation, and the Jackson, Pollock, and Ward equation (three or four SKF), is suitable for body composition estimation in adult population.

## Limitations and Strengths

Our systematic review has some limitations that should be acknowledged. Firstly, our data are representative of a healthy adult population. Despite we have included different weight statuses, race ethnicities, or activity levels, our data cannot be applied to other specific populations that may explain some of the discrepancies between our findings and those of other studies (e.g., athletes have less body fat than do non-athletes).

Another limitation of the present review comes from the fact that we could not conduct a meta-analysis owing to methodological heterogeneity such as different study designs, variability in adjustments for potential risk factors, and different statistical estimates. Although developing specific equations would be ideal, the more specific the population, the less general application an equation will have. Moreover, the more specific equation, the more difficult to implement that equation in epidemiologic, clinical, or field setting assessment. Furthermore, although an effort was made to classify the evidence based on different race ethnicities, following data reported or study site, many people could identify with more than one race ethnicity; therefore, the proposed categories should not be considered as absolute or viewed in isolation.

Finally, it is known that direct analysis of body composition can only be performed by chemical analysis of cadavers [120], and therefore, other methods serve as only indirect measurements of %BF, and all techniques suffer from some type of error. In fact, estimation equations imply theoretical assumptions of body composition measurement [120].

The strengths of the present study are supported by the large sample size sorted by sex, the wide age range of the sample, the inclusion of different race ethnics, different weight statuses, and the number of included field-based methods studied (*n* = 10) and derived equation studies (*n* = 90). In addition, we have investigated the validity of existing body composition field-based methods using the most precise or accurate criterion methods (i.e., “gold standards”) for the populations tested.

## Conclusions

The present systematic review emphasizes a number of important major points about the criterion-related validity of field-based methods and equations for body composition estimation in adults:

*Strong evidence* indicates that field-based methods, such as WC, BAI, and BMI, are valid indicators of body adiposity in general adult population. Moreover, classical equations, such as Durnin/Womersley equation; Jackson/Pollock equation; Jackson, Pollock, and Ward equation; and estimation equations implying SKF, alone or combined with circumferences, are most valid to estimate total body fat mass or %BF in general adult population. Therefore, these equations are valid to be “generalized” in the clinical or epidemiologic environments and field setting assessment. The ease in body composition assessment may help in the rapid identification, prevention, and management of the diverse health conditions related to obesity.

In practical terms, the best prediction equation should be chosen based on available time, existing resources, and characteristics of the sample studied. Future research should be aimed at (a) validating a suitable regression equation for use in each of the different BMI categories or race ethnicities or even lifestyle factors, such as nutritional status and fitness/exercise levels, and (b) reaching a consensus about which equations should be mainly used to homogenize research studies.

## Supplementary Information

Below is the link to the electronic supplementary material.

Supplementary file1 (DOCX 15 KB)

Supplementary file2 (DOCX 27 KB)

Supplementary file3 (DOCX 965 KB)

Supplementary file4 (DOCX 14 KB)

Supplementary file5 (DOCX 24 KB)

Supplementary file6 (DOCX 109 KB)

Supplementary file7 (DOCX 72 KB)

Supplementary file8 (DOCX 229 KB)

Supplementary file9 (DOCX 26 KB)

Supplementary file10 (DOCX 13 KB)

Supplementary file11 (DOCX 15 KB)

Supplementary file12 (PPTX 512 KB)
