# Supplementary material for: Criterion-Related Validity of Field-Based Methods and Equations for Body Composition Estimation in Adults: A Systematic Review
Source: Curr Obes Rep. 2022 Nov 11;11(4):336–49. doi: 10.1007/s13679-022-00488-8 (PMC9729144; doi:10.1007/s13679-022-00488-8)
Supplement: Supplementary file 1 — Supplementary file1 (DOCX 15 KB) [file 13679_2022_488_MOESM1_ESM.docx]

**Supplemental Material S1**

PubMed: all searches combined

(((((((Adult[Mesh:NoExp] OR "Middle Aged"[Mesh]) AND ("criterion validity" [All Fields] OR “criterion-related validity”[All Fields] OR validity[All Fields] OR validation[All Fields] OR estimation[All Fields] OR prediction [All Fields] OR cross-validation[All Fields]) AND (“body mass index ”[All Fields])))) OR (((Adult[Mesh:NoExp] OR "Middle Aged"[Mesh]) AND ("criterion validity" [All Fields] OR “criterion-related validity”[All Fields] OR validity[All Fields] OR validation[All Fields] OR estimation[All Fields] OR prediction [All Fields] OR cross-validation[All Fields]) AND (“waist circumference”[All Fields])) OR (((Adult[Mesh:NoExp] OR "Middle Aged"[Mesh]) AND ("criterion validity" [All Fields] OR “criterion-related validity”[All Fields] OR validity[All Fields] OR validation[All Fields] OR estimation[All Fields] OR prediction [All Fields] OR cross-validation[All Fields]) AND (“skin folds”[All Fields])) OR (((Adult[Mesh:NoExp] OR "Middle Aged"[Mesh]) AND ("criterion validity" [All Fields] OR “criterion-related validity”[All Fields] OR validity[All Fields] OR validation[All Fields] OR estimation[All Fields] OR prediction [All Fields] OR cross-validation[All Fields]) AND (“neck circumference”[All Fields])) OR (((Adult[Mesh:NoExp] OR "Middle Aged"[Mesh]) AND ("criterion validity" [All Fields] OR “criterion-related validity”[All Fields] OR validity[All Fields] OR validation[All Fields] OR estimation[All Fields] OR prediction [All Fields] OR cross-validation[All Fields]) AND (“hip circumference”[All Fields])) OR (((Adult[Mesh:NoExp] OR "Middle Aged"[Mesh]) AND ("criterion validity" [All Fields] OR “criterion-related validity”[All Fields] OR validity[All Fields] OR validation[All Fields] OR estimation[All Fields] OR prediction [All Fields] OR cross-validation[All Fields]) AND (“fat mass index” [All Fields])) OR (((Adult[Mesh:NoExp] OR "Middle Aged"[Mesh]) AND ("criterion validity" [All Fields] OR “criterion-related validity”[All Fields] OR validity[All Fields] OR validation[All Fields] OR estimation[All Fields] OR prediction [All Fields] OR cross-validation[All Fields]) AND ("body adiposity index" [All Fields])) OR (((Adult[Mesh:NoExp] OR "Middle Aged"[Mesh]) AND ("criterion validity" [All Fields] OR “criterion-related validity”[All Fields] OR validity[All Fields] OR validation[All Fields] OR estimation[All Fields] OR prediction [All Fields] OR cross-validation[All Fields]) AND ("bioelectrical impedance analysis" [All Fields])) OR (((Adult[Mesh:NoExp] OR "Middle Aged"[Mesh]) AND ("criterion validity" [All Fields] OR “criterion-related validity”[All Fields] OR validity[All Fields] OR validation[All Fields] OR estimation[All Fields] OR prediction [All Fields] OR cross-validation[All Fields]) AND ("waist to hip ratio" [All Fields])) OR (((Adult[Mesh:NoExp] OR "Middle Aged"[Mesh]) AND ("criterion validity" [All Fields] OR “criterion-related validity”[All Fields] OR validity[All Fields] OR validation[All Fields] OR estimation[All Fields] OR prediction [All Fields] OR cross-validation[All Fields]) AND ("waist to height ratio"[All Fields])))

Web of Sciences: all searches combined

(adult* OR "Middle Aged") AND ("criterion validity" OR "criterion-related validity" OR validity OR validation OR cross-validation OR estimation OR prediction) AND ("body mass index" OR "waist circumference" OR "skin folds" OR "neck circumference" OR "hip circumference" OR "fat mass index" OR "body adiposity index" OR "bioelectrical impedance analysis" OR "waist to hip ratio" OR "waist to height ratio")
