# Supplementary material for: Criterion-Related Validity of Field-Based Methods and Equations for Body Composition Estimation in Adults: A Systematic Review
Source: Curr Obes Rep. 2022 Nov 11;11(4):336–49. doi: 10.1007/s13679-022-00488-8 (PMC9729144; doi:10.1007/s13679-022-00488-8)
Supplement: Supplementary file 2 — Supplementary file2 (DOCX 27 KB) [file 13679_2022_488_MOESM2_ESM.docx]

**Supplementary Material S2.** List of excluded studies.

*a) Unable to source full text (n=10)*

1. Development of a Body Fat Prediction Equation for Young Adults. Faseb Journal. 2008;22.

2. Barnes JT, Wagganer JD, Loenneke JP, Miller WM, Abdul MMS, Soni DS. Validity Of A Joint Diameter-based System For The Measurement Of Body Composition. Medicine and Science in Sports and Exercise. 2015;47(5):41-.

3. Barnes JT, Wagganer JD, Loenneke JP, Miller WM, Gegg CR, Kylie WA, et al. Validity of Ultrasound and Skinfolds for the Measurement of Body Composition in Collegiate Baseball Players. Medicine and Science in Sports and Exercise. 2016;48(5):994-.

4. Heitmann BL. EVALUATION OF BODY-FAT ESTIMATED FROM BODY-MASS INDEX, SKINFOLDS AND IMPEDANCE - A COMPARATIVE-STUDY. European Journal of Clinical Nutrition. 1990;44(11):831-7.

5. Hirsch KR, Mock MG, Trexler ET, Blue MNM, Smith-Ryan AE. Validation Of A System-specific Dual-energy X-ray Absorptiometry-derived Body Volume Equation For 4-compartment Body Composition Calculations. Medicine and Science in Sports and Exercise. 2017;49(5):328-.

6. Kwon S. PERCENT BODY FAT PREDICTION EQUATIONS: EXTERNAL VALIDITY AND RACE/ETHNICITY. Medicine and Science in Sports and Exercise. 2011;43(3):550-.

7. Monsma EV, Torres-McGehee TM, Goins JM. Concurrent Validity of Tanita Scale and Skinfold Estimates of Percent Body Fat in Collegiate Athletes. Medicine and Science in Sports and Exercise. 2008;40(5):S274-S.

8. Shizgal HM. VALIDATION OF THE MEASUREMENT OF BODY-COMPOSITION FROM WHOLE-BODY BIOELECTRIC IMPEDANCE. Infusionstherapie Und Transfusionsmedizin. 1990;17:67-74.

9. Skoufas E, Kanellakis S, Apostolidou E, Khudokonenko V, Ziogou G, Papasotiriou I, et al. Validation of equation models estimating body composition in Greek population. Clinical nutrition ESPEN. 2016;13:e63-e4.

10. Zhao J, Huang C, Hong P, He Z, Wang M, Tian H, et al. Development And Validation Of Body Fat Prediction Equation In 20-69 Adults. Medicine and Science in Sports and Exercise. 2017;49(5):484-.

*b) Not relevant to criterion-related validity (n=35)*

1. Periodic health examination, 1994 update: 1. Obesity in childhood. Canadian Task Force on the Periodic Health Examination. CMAJ : Canadian Medical Association journal = journal de l'Association medicale canadienne. 1994;150(6):871-9.

2. Effectiveness of health checks conducted by nurses in primary care: final results of the OXCHECK study. Imperial Cancer Research Fund OXCHECK Study Group. Bmj. 1995;310(6987):1099-104.

3. Aasvee K, Rasmussen M, Kelly C, Kurvinen E, Giacchi MV, Ahluwalia N. Validity of self-reported height and weight for estimating prevalence of overweight among Estonian adolescents: the Health Behaviour in School-aged Children study. BMC Res Notes. 2015;8:606.

4. Abalkhail BA, Shawky S, Soliman NK. Validity of self-reported weight and height among Saudi school children and adolescents. Saudi Med J. 2002;23(7):831-7.

5. Basterra-Gortari FJ, Bes-Rastrollo M, Forga L, Martinez JA, Martinez-Gonzalez MA. [Validity of self-reported body mass index in the National Health Survey]. An Sist Sanit Navar. 2007;30(3):373-81.

6. Becroft L, Ooi G, Forsyth A, King S, Tierney A. Validity of multi-frequency bioelectric impedance methods to measure body composition in obese patients: a systematic review. International Journal of Obesity. 2019;43(8):1497-507.

7. Belahsen R, Mziwira M, Fertat F. Anthropometry of women of childbearing age in Morocco: body composition and prevalence of overweight and obesity. Public Health Nutrition. 2004;7(4):523-30.

8. Ben-Noun L, Sohar E, Laor A. Neck circumference as a simple screening measure for identifying overweight and obese patients. Obes Res. 2001;9(8):470-7.

9. Bielemann RM, Gonzalez MC, Barbosa-Silva TG, Orlandi SP, Xavier MO, Bergmann RB, et al. Estimation of body fat in adults using a portable A-mode ultrasound. Nutrition. 2016;32(4):441-6.

10. Burkhart TA, Arthurs KL, Andrews DM. Reliability of upper and lower extremity anthropometric measurements and the effect on tissue mass predictions. J Biomech. 2008;41(7):1604-10.

11. Chen YM, Ho SC, Lam SS, Chan SS. Validity of body mass index and waist circumference in the classification of obesity as compared to percent body fat in Chinese middle-aged women. Int J Obes (Lond). 2006;30(6):918-25.

12. Esco MR. The accuracy of the body adiposity index for predicting body fat percentage in collegiate female athletes. J Strength Cond Res. 2013;27(6):1679-83.

13. Goacher PJ, Lambert R, Moffatt PG. Can weight-related health risk be more accurately assessed by BMI, or by gender specific calculations of Percentage Body Fatness? Med Hypotheses. 2012;79(5):656-62.

14. Housh TJ, Johnson GO, Thorland WG, Cisar CJ, Hughes RA, Kenney KB, et al. Validity and intertester error of anthropometric estimations of body density. J Sports Med Phys Fitness. 1989;29(2):149-56.

15. KlipsteinGrobusch K, Georg T, Boeing H. Interviewer variability in anthropometric measurements and estimates of body composition. International Journal of Epidemiology. 1997;26:S174-S80.

16. Kuehnapfel A, Ahnert P, Loeffler M, Broda A, Scholz M. Reliability of 3D laser-based anthropometry and comparison with classical anthropometry. Scientific Reports. 2016;6.

17. Quiroz-Olguin G, Serralde-Zuniga AE, Saldana-Morales V, Guevara-Cruz M. Validation of a new formula for predicting body weight in a Mexican population with overweight and obesity. Nutr Hosp. 2013;28(3):690-3.

18. Schutz Y, Sarafian D, Miles JL, Montani JP, Dulloo AG. Non-contact assessment of waist circumference: will tape measurements become progressively obsolete? Eur J Clin Nutr. 2012;66(2):269-72.

19. Smith-Ryan AE, Blue MNM, Trexler ET, Hirsch KR. Utility of ultrasound for body fat assessment: validity and reliability compared to a multicompartment criterion. Clin Physiol Funct Imaging. 2018;38(2):220-6.

20. Belarmino G, Torrinhas RS, Sala P, Horie LM, Damiani L, Lopes NC, et al. A new anthropometric index for body fat estimation in patients with severe obesity. BMC obesity. 2018;5:25-.

21. Chumlea WC, Guo SS. Assessment and prevalence of obesity - Application of new methods to a major problem. Endocrine. 2000;13(2):135-42.

22. Deurenberg P, Andreoli A, Borg P, Kukkonen-Harjula K, de Lorenzo A, van Marken Lichtenbelt WD, et al. The validity of predicted body fat percentage from body mass index and from impedance in samples of five European populations. Eur J Clin Nutr. 2001;55(11):973-9.

23. Di Lorenzo N, Servidio M, Di Renzo L, Orlandi C, Coscarella G, Gaspari A, et al. Is digital image plethysmographic (DIP) acquisition a valid new tool for preoperative body composition assessment? A validation by dual-energy X-ray absorptiometry. Obes Surg. 2006;16(5):560-6.

24. Direk K, Cecelja M, Astle W, Chowienczyk P, Spector TD, Falchi M, et al. The relationship between DXA-based and anthropometric measures of visceral fat and morbidity in women. BMC Cardiovasc Disord. 2013;13:25.

25. Fuller NJ, Hardingham CR, Graves M, Screaton N, Dixon AK, Ward LC, et al. Assessment of limb muscle and adipose tissue by dual-energy X-ray absorptiometry using magnetic resonance imaging for comparison. Int J Obes Relat Metab Disord. 1999;23(12):1295-302.

26. Goran MI, Khaled MA. Cross-validation of fat-free mass estimated from body density against bioelectrical resistance: effects of obesity and gender. Obes Res. 1995;3(6):531-9.

27. Harbin MM, Kasak A, Ostrem JD, Dengel DR. Validation of a three-dimensional body scanner for body composition measures. Eur J Clin Nutr. 2018;72(8):1191-4.

28. Hemmingsson E, Udden J, Neovius M. No apparent progress in bioelectrical impedance accuracy: validation against metabolic risk and DXA. Obesity (Silver Spring). 2009;17(1):183-7.

29. Henry CJ, Ponnalagu S, Bi X. Development of an Easy-to-Use Visual Aid for the Prediction of Body Fat Based on Waist Circumference and Height in Asian Chinese Adults. Journal of the Academy of Nutrition and Dietetics. 2019;119(9):1533-40.

30. Henry CJ, S DOP, Bi X, Tan SY. New Equations to Predict Body Fat in Asian-Chinese Adults Using Age, Height, Skinfold Thickness, and Waist Circumference. J Acad Nutr Diet. 2018;118(7):1263-9.

31. Jebb SA, Siervo M, Murgatroyd PR, Evans S, Fruhbeck G, Prentice AM. Validity of the leg-to-leg bioimpedance to estimate changes in body fat during weight loss and regain in overweight women: a comparison with multi-compartment models. Int J Obes (Lond). 2007;31(5):756-62.

32. LaForgia J, Dollman J, Dale MJ, Withers RT, Hill AM. Validation of DXA body composition estimates in obese men and women. Obesity (Silver Spring). 2009;17(4):821-6.

33. Leiter LA, Lukaski HC, Kenny DJ, Barnie A, Camelon K, Ferguson RS, et al. THE USE OF BIOELECTRICAL-IMPEDANCE ANALYSIS (BIA) TO ESTIMATE BODY-COMPOSITION IN THE DIABETES CONTROL AND COMPLICATIONS TRIAL (DCCT). International Journal of Obesity. 1994;18(12):829-35.

34. McLester CN, Nickerson BS, Kliszczewicz BM, Hicks CS, Williamson CM, Bechke EE, et al. Validity of DXA body volume equations in a four-compartment model for adults with varying body mass index and waist circumference classifications. PLoS One. 2018;13(11):e0206866.

35. Varady KA, Santosa S, Jones PJH. Validation of hand-held bioelectrical impedance analysis with magnetic resonance imaging for the assessment of body composition in overweight women. American Journal of Human Biology. 2007;19(3):429-33.

*c) No language inclusion criteria (n=1)*

1. Rech CR, Lima LRAd, Cordeiro BA, Petroski EL, Vasconcelos FdAGd. Validade de equações antropométricas para a estimativa da gordura corporal em idosos do sul do Brasil. Revista Brasileira de Cineantropometria & Desempenho Humano. 2010;12(1):01-7.

*d) No healthy participants (n=7)*

1. Buchholz AC, McGillivray CF, Pencharz PB. The use of bioelectric impedance analysis to measure fluid compartments in subjects with chronic paraplegia. Arch Phys Med Rehabil. 2003;84(6):854-61.

2. Charatsi AM, Dusser P, Freund R, Maruani G, Rossin H, Boulier A, et al. Bioelectrical impedance in young patients with cystic fibrosis: Validation of a specific equation and clinical relevance. J Cyst Fibros. 2016;15(6):825-33.

3. El Ghoch M, Alberti M, Milanese C, Battistini NC, Pellegrini M, Capelli C, et al. Comparison between dual-energy X-ray absorptiometry and skinfolds thickness in assessing body fat in anorexia nervosa before and after weight restoration. Clinical Nutrition. 2012;31(6):911-6.

4. Esco MR, Nickerson BS, Bicard SC, Russell AR, Bishop PA. Agreement of BMI-Based Equations and DXA in Determining Body Fat Percentage in Adults With Down Syndrome. Adapted Physical Activity Quarterly. 2016;33(1):89-96.

5. Hughes JT, Maple-Brown LJ, Piers LS, Meerkin J, O'Dea K, Ward LC. Development of a single-frequency bioimpedance prediction equation for fat-free mass in an adult Indigenous Australian population. Eur J Clin Nutr. 2015;69(1):28-33.

6. Perea V, Jimenez A, Flores L, Ortega E, Coves MJ, Vidal J. Anthropometric indexes outperform bioelectrical impedance analysis-derived estimates of body composition in identification of metabolic abnormalities in morbid obesity. Surg Obes Relat Dis. 2013;9(5):648-52.

7. Rodriguez-Escudero JP, Pack QR, Somers VK, Thomas RJ, Squires RW, Sochor O, et al. Diagnostic Performance of Skinfold Method to Identify Obesity as Measured by Air Displacement Plethysmography in Cardiac Rehabilitation. Journal of Cardiopulmonary Rehabilitation and Prevention. 2014;34(5):335-42.

*e) Specific sport criteria (n=8)*

1. Andreoli A, Melchiorri G, Volpe SL, Sardella F, Iacopino L, De Lorenzo A. Multicompartment model to assess body composition in professional water polo players. Journal of Sports Medicine and Physical Fitness. 2004;44(1):38-43.

2. Modlesky CM, Cureton KJ, Lewis RD, Prior BM, Sloniger MA, Rowe DA. Density of the fat-free mass and estimates of body composition in male weight trainers. J Appl Physiol (1985). 1996;80(6):2085-96.

3. Munguia-Izquierdo D, Suarez-Arrones L, Di Salvo V, Paredes-Hernandez V, Ara I, Mendez-Villanueva A. Estimating fat-free mass in elite youth male soccer players: cross-validation of different field methods and development of prediction equation. Journal of Sports Sciences. 2019;37(11):1197-204.

4. Riyahi-Alam S, Mansournia MA, Kabirizadeh Y, Mansournia N, Steyerberg E, Kordi R. Development and Validation of a Skinfold Model for Estimation of Body Density for a Safe Weight Reduction in Young Iranian Wrestlers. Sports Health. 2017;9(6):564-9.

5. Santos DA, Silva AM, Matias CN, Magalhaes JP, Minderico CS, Thomas DM, et al. Utility of novel body indices in predicting fat mass in elite athletes. Nutrition. 2015;31(7-8):948-54.

6. Slater GJ, Duthie GM, Pyne DB, Hopkins WG. Validation of a skinfold based index for tracking proportional changes in lean mass. Br J Sports Med. 2006;40(3):208-13.

7. Stewart AD, Hannan WJ. Prediction of fat and fat-free mass in male athletes using dual X-ray absorptiometry as the reference method. Journal of Sports Sciences. 2000;18(4):263-74.

8. Withers RT, Craig NP, Bourdon PC, Norton KI. Relative body fat and anthropometric prediction of body density of male athletes. Eur J Appl Physiol Occup Physiol. 1987;56(2):191-200.

*f) Reviews excluded, not relevant to criterion-related validity (n=2)*

1. Arias Tellez MJ, Martinez-Tellez B, Soto J, Sanchez-Delgado G. Validity of neck circumference as a marker of adiposity in children and adolescents, and in adults: a systematic review. Nutr Hosp. 2018;35(3):707-21.

2. Deurenberg P, Yap M, van Staveren WA. Body mass index and percent body fat: a meta analysis among different ethnic groups. Int J Obes Relat Metab Disord. 1998;22(12):1164-71.
