# Supplementary material for: Criterion-Related Validity of Field-Based Methods and Equations for Body Composition Estimation in Adults: A Systematic Review
Source: Curr Obes Rep. 2022 Nov 11;11(4):336–49. doi: 10.1007/s13679-022-00488-8 (PMC9729144; doi:10.1007/s13679-022-00488-8)
Supplement: Supplementary file 3 — Supplementary file3 (DOCX 965 KB) [file 13679_2022_488_MOESM3_ESM.docx]

**Supplementary Material S3.** Results of validity of field-based body composition estimation and estimation equations for body composition in adults.

The criterion methods used to compare with field-based methods were DXA^17, 30-32, 69, 82, 95, 110, 112^ (n= 9) and UWW^33^ (n= 1). The field-based methods used were BMI^30-32, 69, 95, 112^ (n= 6), WC^31, 69, 95, 110, 112^ (n= 4), BAI (n= 3)^32, 69, 82^, HC^69, 95^ (n= 2), SKF^33^ (n= 1), BIA^110^ (n= 1), WHR^95^ (n= 1), and WHtR^110^ (n= 1).

The following field-based methods showed **very high/high validity**, compared with criterion methods: BMI (derived from five studies, r= 0.71-0.95; all, p<0.05)^30-32, 69, 95^; WC (derived from three studies, r= 0.72-0.89; all, p<0.05)^31, 95, 110^; HC (derived from one study, r= 0.77-0.85, p<0.0001)^95^; SKF (derived from one study, r= 0.88-0.95, p<0.001)^33^; BAI (derived from one study, r= 0.70-0.81, p<0.05)^32^; BIA (derived from one study, r= 0.99, p<0.001)^110^ and; WHtR (derived from one study, r= 0.82, p<0.001)^110^.

The following field-based methods showed **moderate validity**, compared with criterion methods: WC (derived from two studies, r= 0.57-0.63; both, p<0.001)^69, 110^; BAI (derived from two studies, r= 0.58-0.68; both, p<0.001)^69, 82^; BMI (derived from two studies, r= 0.54-0.69; both, p<0.01)^32, 69^; HC (derived from one study, r= 0.66, p<0.0001)^69^; BIA (derived from one study, r= 0.59, p<0.001)^110^; WHR (derived from one study, r=0.51-0.63, p<0.001)^95^ and; WHtR (derived from one study, r= 0.59, p<0.001)^110^.

The following field-based methods showed **very low/low validity**, compared with criterion methods: WC (derived from one study, r= 0.23, p<0.05)^112^; BMI (derived from one study, r= 0.22, p<0.005)^112^ and; WHR (derived from one study, r= 0.46, p<0.05)^95^.

There was a balanced distribution in the female-male ratio between studies and the validity of the results does not change significantly between sexes.

***Age groups***

**The 19-33 years old group:** WC showed **high validity** (derived from two studies, r= 0.83-0.86; both, p<0.05)^31, 110^, and **moderate validity** (derived from one study, r= 0.57, p<0.001)^110^; WHtR showed **high validity** (derived from one study, r= 0.75, p<0.001)^110^, and **moderate validity** (derived from one study, r= 0.59, p<0.001)^110^; BIA showed **very high validity** (derived from one study, r= 0.99, p<0.001)^110^, and **moderate validity** (derived from one study, r= 0.59, p<0.001)^110^ and; BMI showed **high validity** (derived from one study, r= 0.79-0.84, p<0.05)^31^.

**The 34-49 years old group**: WC showed **high validity** (derived from one study, r= 0.76-0.77, p<0.05)^31^, and **low validity** (derived from one study, r= 0.23, p<0.05)^112^; BMI showed **high validity** (derived from one study, r= 0.75-0.79, p<0.05)^31^, and **very low validity** (derived from one study, r= 0.22, p<0.05)^112^ and; SKF showed **very high/high validity** (derived from one study, r= 0.88-0.95, p<0.05)^33^.

**The 50-64 years old group:** BMI showed **very high/high validity** (derived from five studies, r= 0.71-0.95; all, p<0.05)^30-32, 69, 95^; WC showed **very high/high validity** (derived from three studies, r= 0.72-0.90; all, p<0.05)^31, 69, 95^, and **moderate validity** (derived from one study, r= 0.63, p<0.0001)^69^; BAI showed **high validity** (derived from one study, r= 0.72, p<0.01)^32^ and **moderate validity** (derived from two studies, r= 0.55-0.65; both, p<0.01)^69, 82^; HC showed **high validity** (derived from one study, r= 0.77-0.85, p<0.05)^95^, and **moderate validity** (derived from one study, r= 0.66, p<0.0001)^69^ and; WHR showed **moderate validity** (derived from one study, r= 0.46-0.63, p<0.05)^95^.

***Weight status groups***

**Group with normal weight:** WC showed **high validity** (derived from one study, r= 0.72, p<0.0001)^69^, **moderate validity** (derived from two studies, r= 0.57-0.63; both, p<0.001)^69, 110^, and **low validity** (derived from one study, r= 0.23, p<0.05)^112^; BMI showed **high validity** (derived from two studies, r= 0.70-0.84; both, p<0.05)^30, 69^, and **very low** (derived from one study, r= 0.22, p<0.05)^112^; SKF showed **high/very high validity** (derived from one study, r= 0.88-0.95, p<0.001)^33^; HC showed **moderate validity** (derived from one study, r= 0.66, p<0.0001)^69^; WHtR showed **moderate** **validity** (derived from one study, r= 0.59, p<0.001)^110^; BAI showed **moderate** **validity** (derived from one study, r= 0.58-0.62, p<0.001)^69^ and; BIA showed **moderate validity** (derived from one study, r= 0.59, p<0.001)^110^.

**Group with overweight:** WC showed **very high/high validity** (derived from two studies, r= 0.84-0.90; both, p<0.001)^95, 110^; BMI showed **very high/high validity** (derived from two studies, r= 0.71-0.95; both, p<0.05)^32, 95^; BAI showed **high validity** (derived from one study, r= 0.72, p<0.01)^32^ and **moderate validity** (derived from two studies, r= 0.55-0.65; both, p<0.001)^32, 82^; HC showed **high validity** (derived from one study, r= 0.77-0.85, p<0.001)^95^; WHtR showed **high validity** (derived from one study, r= 0.75, p<0.001)^110^; BIA showed **very high validity** (derived from one study, r= 0.99, p<0.001)^110^ and; WHR showed **moderate** **validity** (derived from one study, r= 0.46-0.63, p<0.001)^95^.

**Group** **with obesity**: WC showed **high validity** (derived from one study, r= 0.84, p<0.001)^110^ and; WHtR showed **high validity** (derived from one study, r= 0.75, p<0.001)^110^.

***Race-ethnicity groups***

**Caucasian/White** **adults:** BMI showed **high validity** (derived from two studies, r= 0.78-0.84; both, p<0.05)^30, 31^ and **moderate validity** (derived from one study, r= 0.54, p<0.01)^32^; WC showed **high validity** (derived from two studies, r= 0.83-0.84; both, p<0.05)^31, 110^ and **moderate validity** (derived from one study, r= 0.57, p<0.001)^110^; WHtR showed **high validity** (derived from one study, r= 0.75, p<0.001)^110^ and **moderate validity** (derived from one study, r= 0.59, p<0.001)^110^; BIA showed **very high/high validity** (derived from one study, r= 0.99, p<0.001)^110^ and **moderate validity** (derived from one study, r= 0.59, p<0.001)^110^; SKF showed **very high/high validity** (derived from one study, r= 0.88-0.95, p<0.05)^33^ and; BAI showed **high validity** (derived from one study, r= 0.70, p<0.01)^32^.

**African-American/Black** **adults:** WC showed **high validity** (derived from one study, r= 0.84, p<0.05)^31^; BAI showed **high validity** (derived from one study, r= 0.81, p<0.01)^32^; BMI showed **high validity** (derived from one study, r= 0.78, p<0.05)^31^ and **moderate validity** (derived from one study: r= 0.62, p<0.01)^32^.

**Other different races-ethnicities**: In Tasmanian adults, WC, HC and BMI showed **very high/high validity** (r= 0.77-0.90, p<0.0001)^95^; in Hispanic, WC and BMI showed **high validity** (r= 0.74-0.78, p<0.05)^31^; in Chinese adults, WC and BMI showed **moderate** to **high validity** (r= 0.63-0.72, p<0.0001)^69^, and HC and BAI showed **moderate** validity (0.58-0.66, p<0.0001)^69^; in Brazilian adults, BAI showed **moderate validity** (r= 0.65, p<0.001)^82^; in Korean adults, WC and BMI showed **low validity** (r= 0.23-0.39, p<0.05)^112^.

**Results of validity of estimation equations for body composition in adults.**

The criterion methods used to compare with field-based methods, in the estimation equations studies were: DXA^6, 10, 21-23, 34-39, 42-51, 53-55, 58, 60, 66, 71, 73-75, 77, 80, 83, 86, 88, 89, 92-94, 98, 100, 111^ (n= 44), UWW^11-13, 18-20, 24-26, 28, 29, 40, 49, 52, 54, 55, 59, 61-63, 65, 68, 70, 72, 81, 84, 85, 91, 102-108^ (n= 35), D_2_O^54, 55, 65, 76, 79, 87, 90^ (n= 7), ADP^27, 56, 67, 78, 101, 109^ (n= 6), isotope dilution^57, 64, 96^ (n= 3), ^3^H_2_O ^54, 55^ (n= 2), K40^57, 96^ (n= 2), MRI^41, 99^ (n= 2), and photon absorptiometry^65^ (n= 1). The field-based methods used were: SKF^11-13, 18-22, 24-29, 34-39, 42, 43, 45-53, 57, 60, 62-65, 67, 68, 70-73, 76, 79-81, 83-87, 90, 91, 94, 96, 98-107, 109, 111^ (n= 66), WC^6, 10, 12, 19, 26, 35, 36, 38-42, 44-48, 51, 53, 54, 57, 60, 62, 64, 71, 73-75, 78, 80, 83, 85, 88, 90-92, 94, 96, 98, 105, 106, 111^ (n= 42), HC^10, 26, 28, 29, 35, 40-42, 44-48, 51, 53, 54, 59, 62, 64, 71-74, 78, 80, 84, 85, 88, 90, 91, 94, 104^ (n= 32), BMI^36, 39, 46, 48-50, 53, 54, 56, 58, 60-62, 64, 66, 68, 71, 74-76, 83, 88, 89, 93, 96, 108^ (n= 26), arm circumference^11, 18-20, 25, 26, 28, 29, 38-40, 47, 51, 57, 59, 60, 62, 72, 85, 91, 94, 96, 104-106^ (n= 25), thigh circumference^11, 18-20, 25, 28, 29, 38-40, 42, 47, 51, 57, 59, 60, 62, 72, 80, 85, 91, 94, 104-106^ (n= 25), AbC^18, 23, 25, 28, 29, 38, 47, 51, 59, 64, 85, 88, 91, 98, 103-106^ (n= 18), chest circumference^18, 19, 25, 26, 28, 29, 38, 40, 42, 47, 51, 57, 59, 60, 103-106^ (n= 18), BIA^6, 21, 34, 52, 68, 70, 77-79, 81, 83, 86, 87, 93, 99, 100, 102, 111^ (n= 18), calf circumference^11, 18, 20, 25, 38-40, 47, 51, 59, 72, 80, 85, 91, 104-106^ (n= 17), forearm circumference^12, 18, 25, 28, 29, 38, 47, 51, 104-106^ (n= 11), NC^18, 23, 26, 28, 29, 38, 40, 51, 59, 98^ (n= 10), bi-acromial diameter^20, 25, 28, 29, 57, 59, 104-106^ (n= 9), bi-iliac diameter^20, 25, 28, 29, 57, 59, 104-106^ (n= 9), WHR^44, 53, 54, 56, 62, 64, 78, 83^ (n= 8), ankle circumference^25, 28, 29, 57, 59, 104-106^ (n= 8), shoulder circumference^18, 25, 28, 29, 59, 104-106^ (n= 8), chest diameter^25, 28, 29, 57, 59, 104-106^ (n= 8), bitrochanteric diameter^25, 28, 29, 59, 104-106^ (n= 7), knee diameter^25, 28, 29, 59, 104-106^ (n= 7), wrist diameter^25, 28, 29, 59, 104-106^ (n= 7), gluteal circumference^13, 19, 25, 96, 105, 106^ (n= 6), wrist circumference^18, 25, 57, 59, 105, 106^ (n= 6), WHtR^36, 56, 88, 92, 93^ (n= 5), BAI^10, 56, 74, 83, 89^ (n= 5), head circumference^25, 28, 29, 59^ (n= 4), ankle diameter^25, 28, 29, 59^ (n= 4), elbow diameter^25, 28, 29, 59^ (n= 4), knee circumference^25, 28, 29^ (n= 3), bideltoid diameter^28, 29, 59^ (n= 3), deltoid circumference^28, 29^ (n= 2), arm diameter^28, 29^ (n= 2), foot length^28, 29^ (n= 2), hand length^28, 29^ (n= 2), arm span^62^ (n= 1), bone breadths^42^ (n= 1), cup size^106^ (n= 1), leg length^62^ (n=1) and tibia/total body length^54^ (n=1). Whenever applicable, the total body density was converted to %BF by Siri^11-13, 18-21, 24, 27-29, 34-38, 43, 45, 59, 61, 65, 67, 76, 79, 83-85, 91, 99-101, 105-108^ (n= 35), by Brozek^11-13, 19, 20, 25, 26, 28, 29, 35, 45, 52, 63, 64, 70, 72, 76, 99, 102, 104-106^ (n= 22), and by Rathbun and Pace^19, 28, 29, 103^ (n= 4).

Among the studies analyzing the criterion-related validity of the equations proposed, those by Durnin/Womersley^11, 18, 21, 22, 27, 34, 36-38, 42, 47, 49, 62, 64, 67, 68, 72, 73, 76, 79, 84-87, 99-102^ (n= 28), by Jackson, Pollock and Ward^13, 27, 34, 35, 38, 49, 62, 72, 81, 83-85^ (n= 12), and by Jackson/Pollock^12, 34, 49, 52, 62, 72, 83^ (n= 7) were the most common. Due to the large number of proposed equations, only the most common ones, at least cited in three studies, were analysed for validity.

The following classical equations showed **very high/high validity** to estimate total body fat mass or BF%, compared with criterion methods: Durnin/Womersley equation (derived from 18 studies, r= 0.71-0.95; all, p<0.05)^11, 27, 34, 36-38, 47, 62, 64, 68, 72, 76, 79, 85, 87, 99, 100, 102^; Jackson, Pollock and Ward equation (derived from 10 studies, r= 0.71-0.97; all, p<0.05)^13, 34, 35, 49, 62, 72, 81, 83-85^ and; Jackson/Pollock equation (derived from six studies, r= 0.71-0.98; all, p<0.05)^12, 34, 49, 52, 62, 83^.

Moreover, the following estimation equations and indexes showed **very high/high validity** to estimate total body fat mass or BF%, compared with criterion methods: BMI equations (derived from 16 studies, r= 0.71-0.90; all, p<0.05)^36, 45, 53-56, 58, 61, 66, 68, 74, 83, 88, 89, 96, 108^; BIA equations (derived from 14 studies, r= 0.71-0.99; all, p<0.05)^34, 44, 52, 68, 70, 77-79, 83, 87, 93, 99, 100, 102^; equations implying sum of SKF (derived from 10 studies, r= 0.71-0.95; all, p<0.05)^34, 36, 47, 63, 65, 70, 81, 103, 107, 109^; equations combining circumferences+SKF (derived from nine studies, r= 0.75-0.97; all, p<0.05)^39, 42, 46, 48, 73, 94, 96, 104, 105^; equations based on WC (derived from seven studies, r= 0.72-0.89; all, p<0.05)^6, 36, 39, 41, 83, 90, 92^; equations combining WC+SKF (derived from five studies, r= 0.80-0.99; all, p<0.05)^37, 42, 62, 80, 111^; BAI equations (derived from five studies, r= 0.72-0.85; all, p<0.05)^10, 56, 74, 83, 89^; equations combining various circumferences (derived from four studies, r= 0.80-0.93; all, p<0.05)^39, 46, 59, 64^; WHtR equations (derived from four studies, r= 0.72-0.87; all, p<0.05)^36, 88, 90, 92^; equations combining AbC+SKF (derived from three studies, r= 0.84-0.97; all, p<0.05)^47, 51, 85^; equations combining WC+SKF+BMI (derived from three studies, r= 0.82-0.91; all, p<0.05)^44, 53, 71^; equations combining circumferences+diameters+SKF (derived from three studies, r= 0.75-0.99; all, p<0.05)^57, 105, 106^; equations combining SKF+BMI (derived from three studies, r= 0.81-0.93; all, p<0.05)^50, 60, 62^; equations combining abdominal circumference (AbC)+NC (derived from two studies, r= 0.80-0.90; both, p<0.05)^59, 98^ and; WHR equations (derived from one study, r= 0.82-0.84, p<0.05)^56^.

The following classical equations showed **moderate validity** to estimate total body fat mass or BF%, compared with criterion methods: Durnin/Womersley equation (derived from six studies, r= 0.50-0.67; all, p<0.001)^36, 42, 67, 84, 99, 100^; and Jackson/Pollock equation (derived from one study, r= 0.67, p<0.01)^72^. Moreover, the following estimation equations and indexes showed **moderate validity** to estimate total body fat mass or BF%, compared with criterion methods: BMI equations (derived from seven studies, r= 0.50-0.68; all, p<0.05)^6, 36, 49, 54, 55, 68, 108^; BIA equations (derived from three studies, r= 0.54-0.69, all, p<0.01)^68, 86, 99^; equations based on WC (derived from two studies, r= 0.56-0.64; both, p<0.05)^6, 45^; equations implying sum of SKF (derived from one study, r= 0.52, p<0.05)^45^; BAI equations (derived from one study, r= 0.56-0.64; all, p<0.05)^6^ and; WRH equations (derived from one study, r= 0.55-0.65, p<0.05)^83^.

The following classical equation showed **very low/low validity** to estimate total body fat mass or BF%, compared with criterion methods: Durnin/Womersley equation (derived from four studies, r= 0.00-0.44; all, p<0.14)^36, 86, 99, 101^. Moreover, the following estimation equations and indexes showed **very low/low validity** to estimate total body fat mass or BF%, compared with criterion methods, WHR equations (derived from two studies, r= 0.21-0.30; both, p<0.05)^53, 90^; BAI equations (derived from one study, r= 0.35, p<0.05)^56^; BMI equations (derived from one study, r= 0.47, p<0.05)^108^ and; WHtR equations (derived from one study, r= 0.47-0.49, p<0.05)^56^.

Different validity has been found between female and male populations, specific age groups (19-33 years, 34-49 years, and 50-64 years), BMI status (group with normal weight, with overweight and with obesity), and race-ethnicity.

***Sex groups***

**Female adults:** Durnin/Womersley equation showed **very high/high validity** (derived from 14 studies, r= 0.71-0.95; all, p<0.05)^11, 34, 37, 38, 47, 62, 64, 68, 72, 76, 85, 87, 99, 102^, **moderate validity** (derived from three studies, r= 0.50-0.63; all, p<0.05)^67, 84, 100^, and **low validity** (derived from two studies, r= 0.00-0.44; both, p<0.05)^86, 101^; Jackson, Pollock and Ward equation showed **very high/high validity** (derived from 10 studies, r= 0.71-0.93; all, p<0.05)^13, 34, 35, 49, 62, 72, 81, 83-85^; BMI equations showed **very high/high validity** (derived from 15 studies, r= 0.71-0.90; all, p<0.05)^36, 45, 49, 54-56, 58, 61, 68, 74, 76, 83, 88, 89, 108^ and **moderate validity** (derived from two studies, r= 0.55-0.68; both, p<0.05)^6, 55^; BIA equations showed **very high/high validity** (derived from 11 studies, r= 0.76-0.99; all, p<0.05)^34, 44, 68, 77, 78, 83, 87, 93, 99, 100, 102^ and **moderate validity** (derived from one study, r= 0.56, p<0.0001)^86^; equations combining circumferences+SKF showed **high validity** (derived from eight studies, r= 0.82-0.97; all, p<0.05)^39, 42, 46, 48, 73, 94, 96, 104^; equations implying sum of SKF showed **very high/high validity** (derived from six studies, r= 0.75-0.92; all, p<0.05)^34, 36, 65, 81, 107, 109^ and **moderate validity** (derived from one study, r= 0.52, p<0.05)^45^; BAI equations showed showed **high validity** (derived from five studies, r= 0.76-0.85; all, p<0.05)^10, 56, 74, 83, 89^ and **moderate validity** (derived from one study, r= 0.56, p<0.05)^6^; equations based on WC showed **high validity** (derived from four studies, r= 0.74-0.84; all, p<0.05)^36, 39, 83, 92^ and **moderate validity** (derived from two studies, r= 0.56-0.64; both, p<0.05)^6, 45^; equations combining various circumferences showed **very high/high validity** (derived from four studies, r= 0.80-0.93; all, p<0.05)^39, 46, 59, 64^; equations combining AbC+SKF showed **high validity** (derived from three studies, r= 0.85-0.94; all, p<0.05)^47, 51, 85^; equations combining SKF+BMI showed **high validity** (derived from three studies, r= 0.85-0.89; all, p<0.05)^50, 60, 62^; WHtR equations showed **high validity** (derived from three studies, r= 0.74-0.85; all, p<0.05)^36, 88, 92^; equations combining WC+SKF showed **very high/high validity** (derived from two studies, r= 0.93-0.98; both, p<0.05)^80, 111^; equations combining WC+SKF+BMI showed **high validity** (derived from two studies, r= 0.87-0.90; both, p<0.05)^44, 53^; equations combining circumferences+diameters+SKF showed **very high validity** (derived from two studies, r= 0.92-0.99; both, p<0.05)^57, 106^; WHR equations showed **very high/high validity** (derived from one study, r= 0.82-0.84, p<0.05)^56^ and **moderate validity** (derived from one study, r= 0.55, p<0.05)^83^ and; equations combining AbC+NC showed **very high validity** (derived from one study, r= 0.81, p<0.05)^98^.

**Male adults:** Durnin/Womersley equation showed **very high/high validity** (derived from 11 studies, r= 0.70-0.95; all, p<0.05)^11, 34, 37, 47, 62, 68, 72, 76, 79, 100, 102^ and **low validity** (derived from two studies, r= 0.00-0.44; both, p<0.05)^86, 101^; Jackson/Pollock equation showed **very high/high validity** (derived from six studies, r= 0.86-0.92; all, p<0.05)^12, 34, 49, 52, 62, 83^ and **moderate validity** (derived from one study, r= 0.67, p<0.01)^72^;BMI equations showed **very high/high validity** (derived from 12 studies, r= 0.72-0.90; all, p<0.05)^36, 54-56, 58, 61, 74, 76, 83, 88, 89, 108^ and **moderate validity** (derived from six studies, r= 0.61-0.68; all, p<0.05)^6, 36, 49, 54, 55, 68^; equations implying sum of SKF showed **very high/high validity** (derived from nine studies, r= 0.70-0.95; all, p<0.05)^34, 36, 47, 63, 65, 70, 103, 107, 109^; BIA equations showed **very high/high validity** (derived from nine studies, r= 0.71-0.99; all, p<0.05)^34, 52, 70, 78, 79, 83, 93, 100, 102^ and **moderate validity** (derived from two studies, r= 0.56-0.69; both, p<0.05)^68, 86^; equations based on WC showed **high validity** (derived from seven studies, r= 0.73-0.89; all, p<0.05)^6, 36, 39, 41, 83, 90, 92^; equations combining circumferences+SKF showed **high validity** (derived from five studies, r= 0.82-0.96; all, p<0.05)^39, 46, 73, 94, 105^; BAI equations showed **high validity** (derived from four studies, r= 0.72-0.85; all, p<0.05)^10, 74, 83, 89^, **moderate validity** (derived from one study, r= 0.64, p<0.05)^6^, and **low validity** (derived from one study, r= 0.35, p<0.05)^56^; equations combining WC+SKF showed **very high/high validity** (derived from four studies, r= 0.86-0.93; all, p<0.05)^42, 62, 80, 111^; WHtR equations showed **high validity** (derived from four studies, r= 0.72-0.87; all, p<0.05)^36, 88, 90, 92^; equations combining AbC+NC showed **very high validity** (derived from two studies, r= 0.88-0.90; both, p<0.05)^59, 98^; equations combining WC+SKF+BMI showed **high validity** (derived from two studies, r= 0.82-0.91; both, p<0.05)^53, 71^; equations combining various circumferences showed **very high/high validity** (derived from two studies, r= 0.86-0.92; both, p<0.05)^39, 46^; equations combining circumferences+diameters+SKF showed **very high validity** (derived from two studies, r= 0.94-0.99; both, p<0.05)^57, 105^; equations combining SKF+BMI showed **high validity** (derived from two studies, r= 0.82-0.84; both, p<0.05)^50, 60^; WHR equations showed **high validity** (derived from one study, r= 0.84, p<0.05)^56^, **moderate validity** (derived from one study, r= 0.65, p<0.05)^83^, and **low validity** (derived from one study, r= 0.33, p<0.05)^90^ and; equations combining AbC+SKF showed **high validity** (derived from one study, r= 0.97, p<0.001)^51^.

***Age groups***

**The 19-33 years old group:** Durnin/Womersley equation showed **very high/high validity** (derived from 11 studies, r= 0.71-0.95; all, p<0.05)^11, 34, 38, 47, 64, 68, 72, 85, 87, 99, 102^ and **moderate validity** (derived from two studies, r= 0.50-0.62; both, p<0.05)^67, 84^; Jackson, Pollock and Ward equation showed **very high/high validity** (derived from seven studies, r= 0.71-0.92; all, p<0.05)^13, 34, 35, 49, 72, 84, 85^; Jackson/Pollock equation showed **very high/high validity** (derived from four studies, r= 0.77-0.92; all, p<0.05)^12, 34, 49, 52^; BIA equations showed **very high/high validity** (derived from seven studies, r= 0.74-0.96; all, p<0.05)^34, 52, 68, 70, 87, 99, 102^ and **moderate validity** (derived from one study, r= 0.69, p<0.05)^68^; equations implying sum of SKF showed **very high/high validity** (derived from six studies, r= 0.71-0.95; all, p<0.05)^47, 63, 65, 70, 103, 107^; BMI equations showed **very high/high validity** (derived from three studies, r= 0.89-0.92; all, p<0.05)^58, 66, 108^, **moderate validity** (derived from three studies, r= 0.51-0.66; all, p<0.05)^6, 49, 108^ , and **low validity** (derived from one study, r= 0.47, p<0.05)^108^; equations based on WC showed **high validity** (derived from three studies, r= 0.73-0.85; all, p<0.05)^6, 90, 92^ and **moderate validity** (derived from one study, r= 0.64, p<0.05)^6^; equations combining AbC+SKF showed **high validity** (derived from two studies, r= 0.85-0.92; both, p<0.05)^47, 85^; equations combining various circumferences showed **very high/high validity** (derived from two studies, r= 0.90-0.93; both, p<0.05)^59, 64^; equations combining circumferences+diameters+SKF showed **very high validity** (derived from two studies, r= 0.92-0.94; both, p<0.05)^105, 106^; WHtR equations showed **high validity** (derived from two studies, r= 0.72-0.87; both, p<0.05)^90, 92^; equations combining AbC+NC showed **very high validity** (derived from one study, r= 0.90, p<0.05)^59^; equations combining WC+SKF showed **very high/high validity** (derived from one study, r= 0.88-0.99, p<0.05)^111^; equations combining WC+SKF+BMI showed **high validity** (derived from one study, r= 0.82, p<0.05)^71^; equations combining circumferences+SKF showed **high validity** (derived from one study, r= 0.82, p<0.05)^104^; equations combining SKF+BMI showed **high validity** (derived from one study, EST= 0.17-0.19, p<0.005)^50^; BAI equations showed **moderate validity** (derived from one study, r= 0.56-0.64, p<0.05)^6^ and; WHR equations showed **low validity** (derived from one study, r= 0.30, p<0.05)^90^.

**The 34-49 years old group:** Durnin/Womersley equation showed **very high/high validity** (derived from 11 studies, r= 0.75-0.95; all, p<0.05)^11, 36, 37, 47, 62, 68, 72, 76, 79, 85, 100^, **moderate validity** (derived from one study, r= 0.63, p<0.01)^100^ and **very low/low validity** (derived from one study, r= 0.00-0.39, p<0.05)^101^; Jackson, Pollock and Ward equation showed **high validity** (derived from five studies, r= 0.77-0.89; all, p<0.05)^13, 62, 81, 83, 85^; Jackson/Pollock equation showed **very high/high validity** (derived from three studies, r= 0.86-0.92; all, p<0.05)^12, 62, 83^ and **moderate validity** (derived from one study, r= 0.67, p<0.05)^72^; BMI equations showed **very high/high validity** (derived from 11 studies, r= 0.72-0.90; all, p<0.05)^36, 54-56, 58, 61, 74, 83, 88, 89, 108^ and **moderate validity** (derived from three studies, r= 0.66-0.68; all, p<0.05)^36, 54, 55^; equations combining circumferences+SKF showed **very high validity** (derived from seven studies, r= 0.75-0.97; all, p<0.05)^39, 42, 46, 73, 94, 96, 105^; equations implying sum of SKF showed **very high/high validity** (derived from six studies, r= 0.70-0.95; all, p<0.05)^36, 47, 81, 103, 107, 109^; BIA equations showed **very high/high validity** (derived from six studies, r= 0.75-0.98; all, p<0.05)^68, 78, 79, 83, 93, 100^ and **moderate validity** (derived from one study, r= 0.69, p<0.05)^68^; BAI equations showed showed **high validity** (derived from five studies, r= 0.72-0.85; all, p<0.05)^10, 56, 74, 83, 89^ and **low validity** (derived from one study, r= 0.35, p<0.05)^56^; equations based on WC showed **high validity** (derived from four studies, r= 0.74-0.89; all, p<0.05)^36, 39, 41, 83^; equations combining AbC+SKF showed **very high/high validity** (derived from three studies, r= 0.84-0.97; all, p<0.05)^47, 51, 85^; equations combining WC+SKF showed **very high validity** (derived from three studies, r= 0.80-0.93; all, p<0.05)^37, 62, 80^; WHtR equations showed **high validity** (derived from two studies, r= 0.74-0.82; both, p<0.05)^36, 88^ and **moderate validity** (derived from one study, r= 0.47-0.49, p<0.05)^56^; equations combining WC+SKF+BMI showed **very high/high validity** (derived from two studies, r= 0.82-0.91; both, p<0.05)^53, 71^; equations combining various circumferences showed **very high/high validity** (derived from two studies, r= 0.83-0.92; both, p<0.05)^39, 46^; equations combining circumferences+diameters+SKF showed **very high validity** (derived from two studies, r= 0.92-0.95; both, p<0.05)^57, 106^; WHR equations showed **high validity** (derived from one study, r= 0.82-0.84, p<0.05)^56^ and **moderate validity** (derived from one study, r= 0.55-0.65, p<0.05)^83^; equations combining AbC+NC showed **high validity** (derived from one study, r= 0.81-0.88, p<0.05)^98^ and; equations combining SKF+BMI showed **high validity** (derived from one study, r= 0.89, p<0.05)^62^.

**The 50-64 years old group:** Durnin/Womersley equation showed **very high/high validity** (derived from two studies, r= 0.84-0.95; both, p<0.05)^11, 37^ and **low validity** (derived from one study, r= 0.44, p<0.05)^86^; Jackson/Pollock equation showed **very high validity** (derived from one study, r= 0.91-0.92; all, p<0.05)^12^; BMI equations showed **very high/high validity** (derived from four studies, r= 0.71-0.90; all, p<0.05)^45, 54, 58, 108^ and **moderate validity** (derived from two studies, r= 0.50-0.68; both, p<0.05)^54, 108^; equations implying sum of SKF showed **high validity** (derived from two studies, r= 0.80-0.85; both, p<0.05)^107, 109^ and **moderate validity** (derived from one study, r= 0.52, p<0.05)^45^; BIA equations showed **very high/high validity** (derived from two studies, r= 0.80-0.91; both, p<0.001)^44, 77^ and **moderate validity** (derived from one study, r= 0.56, p<0.05)^86^; equations combining WC+SKF+BMI showed **high validity** (derived from two studies, r= 0.82-0.89; both, p<0.05)^44, 71^; equations combining circumferences+SKF showed very **high validity** (derived from two studies, r= 0.95-0.96; both, p<0.05)^48, 96^; equations combining WC+SKF showed **high validity** (derived from one study, r= 0.80-0.89, p<0.05)^37^; equations combining SKF+BMI showed **high validity** (derived from one study, r= 0.82-0.87, p<0.05)^60^ and; equations based on WC showed **moderate validity** (derived from one study, r= 0.56, p<0.05)^45^.

***Weight status groups***

**Group with normal weight**: Durnin/Womersley equation showed **very high/high validity** (derived from nine studies, r= 0.71-0.94; all, p<0.05)^36-38, 68, 76, 79, 85, 87, 99^, **moderate validity** (derived from three studies, r= 0.50-0.62; all, p<0.05)^36, 67, 84^, and **low validity** (derived from one study, r= 0.26, p<0.05)^101^; Jackson, Pollock and Ward equation showed **very high/high validity** (derived from three studies, r= 0.77-0.92; all, p<0.05)^35, 84, 85^; BMI equations showed **high validity** (derived from seven studies, r= 0.75-0.93; all, p<0.05)^53-55, 58, 66, 68, 108^, **moderate validity** (derived from three studies, r= 0.55-0.62; all, p<0.05)^6, 36, 68^, and **low validity** (derived from one study, r= 0.42, p<0.05)^36^; BIA equations showed **very high/high validity** (derived from four studies, r= 0.76-0.96; all, p<0.05)^34, 68, 79, 87^ and **moderate validity** (derived from two studies, r= 0.54-0.69; both, p<0.05)^68, 99^; equations based on WC showed **high validity** (derived from three studies, r= 0.71-0.85; all, p<0.05)^6, 36, 92^, **moderate validity** (derived from one study, r= 0.64, p<0.05)^6^, and **low validity** (derived from one study, r= 0.46, p<0.05)^36^; equations combining WC+SKF showed **very high/high validity** (derived from three studies, r= 0.80-0.93; all, p<0.05)^37, 62, 80^; equations combining circumferences+SKF showed **very high validity** (derived from three studies, r= 0.94-0.97; all, p<0.05)^48, 73, 96^; equations combining WC+SKF+BMI showed **very high/high validity** (derived from two studies, r= 0.82-0.91; both, p<0.05)^53, 71^; equations implying sum of SKF showed **high validity** (derived from two studies, r= 0.77-0.88; both, p<0.05)^34, 36^; equations combining SKF+BMI showed **high validity** (derived from two studies, r= 0.82-0.89; both, p<0.05)^60, 62^; WHtR equations showed **high validity** (derived from two studies, r= 0.71-0.87; both, p<0.05)^36, 92^, and **low validity** (derived from one study, r= 0.46, p<0.05)^36^; equations combining AbC+NC showed **very high validity** (derived from one study, r= 0.90, p<0.05)^59^; equations combining AbC+SKF showed **high validity** (derived from one study, r= 0.85, p<0.05)^85^; equations combining various circumferences showed **very high validity** (derived from one study, r= 0.90, p<0.05)^59^; BAI equations showed **moderate validity** (derived from one study, r= 0.56-0.64, p<0.05)^6^ and; WHR equations showed **very low/low validity** (derived from one study, r= 0.21-0.27, p<0.05)^53^.

**Group with overweight:** Durnin/Womersley equation showed **very high/high validity** (derived from three studies, r= 0.80-0.92; all, p<0.05)^37, 47, 100^, **moderate validity** (derived from three studies, r= 0.59-0.65; all, p<0.05)^36, 99, 100^, and **low validity** (derived from three studies, r= 0.17-0.44; all, p<0.05)^36, 86, 101^; Jackson, Pollock and Ward equation showed **high validity** (derived from two studies, r= 0.82-0.89; both, p<0.05)^81, 83^; Jackson/Pollock equation showed **high validity** (derived from one study, r= 0.89, p<0.05)^83^; BMI equations showed **very high/high validity** (derived from 11 studies, r= 0.71-0.94; all, p<0.05)^45, 54, 55, 58, 61, 66, 74, 83, 88, 89, 108^, **moderate validity** (derived from one study, r= 0.66, p<0.05)^54^, and **low validity** (derived from one study, r= 0.14-0.45, p<0.05)^36^; BIA equations showed **very high/high validity** (derived from six studies, r= 0.75-0.98; all, p<0.05)^44, 78, 83, 93, 99, 100^ and **moderate validity** (derived from one study, r= 0.56, p<0.05)^86^; equations combining circumferences+SKF showed **very high/high validity** (derived from five studies, r= 0.79-0.96; all, p<0.05)^39, 42, 46, 48, 73^; equations based on WC showed **high validity** (derived from three studies, r= 0.81-0.89; all, p<0.05)^39, 41, 83^, **moderate validity** (derived from two studies, r= 0.56-0.62; both, p<0.05)^36, 45^, and **low validity** (derived from one study, r= 0.20, p<0.05)^36^; equations combining WC+SKF showed **very high/high validity** (derived from three studies, r= 0.80-0.99; all, p<0.05)^37, 42, 111^; equations combining various circumferences showed **very high validity** (derived from three studies, r= 0.83-0.92; all, p<0.05)^39, 46, 59^; BAI equations showed **high validity** (derived from three studies, r= 0.72-0.83; all, p<0.05)^74, 83, 89^; equations implying sum of SKF showed **very high/high validity** (derived from two studies, r= 0.82-0.92; both, p<0.05)^47, 81^, **moderate validity** (derived from two studies, r= 0.52-0.65; both, p<0.05)^36, 45^, and **low validity** (derived from 1 studies, r= 0.17, p<0.05)^36^; equations combining AbC+NC showed **very high/high validity** (derived from two studies, r= 0.81-0.90; both, p<0.05)^59, 98^; equations combining AbC+SKF showed **very high/validity** (derived from two studies, r= 0.94-0.97; both, p<0.05)^47, 51^; equations combining SKF+BMI showed **very high/high validity** (derived from two studies, r= 0.81-0.93; both, p<0.05)^50, 60^; WHtR equations showed **high validity** (derived from one study, r= 0.74-0.82, p<0.05)^88^, **moderate validity** (derived from one study, r= 0.62, p<0.05)^36^, and **low validity** (derived from one study, r= 0.20, p<0.05)^36^; equations combining WC+SKF+BMI showed **high validity** (derived from one study, r= 0.89, p<0.05)^44^ and; WHR equations showed **moderate validity** (derived from one study, r= 0.55-0.65, p<0.05)^83^.

**Group with obesity:** Durnin/Womersley equation showed **very high/high validity** (derived from one study, r= 0.85-0.92, p<0.05)^64^; **moderate validity** (derived from one study, r= 0.60, p<0.05)^36^, and **low validity** (derived from for studies, r= 0.00-0.44; all, p<0.05)^36, 86, 99, 101^; BMI equations showed **very high/high validity** (derived from four studies, r= 0.77-0.90; all, p<0.05)^56, 58, 66, 108^, **moderate validity** (derived from one study, r= 0.54, p<0.05)^36^, and **low validity** (derived from one study, r= 0.28, p<0.05)^36^; BIA equations showed **very high/high validity** (derived from three studies, r= 0.80-0.92; all, p<0.05)^44, 77, 99^ and **moderate validity** (derived from one study, r= 0.56, p<0.05)^86^; equations combining circumferences+SKF showed **very high validity** (derived from two studies, r= 0.94-0.97; both, p<0.05)^48, 73^; BAI equations showed **high validity** (derived from two studies, r= 0.81-0.85; both, p<0.05)^10, 56^ and **low validity** (derived from one study, r= 0.35, p<0.05)^56^; equations based on WC showed **moderate validity** (derived from one study, r= 0.64, p<0.05)^36^ and **low validity** (derived from one study, r= 0.35, p<0.05)^36^; equations implying sum of SKF showed **moderate validity** (derived from one study, r= 0.65, p<0.05)^36^ and **low validity** (derived from one study, r= 0.17, p<0.05)^36^; and WHtR equations showed **moderate validity** (derived from one study, r= 0.64, p<0.05)^36^ and **low validity** (derived from two studies, r= 0.35-0.49; both, p<0.05)^36, 56^; equations combining SKF+BMI showed **high validity** (derived from one study, r= 0.82-0.87, p<0.05)^60^; WHR equations showed **high validity** (derived from one study, r= 0.82-0.84, p<0.05)^56^; equations combining WC+SKF+BMI showed **high validity** (derived from one study, r= 0.89, p<0.05)^44^ and; equations combining various circumferences showed **very high validity** (derived from one study, r= 0.90-0.93, p<0.05)^64^.

***Race-ethnicity groups***

**Caucasian/White adults:** Durnin/Womersley equation showed **very high/high validity** (derived from five studies, r= 0.73-0.92; all, p<0.05)^36-38, 47, 64^; Jackson/Pollock equation showed **very high/high validity** (derived from two studies, r= 0.88-0.90; both, p<0.05)^49, 52^; Jackson, Pollock and Ward equation showed **very high/high validity** (derived from two studies, r= 0.88-0.92; both, p<0.05)^35, 49^; BMI equations showed **very high/high validity** (derived from 10 studies, r= 0.71-0.93; all, p<0.05)^36, 45, 53-56, 58, 61, 66, 89^ and **moderate validity** (derived from two studies, r= 0.55-0.64; both, p<0.05)^6, 49^; equations combining circumferences+SKF showed **very high/high validity** (derived from seven studies, r= 0.75-0.96; all, p<0.05)^25, 39, 42, 46, 48, 94, 96^; equations implying sum of SKF showed **very high/high validity** (derived from five studies, r= 0.73-0.95; all, p<0.05)^34, 36, 47, 63, 65^ and **moderate validity** (derived from one study, r= 0.52, p<0.05)^45^; equations based on WC showed **high validity** (derived from four studies, r= 0.72-0.89; all, p<0.05)^6, 36, 39, 41^ and **moderate validity** (derived from two studies, r= 0.56-0.64; both, p<0.05)^6, 45^; equations combining various circumferences showed **very high validity** (derived from four studies, r= 0.83-0.93; all, p<0.05)^39, 46, 59, 64^; equations combining WC+SKF showed **very high/high validity** (derived from three studies, r= 0.80-0.93; all, p<0.05)^37, 42, 62^; equations combining SKF+BMI showed **very high/high validity** (derived from three studies, r= 0.81-0.93; all, p<0.05)^50, 60, 62^; BIA equations showed **very high/high validity** (derived from three studies, r= 0.74-0.92; all, p<0.05)^34, 44, 52^; BAI equations showed **high validity** (derived from two studies, r= 0.72-0.81; both, p<0.05)^56, 89^, **moderate validity** (derived from one study, r= 0.56-0.64, p<0.05)^6^, and **low validity** (derived from one study, r= 0.35, p<0.05)^56^; equations combining AbC+SKF showed **very high validity** (derived from two studies, r= 0.94-0.97; both, p<0.05)^47, 51^; equations combining WC+SKF+BMI showed **very high/high validity** (derived from two studies, r= 0.89-0.90; both, p<0.05)^44, 53^; WHR equations showed **high validity** (derived from one study, r= 0.82-0.84, p<0.05)^56^ and **low validity** (derived from one study, r= 0.21-0.27, p<0.05)^53^; WHtR equations showed **high validity** (derived from one study, r= 0.72-0.82, p<0.05)^36^ and **low validity** (derived from one study, r= 0.47-0.49, p<0.05)^56^; equations combining AbC+NC showed **high validity** (derived from one study, r= 0.88, p<0.05)^59^ and; equations combining circumferences+diameters+SKF showed **very high validity** (derived from one study, r= 0.96-0.99, p<0.05)^57^.

**African-American/Black adults:** Durnin/Womersley equation showed **high validity** (derived from two studies, r= 0.74-0.89; both, p<0.05)^36-38^ and **moderate validity** (derived from one study, r= 0.50-0.53, p<0.05)^67^; Jackson, Pollock and Ward equation showed **very high/high validity** (derived from one study, r= 0.89-0.92, p<0.05)^35^; BMI equations showed **very high/high validity** (derived from five studies, r= 0.72-0.93; all, p<0.05)^36, 54, 55, 61, 66^ and **moderate validity** (derived from one study, r= 0.55-0.61, p<0.05)^6^; equations based on WC showed **high validity** (derived from four studies, r= 0.70-0.89; all, p<0.05)^6, 36, 39, 41^ and **moderate validity** (derived from one study, r= 0.64, p<0.05)^6^; equations combining various circumferences showed **high validity** (derived from two studies, r= 0.83-0.86; both, p<0.05)^39, 59^; equations implying sum of SKF showed **very high/high validity** (derived from two studies, r= 0.74-0.90; both, p<0.05)^36, 65^; BAI equations showed **high validity** (derived from one study, r= 0.85, p<0.05)^10^ and **moderate validity** (derived from one study, r= 0.56-0.64, p<0.05)^6^; equations combining AbC+NC showed **very high validity** (derived from one study, r= 0.90, p<0.05)^59^; equations combining WC+SKF showed **high validity** (derived from one study, r= 0.80-0.89, p<0.05)^37^; equations combining circumferences+SKF showed **high validity** (derived from one study, r= 0.79-0.89, p<0.05)^39^; equations combining circumferences+diameters+SKF showed **high validity** (derived from one study, r= 0.96-0.99, p<0.05)^57^; equations combining SKF+BMI showed **high validity** (derived from one study, EST= 0.17-0.19, p<0.005)^50^ and; WHtR equations showed **high validity** (derived from one study, r= 0.70-0.84, p<0.05)^36^.

**Hispanic adults:** Durnin/Womersley equation showed **high validity** (derived from four studies, r= 0.75-0.89; all, p<0.05)^36-38, 85^ and **moderate validity** (derived from two studies, r= 0.62-0.68; both, p<0.05)^36, 84^; Jackson, Pollock and Ward equation showed **very high/high validity** (derived from four studies, r= 0.71-0.92; all, p<0.05)^35, 83-85^; Jackson/Pollock equation showed **high validity** (derived from one study, r= 0.89, p<0.05)^83^; equations based on WC showed **high validity** (derived from six studies, r= 0.73-0.89; all, p<0.05)^6, 36, 39, 41, 83, 92^ and **moderate validity** (derived from two studies, r= 0.64-0.68; both, p<0.05)^6, 36^; BIA equations showed **very high/high validity** (derived from three studies, r= 0.75-0.98; all, p<0.05)^77, 78, 83^; BAI equations showed **high validity** (derived from two studies, r= 0.72-0.85; both, p<0.05)^10, 83^ and **moderate validity** (derived from one study, r= 0.56-0.64, p<0.05)^6^; BMI equations showed **high validity** (derived from two studies, r= 0.70-0.82; both, p<0.05)^36, 83^ and **moderate validity** (derived from one study, r= 0.55-0.61, p<0.05)^6^; WHtR equations showed **high validity** (derived from two studies, r= 0.81-0.87; both, p<0.05)^36, 92^ and **moderate validity** (derived from one study, r= 0.68, p<0.05)^36^; equations combining WC+SKF showed **very high/high validity** (derived from two studies, r= 0.80-0.99; both, p<0.05)^37, 111^; equations combining various circumferences showed **very high validity** (derived from two studies, r= 0.83-0.92; both, p<0.05)^39, 59^; equations implying sum of SKF showed **high validity** (derived from one study, r= 0.79, p<0.05)^36^ and **moderate validity** (derived from one study, r= 0.68, p<0.05)^36^; equations combining AbC+NC showed **high validity** (derived from one study, r= 0.89, p<0.05)^59^; equations combining AbC+SKF showed **high validity** (derived from one study, r= 0.85, p<0.05)^85^; equations combining circumferences+SKF showed **high validity** (derived from one study, r= 0.79-0.89, p<0.05)^39^; equations combining SKF+BMI showed **high validity** (derived from one study, EST= 0.17-0.19, p<0.005)^50^ and; WHR equations showed **moderate validity** (derived from one study, r= 0.55-0.65, p<0.05)^83^.

**Asian adults:** Durnin/Womersley equation showed **very high/high validity** (derived from six studies, r= 0.70-0.94; all, p<0.05)^37, 68, 72, 76, 79, 87^; Jackson, Pollock and Ward equation showed **very high/high validity** (derived from three studies, r= 0.82-0.93; all, p<0.05)^35, 72, 81^; Jackson/Pollock equation showed **moderate validity** (derived from one study, r= 0.67, p<0.05)^72^; BIA equations showed **very high/high validity** (derived from four studies, r= 0.71-0.96; all, p<0.05)^68, 70, 79, 87^ and **moderate validity** (derived from one study, r= 0.69, p<0.05)^68^; BMI equations showed **very high/high validity** (derived from three studies, r= 0.82-0.94; all, p<0.05)^55, 68, 74^ and **moderate validity** (derived from one study, r= 0.62, p<0.05)^68^; equations combining WC+SKF showed **very high/high validity** (derived from two studies, r= 0.80-0.93; both, p<0.05)^37, 80^; equations implying sum of SKF showed **high validity** (derived from two studies, r= 0.71-0.82; both, p<0.05)^70, 81^; equations based on WC showed **high validity** (derived from one study, r= 0.89, p<0.05)^41^; equations combining WC+SKF+BMI showed **high validity** (derived from one study, r= 0.82, p<0.05)^71^; equations combining circumferences+SKF showed **very high validity** (derived from one study, r= 0.94-0.97, p<0.05)^73^; equations combining SKF+BMI showed **high validity** (derived from one study, r= 0.82-0.85, p<0.05)^60^ and; BAI equations showed **high validity** (derived from one study, r= 0.83, p<0.05)^74^.

**Other different races-ethnicities:** In Australian adults, WHtR and BMI equations showed **high validity** (r= 0.83-0.87, p<0.01)^88^; in Canadian adults, BIA equations showed **high validity** (r= 0.88, p<0.05)^93^; in Polynesian females, Durnin/Womersley equation showed **high validity** (r= 0.85, p<0.05)^64^, and equations combining various circumferences showed **very high validity** (r= 0.90, p<0.05)^64^; in Syrian males, equations based on WC and WHtR showed **very high/high validity** (r= 0.72-0.74, p<0.01)^90^, and WHR **low validity** (r= 0.33, p<0.001)^90^; in Anglo-Celtic Australian adults, BIA equations showed **moderate validity** (r= 0.56, p<0.0001)^86^, and Durnin/Womersley equation showed **low validity** (r= 0.44, p<0.0001)^86^.
