# Supplementary material for: Criterion-Related Validity of Field-Based Methods and Equations for Body Composition Estimation in Adults: A Systematic Review
Source: Curr Obes Rep. 2022 Nov 11;11(4):336–49. doi: 10.1007/s13679-022-00488-8 (PMC9729144; doi:10.1007/s13679-022-00488-8)
Supplement: Supplementary file 4 — Supplementary file4 (DOCX 14 KB) [file 13679_2022_488_MOESM4_ESM.docx]

**Supplementary Table S1**. Quality assessment criteria for validity studies^1^.

| Grading system parameter | Grade | Criterion |
| --- | --- | --- |
| Number of study subjects | 0 | n ≤ 10 |
|  | 1 | n = 11-50 |
|  | 2 | n ≥ 51 |
|  |  |  |
| Description of the study population | 0 | Less items than required for grade 1 |
| with respect to age, sex, health status and body composition, menopausal status, ethnicity, physical activity patterns, fitness levels, etc. | 1 | At least age, sex, health status, and body composition |
|  | 2 | More items than required for grade 1 |
|  |  |  |
| Statistical analysis included in the study | 0 | Those not included in 1 |
|  | 1 | Error indexes or regression analysis |
|  | 2 | ≥ 3 items or Bland-Altman plot and/or ANOVA for repeated measurements |

Rating for total score:

High quality = 5-6

Low quality = 3-4

Very low quality = 0-2

**Reference**

1. Castro-Piñero J, Artero EG, España-Romero V, et al. Criterion-related validity of field-based fitness tests in youth: a systematic review. *British journal of sports medicine*. 2010;44(13):934-943.
