# Supplementary material for: Criterion-Related Validity of Field-Based Methods and Equations for Body Composition Estimation in Adults: A Systematic Review
Source: Curr Obes Rep. 2022 Nov 11;11(4):336–49. doi: 10.1007/s13679-022-00488-8 (PMC9729144; doi:10.1007/s13679-022-00488-8)
Supplement: Supplementary file 5 — Supplementary file5 (DOCX 24 KB) [file 13679_2022_488_MOESM5_ESM.docx]

**Supplementary Table S2.** Quality assessment of validity of field-based body composition estimation in adults.

| Study | Measurement of body composition | Gold Standard | Number of study subjects | Description of the study population | Statistical analysis | Total score |
| --- | --- | --- | --- | --- | --- | --- |
| Sun et al. 2013^1^ | BAI, BMI | DXA | 2 | 2 | 0 | 4 |
| Day et al. 2018^2^ | BIA, WC, WHtR | DXA | 1 | 2 | 2 | 5 |
| Kim et al. 2015^3^ | BMI, WC | DXA | 2 | 2 | 1 | 5 |
| Morabia et al. 1999^4^ | BIA, SKF | DXA | 2 | 2 | 1 | 5 |
| Shaw et al. 2007^5^ | BMI, HC, WC, WHR | DXA | 2 | 1 | 2 | 5 |
| Sun et al. 2010^6^ | BMI, SKF | DXA | 2 | 2 | 1 | 5 |
| Cerqueira et al. 2013^7^ | BAI | DXA | 2 | 2 | 2 | 6 |
| Chang et al. 2014^8^ | BAI, BMI | DXA | 2 | 2 | 2 | 6 |
| McLean et al. 1992^9^ | BMI, NIR, SKF | UWW | 2 | 2 | 2 | 6 |
| Zhang et al. 2014^10^ | BAI, BMI, HC, WC | DXA | 2 | 2 | 2 | 6 |

BAI, body adiposity index; BIA, bioelectrical impedance analysis; BMI, body mass index; DXA, dual-energy x-ray absorptiometry; HC, hip circumference; NIR, near-infrared interactance; SKF, skinfolds; UWW, under water weighing; WC, waist circumference; WHR, waist-hip ratio; WHtR, waist height ratio.

**References**

1. Sun G, Cahill F, Gulliver W, et al. Concordance of BAI and BMI with DXA in the Newfoundland population. *Obesity (Silver Spring)*. Mar 2013;21(3):499-503. doi:10.1002/oby.20009

2. Day K, Kwok A, Evans A, et al. Comparison of a Bioelectrical Impedance Device against the Reference Method Dual Energy X-Ray Absorptiometry and Anthropometry for the Evaluation of Body Composition in Adults. *Nutrients*. Oct 10 2018;10(10)doi:10.3390/nu10101469

3. Kim SG, Ko K, Hwang IC, et al. Relationship between indices of obesity obtained by anthropometry and dual-energy X-ray absorptiometry: The Fourth and Fifth Korea National Health and Nutrition Examination Survey (KNHANES IV and V, 2008-2011). *Obes Res Clin Pract*. Sep-Oct 2015;9(5):487-98. doi:10.1016/j.orcp.2014.11.002

4. Morabia A, Ross A, Curtin F, Pichard C, Slosman DO. Relation of BMI to a dual-energy X-ray absorptiometry measure of fatness. *Br J Nutr*. Jul 1999;82(1):49-55. doi:10.1017/s0007114599001117

5. Shaw KA, Srikanth VK, Fryer JL, Blizzard L, Dwyer T, Venn AJ. Dual energy X-ray absorptiometry body composition and aging in a population-based older cohort. *Int J Obes (Lond)*. Feb 2007;31(2):279-84. doi:10.1038/sj.ijo.0803417

6. Sun Q, van Dam RM, Spiegelman D, Heymsfield SB, Willett WC, Hu FB. Comparison of dual-energy x-ray absorptiometric and anthropometric measures of adiposity in relation to adiposity-related biologic factors. *Am J Epidemiol*. Dec 15 2010;172(12):1442-54. doi:10.1093/aje/kwq306

7. Cerqueira M, Amorim P, Magalhaes F, et al. Validity of body adiposity index in predicting body fat in a sample of brazilian women. *Obesity*. Dec 2013;21(12):E696-E699. doi:10.1002/oby.20543

8. Chang H, Simonsick EM, Ferrucci L, Cooper JA. Validation study of the body adiposity index as a predictor of percent body fat in older individuals: findings from the BLSA. *J Gerontol A Biol Sci Med Sci*. Sep 2014;69(9):1069-75. doi:10.1093/gerona/glt165

9. McLean KP, Skinner JS. Validity of Futrex-5000 for body composition determination. *Med Sci Sports Exerc*. Feb 1992;24(2):253-8.

10. Zhang Z-Q, Liu Y-H, Xu Y, et al. The validity of the body adiposity index in predicting percentage body fat and cardiovascular risk factors among Chinese. *Clinical Endocrinology*. Sep 2014;81(3):356-362. doi:10.1111/cen.12351
