# Supplementary material for: Criterion-Related Validity of Field-Based Methods and Equations for Body Composition Estimation in Adults: A Systematic Review
Source: Curr Obes Rep. 2022 Nov 11;11(4):336–49. doi: 10.1007/s13679-022-00488-8 (PMC9729144; doi:10.1007/s13679-022-00488-8)
Supplement: Supplementary file 6 — Supplementary file6 (DOCX 109 KB) [file 13679_2022_488_MOESM6_ESM.docx]

**Supplementary Table S3.** Quality assessment of validity of estimation equations for body composition in adults.

| Study | Measurement of body composition, equations | Gold Standard | Number of study subjects | Description of the study population | Statistical analysis | Total score |
| --- | --- | --- | --- | --- | --- | --- |
| Hodgdon & Beckett 1984^1^ | AbC, AC, CC, ChC, FC, NC, ShC, SKF, TC, WrC | UWW | 2 | 0 | 1 | 3 |
| Sloan, Burt & Blyth 1962^2^ | AC, ChC, GlC, TC, WC, SKF | UWW | 1 | 1 | 1 | 3 |
| Durnin & Rahaman 1967^3^ | AC, Bi-acromial D, Bi-iliac D, CC, SKF, TC | UWW | 2 | 1 | 1 | 4 |
| Erselcan et al. 2000^4^ | BIA, SKF | DXA | 1 | 1 | 2 | 4 |
| Eston et al. 2005^5^ | BIA, SKF | DXA | 2 | 2 | 0 | 4 |
| Friedl et al. 1997^6^ | AbC, NC | DXA | 2 | 1 | 1 | 4 |
| Haisman 1970^7^ | SKF | UWW | 2 | 1 | 1 | 4 |
| Katch & McArdle 1973^8^ | AbC, AC, AkC, AkD, Bi-acromial D, Bi-iliac D,  Bitrochanteric D, CC, ChC, ChD, ED, FC, GlC, HdC, KC, KD, ShC, SKF, TC, WrC, WrD | UWW | 2 | 1 | 1 | 4 |
| Katch & McArdle 1975^9^ | AC, ChC, HC, NC, SKF, WC | UWW | 2 | 1 | 1 | 4 |
| Temple et al. 2014^10^ | SKF | ADP | 1 | 2 | 1 | 4 |
| Wilmore & Behnke 1969^11^ | AbC, AC, AD, AkC, AkD, Biacromial D, Bideltoid D, Bi-iliac D, Bi-trochanter D, ChC, ChD, Deltoid C, KD, ED, FC, Foot length, HC, HdC, Hand length, KC, NC, ShC, SKF, TC, WrD | UWW | 2 | 1 | 1 | 4 |
| Wilmore & Behnke 1970^12^ | AbC, AC, AD, AkC, AkD, Biacromial D, Bideltoid D, Bi-iliac D, Bi-trochanter D, ChC, ChD, Deltoid C, KD, ED, FC, Foot length, HC, HdC, Hand length, KC, NC, ShC, SKF, TC, WrD | UWW | 2 | 1 | 1 | 4 |
| Aandstad et al. 2014^13^ | BIA, SKF | DXA | 2 | 1 | 2 | 5 |
| Aristizabal et al. 2008^14^ | HC, SKF | UWW | 2 | 1 | 2 | 5 |
| Aristizabal et al. 2018^15^ | AbC, AC, CC, HC, TC, SKF, WC | UWW | 2 | 2 | 1 | 5 |
| Balas-Nakash et al. 2010^16^ | BIA | DXA | 1 | 2 | 2 | 5 |
| Ball et al. 2004^17^ | HC, SKF, WC | DXA | 2 | 1 | 2 | 5 |
| Brozek & Keys 1951^18^ | AbC, ChC, SKF | UWW | 2 | 2 | 1 | 5 |
| Cui et al. 2014^19^ | BMI, SKF, WC, WHtR | DXA | 2 | 2 | 1 | 5 |
| Davidson et al. 2011^20^ | SKF | DXA | 2 | 2 | 1 | 5 |
| Demura et al. 2002^21^ | BIA, SKF | UWW | 1 | 2 | 2 | 5 |
| Dioum et al. 2005^22^ | SKF | ADP | 2 | 1 | 2 | 5 |
| Durnin & Womersley 1974^23^ | AC, CC, SKF, TC | UWW | 2 | 2 | 1 | 5 |
| Friedl et al. 2001^24^ | AC, AbC, CC, ChC, FC, NC, SKF, TC, WC | DXA | 2 | 2 | 1 | 5 |
| Gallagher et al. 1996^25^ | BMI, HC, Tibia/total body length, WC, WHR | DXA, D_2_O, ^3^H_2_O, UWW | 2 | 2 | 1 | 5 |
| Gallagher et al. 2000^26^ | BMI | DXA, D_2_O, ^3^H_2_O, UWW | 2 | 2 | 1 | 5 |
| Goel et al. 2008^27^ | CC, HC, SKF, TC, WC | DXA | 2 | 2 | 1 | 5 |
| Gómez-Ambrosi 2011^28^ | BAI, BMI, CUN-BAE, Rohrer index, WHR, WHtR | ADP | 2 | 2 | 1 | 5 |
| Gómez et al. 2018^29^ | WC, WHtR | DXA | 2 | 1 | 2 | 5 |
| Jackson & Pollock 1978^30^ | FC, SKF, WC | UWW | 2 | 2 | 1 | 5 |
| Jackson, Pollock & Ward 1980^31^ | GlC, SKF | UWW | 2 | 2 | 1 | 5 |
| Johnson et al. 2012^32^ | BAI, BMI | DXA | 2 | 2 | 1 | 5 |
| Ketel et al. 2007^33^ | BMI, HC, SKF, WC, WHR | DXA | 2 | 2 | 1 | 5 |
| Lahav et al. 2018^34^ | AbC, NC, SKF, WC | DXA | 2 | 1 | 2 | 5 |
| Lanham et al. 2001^35^ | BIA, SKF | D_2_O | 1 | 2 | 2 | 5 |
| Lee et al. 2017^36^ | AC, BMI, CC, SKF, TC, WC | DXA | 2 | 2 | 1 | 5 |
| Lukaski et al. 1986^37^ | BIA, SKF | UWW | 2 | 1 | 2 | 5 |
| Noopa et al. 1979^38^ | AC, BMI, GlC, WC, SKF | K^40^, Isotope dilution | 2 | 2 | 1 | 5 |
| Pasco et al. 2012^39^ | BMI, WC | DXA | 2 | 2 | 1 | 5 |
| Pollock et al. 1975^40^ | AbC, AC, AkC, biacromial D,  bi-iliac D, bitrochanter D, CC, ChC, ChD, FC, GlC, KD, ShC, ShD, SKF, TC, WC, WrC, WrD | UWW | 2 | 2 | 1 | 5 |
| Pollock et al. 1976^41^ | AbC, AC, AkC, biacromion D, bideltoid D, bi-ilium D, bitrochanter D, CC, ChC, chest width, FC, GlC, KD,  ShC, SKF, TC, WC, WrC, WrD | UWW | 2 | 2 | 1 | 5 |
| Pongchaiyakul et al. 2005^42^ | BMI, HC, SKF, WC | DXA | 2 | 1 | 2 | 5 |
| Simoes et al. 2016^43^ | AC, HC, SKF, TC, WC | DXA | 2 | 1 | 2 | 5 |
| Smith & Boyce 1977^44^ | AC, AbC, AkC, Biacromial D, Bi-iliac D, Bi-trochanteric D, CC, ChC, ChD, FC, HC, KD, ShC, SKF, TC, WrD | UWW | 2 | 2 | 1 | 5 |
| Steinkamp et al. 1965^45^ | AC, AkC, ChC, Biacromial D, Bi-iliac D, Chest D, SKF, TC, WC, WrC | K^40^, Isotope dilution | 2 | 2 | 1 | 5 |
| Thomas et al. 1998^46^ | BIA, SKF | MRI | 2 | 1 | 2 | 5 |
| Tucker et al. 2001^47^ | AC, CC, ChC, FA, HC, NC, TC, WC | UWW | 2 | 2 | 1 | 5 |
| Vogel et al. 1988^48^ | AbC, AC, AkC, AkD, Biacromial D, Bi-iliac D, Bi-trochanteric D, CC, ChC, ChD, DD, ED, HC, HdC, KD, NC, ShC, TC, WrC, WrD | UWW | 2 | 2 | 1 | 5 |
| Wang et al. 1994^49^ | AC, ChC, IC, SKF, TC, WC, BMI | DXA | 2 | 2 | 1 | 5 |
| Wang et al. 1996^50^ | BIA, BMI, SKF | UWW | 2 | 1 | 2 | 5 |
| Womersley & Durnin 1977^51^ | SKF | UWW | 2 | 2 | 1 | 5 |
| Al-Bachir et al. 2016^52^ | HC, SKF, WC | D_2_O | 2 | 2 | 2 | 6 |
| Al-Gindan et al. 2015^53^ | HC, WC | MRI | 2 | 2 | 2 | 6 |
| Benito et al. 2019^54^ | BIA, SKF | DXA | 2 | 2 | 2 | 6 |
| Bergman et al. 2011^55^ | BAI, HC, WC | DXA | 2 | 2 | 2 | 6 |
| Bhat et al. 2005^56^ | BIA, BMI, SKF | D_2_O | 2 | 2 | 2 | 6 |
| Deurenberg et al. 1991^57^ | BMI | UWW | 2 | 2 | 2 | 6 |
| Eston et al. 1995^58^ | AC, CC, HC, SKF, TC | UWW | 2 | 2 | 2 | 6 |
| Fedewa et al. 2019^59^ | BIA, WC | DXA | 2 | 2 | 2 | 6 |
| Garcia et al. 2005^60^ | Bone breadths, ChC, chest depth, HC, SKF, TC, WC | DXA | 2 | 2 | 2 | 6 |
| Hassager et al. 1986^61^ | SKF | ADP | 2 | 2 | 2 | 6 |
| Hicks et al. 2000^62^ | BIA, SKF | UWW | 2 | 2 | 2 | 6 |
| Jackson et al. 2002^63^ | BMI | UWW | 2 | 2 | 2 | 6 |
| Jackson et al. 2009^64^ | SKF | DXA | 2 | 2 | 2 | 6 |
| Kagawa, Byrne & Hills 2008^65^ | AbC, BMI, HC, WC, WHtR | DXA | 2 | 2 | 2 | 6 |
| Kanellakis et al. 2010^66^ | SKF, WC, WHR | DXA | 2 | 2 | 2 | 6 |
| Kanellakis et al. 2012^67^ | HC, SKF, WC | DXA | 2 | 2 | 2 | 6 |
| Kanellakis et al. 2017^68^ | BMI, CC, HC, FC, NC, SKF, WC | DXA | 2 | 2 | 2 | 6 |
| Kholi et al. 200^69^ | HC, SKF, WC | DXA | 2 | 2 | 2 | 6 |
| Lam et al. 2013^70^ | BAI, BMI, HC, WC | DXA | 2 | 2 | 2 | 6 |
| Leahy et al. 2013^71^ | AbC, AC, CC, ChC, FC, HC, SKF, TC, WC | DXA | 2 | 2 | 2 | 6 |
| Lean, Han & Deurenberg 1996^72^ | AC, Arm span, BMI, HC, leg length, SKF, TC, WC, WHR | UWW | 2 | 2 | 2 | 6 |
| Lee et al. 2021^73^ | BMI, WC | DXA | 2 | 2 | 2 | 6 |
| Macias et al. 2007^74^ | BIA, HC, WC, WHR | ADP | 2 | 2 | 2 | 6 |
| Manios et al. 2012^75^ | BMI, HC, SKF, WC | DXA | 2 | 2 | 2 | 6 |
| Nickerson et al. 2018^76^ | BMI, SKF | UWW, DXA | 2 | 2 | 2 | 6 |
| O’Connor et al. 2010^77^ | SKF | DXA | 2 | 2 | 2 | 6 |
| Pascale et al. 1956^78^ | SKF | UWW | 2 | 2 | 2 | 6 |
| Ramirez-Zea et al. 2006^79^ | AbC, AC, CC, HC, SKF, TC, WC | UWW | 2 | 2 | 2 | 6 |
| Ramos-Jiménez et al. 2018^80^ | SKF, WC | DXA | 2 | 2 | 2 | 6 |
| Rush et al. 1997^81^ | AbC, BMI, HC, SKF, WC, WHR | Isotope dilution | 2 | 2 | 2 | 6 |
| Segheto et al. 2017^82^ | BAI, BIA, BMI, SKF, WC, WHR | DXA | 2 | 2 | 2 | 6 |
| Shafer et al. 2010^83^ | SKF | ADP | 2 | 2 | 2 | 6 |
| Skoufas et al. 2018^84^ | AbC, AC, CC, ChC, FC, HC, NC, SKF, TC, WC | DXA | 2 | 2 | 2 | 6 |
| Slaughter et al. 1988^85^ | SKF | D_2_O, photon absorptiometry, UWW | 2 | 2 | 2 | 6 |
| Stout et al. 1994^86^ | BIA, SKF | UWW | 2 | 1 | 2 | 6 |
| Sun et al. 2005^87^ | BIA, BMI, WHtR | DXA | 2 | 2 | 2 | 6 |
| Wattanapenpaiboon et al. 1998^88^ | BIA, SKF | DXA | 2 | 2 | 2 | 6 |
| Yao et al. 2002^89^ | BMI, SKF | D_2_O | 2 | 2 | 2 | 6 |
| Zanovec et al. 2009^90^ | BMI | DXA | 2 | 2 | 2 | 6 |

AbC, abdominal circumference; AC, arm circumference; AD, arm diameter; ADP, air-displacement plethysmography; AkC, ankle circumference; AkD, ankle diameter; BAI, body adiposity index; BIA, bioelectrical impedance analysis; BMI, body mass index; C, circumference; CC, calf circumference; ChC, chest circumference; ChD, chest diameter; D, diameter; DD, deltoid diameter; DXA, dual energy x-ray absorptiometry; D_2_O, deuterium oxide; ED, elbow diameter; FC, forearm circumference; GlC, gluteal circumference; HC, hip circumference; HdC, head circumference; ^3^H_2_O, tritium space; IC, iliac circumference; KC, knee circumference; KD, knee diameter; K^40^, Potassium-40; MRI, magnetic resonance imaging; NC, neck circumference; ShC, shoulder circumference; SKF, skinfolds; TC, thigh circumference; UWW, under water weighing; WC, waist circumference; WHR, waist to hip ratio; WHtR, waist to height ratio; WrC, wrist circumference; WrD, wrist diameter.

**REFERENCES**

1. Hodgdon JA, Beckett MB. *Prediction of percent body fat for US Navy women from body circumferences and height*. 1984.

2. Sloan A, Burt J, Blyth C. Estimation of body fat in young women. *Journal of applied physiology*. 1962;17(6):967-970.

3. Durnin J, Rahaman MM. The assessment of the amount of fat in the human body from measurements of skinfold thickness. *British journal of Nutrition*. 1967;21(3):681-689.

4. Erselcan T, Candan F, Saruhan S, Ayca T. Comparison of body composition analysis methods in clinical routine. *Annals of Nutrition and Metabolism*. Sep-Dec 2000;44(5-6):243-248. doi:10.1159/000046691

5. Eston RG, Rowlands AV, Charlesworth S, Davies A, Hoppitt T. Prediction of DXA-determined whole body fat from skinfolds: importance of including skinfolds from the thigh and calf in young, healthy men and women. *European Journal of Clinical Nutrition*. May 2005;59(5):695-702. doi:10.1038/sj.ejcn.1602131

6. Friedl KE, Vogel JA. Validity of percent body fat predicted from circumferences: classification of men for weight control regulations. *Mil Med*. Mar 1997;162(3):194-200.

7. Haisman M. The assessment of body fat content in young men from measurements of body density and skinfold thickness. *Human biology*. 1970:679-688.

8. Katch FI, McArdle WD. Prediction of body density from simple anthropometric measurements in college-age men and women. *Human biology*. 1973:445-455.

9. Katch FI, McArdle WD. VALIDITY OF BODY COMPOSITION PREDICTION EQUATIONS FOR COLLEGE MEN AND WOMEN. *American Journal of Clinical Nutrition*. 1975 1975;28(2):105-109.

10. Temple D, Denis R, Walsh MC, Dicker P, Byrne AT. Comparison of anthropometric-based equations for estimation of body fat percentage in a normal-weight and overweight female cohort: validation via air-displacement plethysmography. *Public Health Nutr*. Feb 2015;18(3):446-52. doi:10.1017/s1368980014000597

11. Wilmore JH, Behnke AR. An anthropometric estimation of body density and lean body weight in young men. *Journal of Applied Physiology*. 1969;27(1):25-31.

12. Wilmore JH, Behnke AR. An anthropometric estimation of body density and lean body weight in young women. *The American journal of clinical nutrition*. 1970;23(3):267-274.

13. Aandstad A, Holtberget K, Hageberg R, Holme I, Anderssen SA. Validity and reliability of bioelectrical impedance analysis and skinfold thickness in predicting body fat in military personnel. *Mil Med*. Feb 2014;179(2):208-17. doi:10.7205/milmed-d-12-00545

14. Aristizabal JC, Restrepo MT, Amalia L. Validation by hydrodensitometry of skinfold thickness equations used for female body composition assessment. *Biomedica*. Sep 2008;28(3):404-13. Validacion por hidrodensitometria de ecuaciones de pliegues cutaneos utilizadas para estimar la composicion corporal en mujeres.

15. Aristizabal JC, Estrada-Restrepo A, García AG. Desarrollo y validación de ecuaciones antropométricas para estimar la composición corporal en mujeres adultas. *Revista Colombia Médica*. 2018;49(2):154-159.

16. Balas-Nakash M, Legorreta-Legorreta J, Rodriguez-Cano A, Aguilera-Perez R, Perichart-Perera O. Validation of body composition estimation equations by bioelectric impedance in postmenopausic women with metabolic syndrome. *Rev Invest Clin*. Nov-Dec 2010;62(6):538-45. Validacion del uso de ecuaciones para estimar la composicion corporal por analisis de impedancia bioelectrica en mujeres postmenopausicas con sindrome metabolico.

17. Ball S, Swan PD, DeSimone R. Comparison of anthropometry to dual energy X-ray absorptiometry: a new prediction equation for women. *Res Q Exerc Sport*. Sep 2004;75(3):248-58. doi:10.1080/02701367.2004.10609158

18. Brožek J, Keys A. The evaluation of leanness-fatness in man: norms and interrelationships. *British Journal of Nutrition*. 1951;5(2):194-206.

19. Cui Z, Truesdale KP, Cai J, Stevens J. Evaluation of anthropometric equations to assess body fat in adults: NHANES 1999-2004. *Med Sci Sports Exerc*. Jun 2014;46(6):1147-58. doi:10.1249/mss.0000000000000213

20. Davidson LE, Wang J, Thornton JC, et al. Predicting Fat Percent by Skinfolds in Racial Groups: Durnin and Womersley Revisited. *Medicine and Science in Sports and Exercise*. Mar 2011;43(3):542-549. doi:10.1249/MSS.0b013e3181ef3f07

21. Demura S, Yamaji S, Goshi F, Kobayashi H, Sato S, Nagasawa Y. The validity and reliability of relative body fat estimates and the construction of new prediction equations for young Japanese adult males. *J Sports Sci*. Feb 2002;20(2):153-64. doi:10.1080/026404102317200864

22. Dioum A, Gartner A, Maire B, Delpeuch F, Wade S. Body composition predicted from skinfolds in African women: a cross-validation study using air-displacement plethysmography and a black-specific equation. *Br J Nutr*. Jun 2005;93(6):973-9. doi:10.1079/bjn20051426

23. Durnin JV, Womersley J. Body fat assessed from total body density and its estimation from skinfold thickness: measurements on 481 men and women aged from 16 to 72 years. *British journal of nutrition*. 1974;32(1):77-97.

24. Friedl KE, Westphal KA, Marchitelli LJ, Patton JF, Chumlea WC, Guo SS. Evaluation of anthropometric equations to assess body-composition changes in young women. *American Journal of Clinical Nutrition*. Feb 2001;73(2):268-275. doi:doi.org/10.1093/ajcn/73.2.268

25. Gallagher D, Visser M, Sepulveda D, Pierson RN, Harris T, Heymsfield SB. How useful is body mass index for comparison of body fatness across age, sex, and ethnic groups? *American journal of epidemiology*. 1996;143(3):228-239.

26. Gallagher D, Heymsfield SB, Heo M, Jebb SA, Murgatroyd PR, Sakamoto Y. Healthy percentage body fat ranges: an approach for developing guidelines based on body mass index. *The American journal of clinical nutrition*. 2000;72(3):694-701.

27. Goel K, Gupta N, Misra A, et al. Predictive equations for body fat and abdominal fat with DXA and MRI as reference in Asian Indians. *Obesity (Silver Spring)*. Feb 2008;16(2):451-6. doi:10.1038/oby.2007.55

28. Gómez-Ambrosi J, Silva C, Catalán V, et al. Clinical usefulness of a new equation for estimating body fat. *Diabetes Care*. 2012;35(2):383-388.

29. Gomez Campos R, Pacheco Carrillo J, Almonacid Fierro A, Urra Albornoz C, Cossio-Bolanos M. Validation of equations and proposed reference values to estimate fat mass in Chilean university students. *Endocrinol Diabetes Nutr*. Mar 2018;65(3):156-163. Validacion de ecuaciones y propuesta de valores referenciales para estimar la masa grasa de jovenes universitarios chilenos. doi:10.1016/j.endinu.2017.11.008

30. Jackson AS, Pollock ML. Generalized equations for predicting body density of men. *British journal of nutrition*. 1978;40(3):497-504.

31. Jackson AS, Pollock ML, Ward A. Generalized equations for predicting body density of women. *Medicine and science in sports and exercise*. 1980;12(3):175-181.

32. Johnson W, Chumlea WC, Czerwinski SA, Demerath EW. Concordance of the recently published body adiposity index with measured body fat percent in European-American adults. *Obesity (Silver Spring)*. Apr 2012;20(4):900-3. doi:10.1038/oby.2011.346

33. Ketel IJ, Volman MN, Seidell JC, Stehouwer CD, Twisk JW, Lambalk CB. Superiority of skinfold measurements and waist over waist-to-hip ratio for determination of body fat distribution in a population-based cohort of Caucasian Dutch adults. *Eur J Endocrinol*. Jun 2007;156(6):655-61. doi:10.1530/eje-06-0730

34. Lahav Y, Epstein Y, Kedem R, Schermann H. A novel body circumferences-based estimation of percentage body fat. *Br J Nutr*. Mar 2018;119(6):720-725. doi:10.1017/s0007114518000223

35. Lanham DA, Stead MA, Tsang K, Davies PSW. The prediction of body composition in Chinese Australian females. *International Journal of Obesity*. Feb 2001;25(2):286-291. doi:10.1038/sj.ijo.0801473

36. Lee DH, Keum N, Hu FB, et al. Development and validation of anthropometric prediction equations for lean body mass, fat mass and percent fat in adults using the National Health and Nutrition Examination Survey (NHANES) 1999-2006. *Br J Nutr*. Nov 2017;118(10):858-866. doi:10.1017/s0007114517002665

37. Lukaski HC, Bolonchuk WW, Hall CB, Siders WA. Validation of tetrapolar bioelectrical impedance method to assess human body composition. *Journal of applied physiology*. 1986;60(4):1327-1332.

38. Noppa H, Andersson M, Bengtsson C, Bruce Å, Isaksson B. Body composition in middle-aged women with special reference to the correlation between body fat mass and anthropometric data. *The American journal of clinical nutrition*. 1979;32(7):1388-1395.

39. Pasco JA, Nicholson GC, Brennan SL, Kotowicz MA. Prevalence of obesity and the relationship between the body mass index and body fat: cross-sectional, population-based data. *PloS one*. 2012;7(1):e29580.

40. Pollock ML, Laughridge EE, Coleman B, Linnerud A, Jackson A. Prediction of body density in young and middle-aged women. *Journal of applied physiology*. 1975;38(4):745-749.

41. Pollock ML, Hickman T, Kendrick Z, Jackson A, Linnerud A, Dawson G. Prediction of body density in young and middle-aged men. *Journal of Applied Physiology*. 1976;40(3):300-304.

42. Pongchaiyakul C, Kosulwat V, Rojroongwasinkul N, et al. Prediction of percentage body fat in rural thai population using simple anthropometric measurements. *Obes Res*. Apr 2005;13(4):729-38. doi:10.1038/oby.2005.82

43. Simoes M, Severo M, Oliveira A, Ferreira I, Lopes C. Predictive equations for estimating regional body composition: a validation study using DXA as criterion and associations with cardiometabolic risk factors. *Ann Hum Biol*. May 2016;43(3):219-28. doi:10.3109/03014460.2015.1054427

44. Smith DP, Boyce RW. Prediction of body density and lean body weight in females 25 to 37 years old. *The American Journal of Clinical Nutrition*. 1977;30(4):560-564.

45. Steinkamp R, Cohen N, Gaffey W, et al. Measures of body fat and related factors in normal adults—II: A simple clinical method to estimate body fat and lean body mass. *Journal of chronic diseases*. 1965;18(12):1291-1307.

46. Thomas EL, Saeed N, Hajnal JV, et al. Magnetic resonance imaging of total body fat. *Journal of Applied Physiology*. Nov 1998;85(5):1778-1785.

47. Tucker LA, Greenwell SD. Using simple measures to estimate body fat percentage in college men. *American Journal of Health Behavior*. Sep-Oct 2001;25(5):460-467. doi:10.5993/ajhb.25.5.3

48. Vogel JA, Kirkpatrick J, Fitzgerald P, Hodgdon JA, Harman E. *Derivation of anthropometry based body fat equations for the Army's weight control program*. 1988.

49. Wang J, Thornton JC, Russell M, Burastero S, Heymsfield S, Pierson Jr RN. Asians have lower body mass index (BMI) but higher percent body fat than do whites: comparisons of anthropometric measurements. *The American journal of clinical nutrition*. 1994;60(1):23-28.

50. Wang J, Deurenberg P. The validity of predicted body composition in Chinese adults from anthropometry and bioelectrical impedance in comparison with densitometry. *Br J Nutr*. Aug 1996;76(2):175-82. doi:10.1079/bjn19960023

51. Womersley J, Durnin J. A comparison of the skinfold method with extent of ‘overweight’and various weight-height relationships in the assessment of obesity. *British journal of nutrition*. 1977;38(2):271-284.

52. Al-Bachir M, Ahmad H. VALIDITY OF USING WAIST AND HIP CIRCUMFERENCE MEASUREMENTS TO DETERMINE BODY COMPOSITION OF YOUNG SYRIAN MEN. *J Biosoc Sci*. Sep 2016;48(5):647-57. doi:10.1017/s0021932015000413

53. Al-Gindan YY, Hankey CR, Govan L, Gallagher D, Heymsfield SB, Lean MEJ. Derivation and validation of simple anthropometric equations to predict adipose tissue mass and total fat mass with MRI as the reference method. *British Journal of Nutrition*. Dec 14 2015;114(11):1852-1867. doi:10.1017/s0007114515003670

54. Benito PJ, Gomez-Candela C, Dolores Cabanas M, Szendrei B, Aparecida Castro E, Grp PS. COMPARISON BETWEEN DIFFERENT METHODS FOR MEASURING BODY FAT AFTER A WEIGHT LOSS PROGRAM. *Revista Brasileira De Medicina Do Esporte*. Nov-Dec 2019;25(6):474-479. doi:10.1590/1517-869220192506149743

55. Bergman RN, Stefanovski D, Buchanan TA, et al. A better index of body adiposity. *Obesity (Silver Spring)*. May 2011;19(5):1083-9. doi:10.1038/oby.2011.38

56. Bhat DS, Yajnik CS, Sayyad MG, et al. Body fat measurement in Indian men: comparison of three methods based on a two-compartment model. *Int J Obes (Lond)*. Jul 2005;29(7):842-8. doi:10.1038/sj.ijo.0802953

57. Deurenberg P, Weststrate JA, Seidell JC. Body mass index as a measure of body fatness: age-and sex-specific prediction formulas. *British journal of nutrition*. 1991;65(2):105-114.

58. Eston RG, Fu F, Fung L. VALIDITY OF CONVENTIONAL ANTHROPOMETRIC TECHNIQUES FOR PREDICTING BODY-COMPOSITION IN HEALTHY CHINESE ADULTS. *British Journal of Sports Medicine*. Mar 1995;29(1):52-56. doi:10.1136/bjsm.29.1.52

59. Fedewa MV, Nickerson BS, Esco MR. Associations of body adiposity index, waist circumference, and body mass index in young adults. *Clinical Nutrition*. Apr 2019;38(2):715-720. doi:10.1016/j.clnu.2018.03.014

60. Garcia AL, Wagner K, Hothorn T, Koebnick C, Zunft HJ, Trippo U. Improved prediction of body fat by measuring skinfold thickness, circumferences, and bone breadths. *Obes Res*. Mar 2005;13(3):626-34. doi:10.1038/oby.2005.67

61. Hassager C, Gotfredsen A, Jensen J, Christiansen C. Prediction of body composition by age, height, weight, and skinfold thickness in normal adults. *Metabolism*. 1986;35(12):1081-1084.

62. Hicks VL, Stolarczyk LM, Heyward VH, Baumgartner RN. Validation of near-infrared interactance and skinfold methods for estimating body composition of American Indian women. *Med Sci Sports Exerc*. Feb 2000;32(2):531-9. doi:10.1097/00005768-200002000-00041

63. Jackson AS, Stanforth PR, Gagnon J, et al. The effect of sex, age and race on estimating percentage body fat from body mass index: The Heritage Family Study. *International journal of obesity*. 2002;26(6):789-796.

64. Jackson AS, Ellis KJ, McFarlin BK, Sailors MH, Bray MS. Cross-validation of generalised body composition equations with diverse young men and women: the Training Intervention and Genetics of Exercise Response (TIGER) Study. *Br J Nutr*. Mar 2009;101(6):871-8. doi:10.1017/s0007114508047764

65. Kagawa M, Byrne NM, Hills AP. Comparison of body fat estimation using waist: height ratio using different ‘waist’measurements in Australian adults. *British Journal of Nutrition*. 2008;100(5):1135-1141.

66. Kanellakis S, Kourlaba G, Moschonis G, Vandorou A, Manios Y. Development and validation of two equations estimating body composition for overweight and obese postmenopausal women. *Maturitas*. Jan 2010;65(1):64-8. doi:10.1016/j.maturitas.2009.10.012

67. Kanellakis S, Manios Y. Validation of five simple models estimating body fat in white postmenopausal women: use in clinical practice and research. *Obesity (Silver Spring)*. Jun 2012;20(6):1329-32. doi:10.1038/oby.2011.403

68. Kanellakis S, Skoufas E, Khudokonenko V, et al. Development and Validation of Two Equations Based on Anthropometry, Estimating Body Fat for the Greek Adult Population. *Obesity*. Feb 2017;25(2):408-416. doi:10.1002/oby.21736

69. Kohli S, Gao M, Lear SA. Using simple anthropometric measures to predict body fat in South Asians. *Appl Physiol Nutr Metab*. Feb 2009;34(1):40-8. doi:10.1139/h08-128

70. Lam BC, Lim SC, Wong MT, et al. A method comparison study to validate a novel parameter of obesity, the body adiposity index, in Chinese subjects. *Obesity (Silver Spring)*. Dec 2013;21(12):E634-9. doi:10.1002/oby.20504

71. Leahy S, O'Neill C, Sohun R, Toomey C, Jakeman P. Generalised equations for the prediction of percentage body fat by anthropometry in adult men and women aged 18-81 years. *Br J Nutr*. Feb 28 2013;109(4):678-85. doi:10.1017/s0007114512001870

72. Lean M, Han TS, Deurenberg P. Predicting body composition by densitometry from simple anthropometric measurements. *The American journal of clinical nutrition*. 1996;63(1):4-14.

73. Lee G, Chang J, Hwang SS, Son JS, Park SM. Development and validation of prediction equations for the assessment of muscle or fat mass using anthropometric measurements, serum creatinine level, and lifestyle factors among Korean adults. *Nutrition Research and Practice*. Feb 2021;15(1):95-105. doi:10.4162/nrp.2021.151.95

74. Macias N, Alemán-Mateo H, Esparza-Romero J, Valencia ME. Body fat measurement by bioelectrical impedance and air displacement plethysmography: a cross-validation study to design bioelectrical impedance equations in Mexican adults. *Nutrition Journal*. 2007;6(1):1-7.

75. Manios Y, Kanellakis S, Androutsos O, et al. Development and validation of a simple model based on anthropometry: estimating fat mass for white postmenopausal women. *Menopause*. Apr 2012;19(4):467-70. doi:10.1097/gme.0b013e31823110db

76. Nickerson BS, Esco MR, Bishop PA, et al. Validity of BMI-Based Body Fat Equations in Men and Women: A 4-Compartment Model Comparison. *J Strength Cond Res*. Jan 2016;32(1):121-129. doi:10.1519/jsc.0000000000001774

77. O'Connor DP, Bray MS, McFarlin BK, Sailors MH, Ellis KJ, Jackson AS. Generalized Equations for Estimating DXA Percent Fat of Diverse Young Women and Men: The TIGER Study. *Medicine and Science in Sports and Exercise*. Oct 2010;42(10):1959-1965. doi:10.1249/MSS.0b013e3181dc2e71

78. Pascale LR, Grossman MI, Sloane HS, Frankel T. Correlations between thickness of skinfolds and body density in 88 soldiers. *Human Biology*. 1956;28(2):165.

79. Ramirez-Zea M, Torun B, Martorell R, Stein AD. Anthropometric predictors of body fat as measured by hydrostatic weighing in Guatemalan adults. *The American journal of clinical nutrition*. 2006;83(4):795-802.

80. Ramos-Jimenez A, Hernandez-Torres RP, Murguia-Romero M. Anthropometric equations for calculating body fat in young adults. *Archivos Latinoamericanos De Nutricion*. Jun 2018;68(2):111-121.

81. Rush EC, Plank LD, Laulu MS, Robinson SM. Prediction of percentage body fat from anthropometric measurements: comparison of New Zealand European and Polynesian young women. *The American journal of clinical nutrition*. 1997;66(1):2-7.

82. Segheto W, Coelho FA, Guimaraes da Silva DC, et al. Validity of body adiposity index in predicting body fat in Brazilians adults. *American Journal of Human Biology*. Jan-Feb 2017;29(1)e22901. doi:10.1002/ajhb.22901

83. Shafer KJ, Siders WA, Johnson LK, Lukaski HC. Body density estimates from upper-body skinfold thicknesses compared to air-displacement plethysmography. *Clin Nutr*. Apr 2010;29(2):249-54. doi:10.1016/j.clnu.2009.09.002

84. Skoufas E, Kanellakis S, Apostolidou E, et al. Development and validation of two anthropometric models estimating abdominal fat percentage in Greek adult women and men. *Clin Nutr ESPEN*. Dec 2018;28:239-242. doi:10.1016/j.clnesp.2018.07.010

85. Slaughter MH, Lohman TG, Boileau R, et al. Skinfold equations for estimation of body fatness in children and youth. *Human biology*. 1988:709-723.

86. Stout JR, Eckerson JM, Housh TJ, Johnson GO, Betts NM. Validity of percent body fat estimations in males. *Med Sci Sports Exerc*. May 1994;26(5):632-6.

87. Sun G, French CR, Martin GR, et al. Comparison of multifrequency bioelectrical impedance analysis with dual-energy X-ray absorptiometry for assessment of percentage body fat in a large, healthy population. *The American journal of clinical nutrition*. 2005;81(1):74-78.

88. Wattanapenpaiboon N, Lukito W, Strauss BJG, Hsu-Hage BH, Wahlqvist ML, Stroud DB. Agreement of skinfold measurement and bioelectrical impedance analysis (BIA) methods with dual energy X-ray absorptiometry (DEXA) in estimating total body fat in Anglo-Celtic Australians. *International Journal of Obesity*. Sep 1998;22(9):854-860. doi:10.1038/sj.ijo.0800672

89. Yao M, Roberts SB, Ma G, Pan H, McCrory MA. Field methods for body composition assessment are valid in healthy chinese adults. *J Nutr*. Feb 2002;132(2):310-7. doi:10.1093/jn/132.2.310

90. Zanovec M, Johnson L, Marx B, Keenan M, Tuuri G. Self-reported physical activity improves prediction of body fatness in young adults. *Medicine+ Science in Sports+ Exercise*. 2009;41(2):328.
