# Supplementary material for: Criterion-Related Validity of Field-Based Methods and Equations for Body Composition Estimation in Adults: A Systematic Review
Source: Curr Obes Rep. 2022 Nov 11;11(4):336–49. doi: 10.1007/s13679-022-00488-8 (PMC9729144; doi:10.1007/s13679-022-00488-8)
Supplement: Supplementary file 7 — Supplementary file7 (DOCX 72 KB) [file 13679_2022_488_MOESM7_ESM.docx]

**Supplementary Table S4.** Overview of field-based body composition estimation studies in adults.

| Author | Participants | Age, range/ SD | Field test | Gold standard | Statistical methods | Results | Conclusions |
| --- | --- | --- | --- | --- | --- | --- | --- |
| ***Low quality studies*** | | | | | | | |
| Sun et al. 2013^1^ | Females=1939  Males=662  Healthy  BMI=26.5 (entire sample) normal weight, overweight and obesity  Caucasian | (≥20)  39.5±14.4  43.3±12.4 | - BAI  - BMI | DXA | Pearson correlation coefficient (r) | Field test correlations vs DXA:  Group with normal weight:  - BAI: r= 0.65 (p<0.001)  - BMI: r= 0.05 (p≥0.05)  Group with overweight:  - BAI: r= 0.62 (p<0.001)  - BMI: r= -0.09 (p<0.001)  Group with obesity:  - BAI: r= 0.68 (p<0.001)  - BMI: r= 0.34 (p<0.001) | The BAI method showed moderate validity to assess body adiposity, compared with DXA.  The BMI showed very low validity to assess body adiposity, in groups with normal weight and overweight, while in the group with obesity showed low validity, compared with DXA. |
| ***High quality studies*** | | | | | | | |
| Day et al. 2018^2^ | Females=16  Males=14  Healthy  BMI=29.2 (entire sample) normal weight, overweight/ obesity  Caucasian | (18-65)  29.9±11.2 | - WC, WHtR  - BIA | DXA | Spearman’s Rank correlations, Bland-Altman method | Field test correlations vs DXA:  Group with normal weight:  - WC: r_s_= 0.57 (p<0.001)  - WHtR: r_s_= 0.59 (p<0.001)  - BIA: r_s_= 0.59 (p<0.001)  Group with overweight/obesity:  - WC: r_s_= 0.84 (p<0.001)  - WHtR: r_s_= 0.75 (p<0.001)  - BIA: r_s_= 0.99 (p<0.001)  Agreement:  - BIA vs DXA, MD (± 95% LoA)= 0.32 (-3.8 to 4.4) | In the group with normal weight, WC, WHtR and BIA showed moderate validity to assess body adiposity, compared with DXA.  In the group with overweight/obesity, WC and BIA showed very high validity and WHtR high validity to assess body adiposity, compared with DXA. |
| Kim et al. 2015^3^ | Females=10.320  Males=7.878  Healthy  BMI=23.6  Korean | (19-59)  44.8±0.2 | - WC  - BMI | DXA | Pearson correlation coefficient (r), linear regression | Field test correlations vs DXA:  - WC: r= 0.23 (p<0.05)  - BMI: r= 0.39 (p<0.05)  Regression analysis:  - WC: R^2^= 0.15 (p<0.05)  - BMI: R^2^= 0.05 (p<0.05) | WC and BMI showed very low and low validity to assess body adiposity, compared with DXA. |
| Morabia et al. 1999^4^ | Females=153  Males=73  Healthy  BMI=22.0  Caucasian | (15-86)  63.5±7.5  62.1±7.3 | - BMI | DXA | Pearson correlation coefficient (r), multiple regression | Field test correlations vs DXA:  - Females: r= 0.81 (p<0.05)  - Males: r= 0.84 (p<0.05)  Regression analysis:  - Females: R^2^= 0.41 (p<0.05)  - Males: R^2^= 0.44 (p<0.05) | BMI showed high validity to assess body adiposity, compared with DXA. |
| Shaw et al. 2007^5^ | Females=307  Males=268  Healthy  2 age-groups: 50-59yrs and 60-69yrs  BMI=28.0  Tasmanian | (50-69) | - HC, WC, WHR  - BMI | DXA | Pearson correlation coefficient (r), Bland-Altman method | Field test correlations vs DXA:  Females:  - HC: r= 0.81 (p<0.0001), in 50-59yrs group; r= 0.77 (p<0.0001), in 60-69yrs group  - WC: r= 0.89 (p<0.0001), in 50-59yrs group; r= 0.87 (p<0.0001), in 60-69yrs group  - WHR: r= 0.51 (p<0.0001), in 50-59yrs group; r= 0.46 (p<0.0001), in 60-69yrs group  - BMI: r= 0.95 (p<0.0001), in 50-59yrs group; r= 0.93 (p<0.0001), in 60-69yrs group  Males:  - HC: r= 0.84 (p<0.0001), in 50-59yrs group; r= 0.85 (p<0.0001), in 60-69yrs group  - WC: r= 0.90 (p<0.0001), in 50-59yrs group; r= 0.90 (p<0.0001), in 60-69yrs group  - WHR: r= 0.63 (p<0.0001), in 50-59yrs group; r= 0.63 (p<0.0001), in 60-69yrs group  - BMI: r= 0.89 (p<0.0001), in 50-59yrs group; r= 0.90 (p<0.0001), in 60-69yrs group  Agreement of body composition measures vs DXA:  **Mean difference was <0.0001 for all comparisons; all comparisons demonstrated no tendency for agreement between measures to vary with the magnitude of measurement.* | HC, WC and BMI showed high to very high validity to assess body adiposity, compared with DXA.  WHR showed low to moderate validity to assess body adiposity, compared with DXA. |
| Sun et al. 2010^6^ | Females=2.126  Males=2.260  Healthy  3 age-groups: 20-39yrs, 40-59yrs, ≥60yrs  *BMI not described*  Non-Hispanic white (NHW), non-Hispanic Black (NHB), Hispanic (H) | (≥20)  42.3±0.3  41.0±0.3 | - WC  - BMI | DXA | Pearson correlation coefficient (r) | Field test correlations vs DXA:  Females (entire sample):  - WC, trunk fat mass (%): r= 0.83 (p<0.05), in 20-39yrs group; r= 0.80 (p<0.05), in 40-59yrs group; r= 0.76 (p<0.05), in ≥60yrs group  - BMI, body adiposity: r= 0.84 (p<0.05), in 20-39yrs group; r= 0.79 (p<0.05), in 40-59yrs group; r= 0.78 (p<0.05), in ≥60yrs group  Males (entire sample):  - WC, trunk fat mass (%): r= 0.86 (p<0.05), in 20-39yrs group; r= 0.72 (p<0.05), in 40-59yrs group; r= 0.77 (p<0.05), in ≥60yrs group  - BMI, body adiposity: r= 0.79 (p<0.05), in 20-39yrs group; r= 0.75 (p<0.05), in 40-59yrs group; r= 0.74 (p<0.05), in ≥60yrs group  Ethnicity (not sex/age distinction):  NHW:  - WC, trunk fat mass (%): r= 0.83 (p<0.05)  - BMI, body adiposity: r= 0.78 (p<0.05)  NHB:  - WC, trunk fat mass (%): r= 0.84 (p<0.05)  - BMI, body adiposity: r= 0.78 (p<0.05)  H:  - WC, trunk fat mass (%): r= 0.78 (p<0.05)  - BMI, body adiposity: r= 0.74 (p<0.05) | WC and BMI showed high validity to assess body adiposity (and trunk fat mass), compared with DXA, independently of sex, age or race-ethnicity. |
| Cerqueira et al. 2013^7^ | Females=102  Healthy  BMI=26.9  Brazilian | (35-83)  60.3±9.8 | - BAI | DXA | Pearson correlation coefficient (r), LCCC (Pc), Bland-Altman method | Field test correlations vs DXA:  - BAI: r= 0.65 (p<0.001)  Concordance:  - Pc= 0.73  Agreement: MD (± 95% LoA)= 3.3 (-5.9 to 12.5) | BAI showed moderate validity to assess body adiposity, compared with DXA. |
| Chang et al. 2014^8^ | Females=471  Males=483  Healthy  BMI=27.2  White  Black | (≥55)  70.4±9.5 | - BAI  - BMI | DXA | Pearson correlation coefficient (r), LCCC (Pc), Linear regression, Bland-Altman method | Field test correlations vs DXA:  Females:  - BAI: r= 0.72 (p<0.01)  - BMI: r= 0.80 (p<0.01)  Males:  - BAI: r= 0.55 (p<0.01)  - BMI: r= 0.71 (p<0.01)  White (not sex distinction):  - BAI: r= 0.70 (p<0.01)  - BMI: r= 0.54 (p<0.01)  Black (not sex distinction):  - BAI: r= 0.81 (p<0.01)  - BMI: r= 0.62 (p<0.01)  Regression analysis:  - BAI: R^2^= 0.56 (p<0.01)  - BMI: R^2^= 0.55 (p<0.01)  Concordance:  - BAI: Pc= 0.55 (p<0.01)  - BMI: Pc= 0.30 (p<0.01)  Agreement:  - BAI vs DXA, MD (± 95% LoA)= -5.17 (-16.94 to 6.59)  - BMI vs DXA, MD (± 95% LoA)= -7.6 (-21.78 to 6.58) | BAI and BMI showed high validity to assess body adiposity, compared with DXA, in females and White and Black populations. In males, BAI showed moderate validity.  However, BAI was not accurate in people with extremely low or high body fat percentages. |
| Mclean et al. 1992^9^ | Females=31  Males=30  Healthy  BMI=24.3  Caucasian | (18-45)  32±8 | - SKF: abdominal, subscapular, suprailiac, thigh, triceps  - NIR  - % BF (from BD by Brozek) | UWW | Pearson correlation coefficient (r), SEE, ANOVA | Field test correlations vs UWW:  Females:  - SKF: r= 0.88, SEE= 2.9 (p<0.001)  - NIR: r= 0.63, SEE= 4.7 (p<0.001)  Males:  - SKF: r= 0.95, SEE= 2.6 (p<0.001)  - NIR: r= 0.80, SEE= 4.9 (p<0.001)  ANOVA:  Females:  - SKF: *F*= 33.4 (p<0.001)  - NIR: *F*= 7.6 (p=0.025)  Males:  - SKF: *F*= 69.8 (p<0.001)  - NIR: *F*= 53.4 (p<0.001) | SKF showed very high validity in the total group to assess body adiposity, compared with UWW. |
| Zhang et al. 2013^10^ | Females=1707  Males=680  BMI=23.6  Chinese | (51-77)  59.6±4.7  62.2±5.2 | - HC, WC  - BAI  - BMI | DXA | Pearson correlation coefficient (r), Bland-Altman method | Field test correlations vs DXA:  Females:  - HC: r= 0.66 (p<0.0001)  - WC: r= 0.63 (p<0.0001)  - BAI: r= 0.62 (p<0.0001)  - BMI: r= 0.70 (p<0.0001)  Males:  - HC: r= 0.66 (p<0.0001)  - WC: r= 0.72 (p<0.0001)  - BAI: r= 0.58 (p<0.0001)  - BMI: r= 0.69 (p<0.0001)  Agreement:  - Females: BAI vs DXA, MD (± 95% LoA)= 5.8 (-1.5 to 13.0)  - Males: BAI vs DXA, MD (± 95% LoA)= 0.3 (-7.0 to 6.0) | BMI showed high validity in females, and WC in males to assess body adiposity, compared with DXA. |

BAI, Body Adiposity Index; BD, body density; BIA, Bioelectrical Impedance Analysis; BMI, Body Mass Index; DXA, Dual-energy X-ray Absorptiometry; HC, Hip Circumference; NIR, Near-infrared Interactance; SKF, Skinfolds; UWW, Under Water Weighing; WC, Waist Circumference; WHR, Waist-Hip Ratio; WHtR, Waist Height Ratio; body adiposity, body fat percentage.

ANOVA, indicates analysis of variance; CI, Coefficient of Interval; LCCC, Lin’s concordance coefficient; LoA, Limits of Agreement; RMSE, root mean square error; SD, Standard Deviation; SE, Standard Error; SEE, Standard Error Estimate.

**References**

1. Sun G, Cahill F, Gulliver W, et al. Concordance of BAI and BMI with DXA in the Newfoundland population. *Obesity (Silver Spring)*. Mar 2013;21(3):499-503. doi:10.1002/oby.20009

2. Day K, Kwok A, Evans A, et al. Comparison of a Bioelectrical Impedance Device against the Reference Method Dual Energy X-Ray Absorptiometry and Anthropometry for the Evaluation of Body Composition in Adults. *Nutrients*. Oct 10 2018;10(10)doi:10.3390/nu10101469

3. Kim SG, Ko K, Hwang IC, et al. Relationship between indices of obesity obtained by anthropometry and dual-energy X-ray absorptiometry: The Fourth and Fifth Korea National Health and Nutrition Examination Survey (KNHANES IV and V, 2008-2011). *Obes Res Clin Pract*. Sep-Oct 2015;9(5):487-98. doi:10.1016/j.orcp.2014.11.002

4. Morabia A, Ross A, Curtin F, Pichard C, Slosman DO. Relation of BMI to a dual-energy X-ray absorptiometry measure of fatness. *Br J Nutr*. Jul 1999;82(1):49-55. doi:10.1017/s0007114599001117

5. Shaw KA, Srikanth VK, Fryer JL, Blizzard L, Dwyer T, Venn AJ. Dual energy X-ray absorptiometry body composition and aging in a population-based older cohort. *Int J Obes (Lond)*. Feb 2007;31(2):279-84. doi:10.1038/sj.ijo.0803417

6. Sun Q, van Dam RM, Spiegelman D, Heymsfield SB, Willett WC, Hu FB. Comparison of dual-energy x-ray absorptiometric and anthropometric measures of adiposity in relation to adiposity-related biologic factors. *Am J Epidemiol*. Dec 15 2010;172(12):1442-54. doi:10.1093/aje/kwq306

7. Cerqueira M, Amorim P, Magalhaes F, et al. Validity of body adiposity index in predicting body fat in a sample of brazilian women. *Obesity*. Dec 2013;21(12):E696-E699. doi:10.1002/oby.20543

8. Chang H, Simonsick EM, Ferrucci L, Cooper JA. Validation study of the body adiposity index as a predictor of percent body fat in older individuals: findings from the BLSA. *J Gerontol A Biol Sci Med Sci*. Sep 2014;69(9):1069-75. doi:10.1093/gerona/glt165

9. McLean KP, Skinner JS. Validity of Futrex-5000 for body composition determination. *Med Sci Sports Exerc*. Feb 1992;24(2):253-8.

10. Zhang Z-Q, Liu Y-H, Xu Y, et al. The validity of the body adiposity index in predicting percentage body fat and cardiovascular risk factors among Chinese. *Clinical Endocrinology*. Sep 2014;81(3):356-362. doi:10.1111/cen.12351
