# Supplementary material for: Criterion-Related Validity of Field-Based Methods and Equations for Body Composition Estimation in Adults: A Systematic Review
Source: Curr Obes Rep. 2022 Nov 11;11(4):336–49. doi: 10.1007/s13679-022-00488-8 (PMC9729144; doi:10.1007/s13679-022-00488-8)
Supplement: Supplementary file 8 — Supplementary file8 (DOCX 229 KB) [file 13679_2022_488_MOESM8_ESM.docx]

**Supplementary Table S5.** Overview of estimation equations for body composition in adults.

| Author | Participants | Age, range/ SD | Field test - Equations | Gold standard | Statistical methods | Results | Conclusion |
| --- | --- | --- | --- | --- | --- | --- | --- |
| ***Low quality studies*** | | | | | | | |
| Erselcan et al. 2000^1^ | Females=37  Healthy,  BMI=22.2 normal weight, 34.5 obese  *Race-ethnicity not described* | 33.9±12.2 | - SKF: biceps, subscapular, suprailiac, triceps  - BIA  - %BF equations (from BD by Siri):  1. SKF by Durnin/ Womersley (DW Sum 4SKF) | DXA | Linear regression, SEE, Bland-Altman method | Regression analysis of field test vs DXA:  Group with normal weight:  - DW Sum 4SKF: R^2^= 0.89, SEE=1.9 (p<0.0001)  - BIA: R^2^= 0.93, SEE=1.5 (p<0.0001)  Group with obesity:  - DW Sum 4SKF: R^2^= 0.75, SEE=5.1 (p<0.0001)  - BIA: R^2^= 0.84, SEE=4.0 (p<0.0001)  Agreement:  Group with normal weight:  - DW Sum 4SKF, MD (± 95% LoA)= 0.8 (1.3 to 3.6)  - BIA, MD (± 95% LoA)= -1.5 (1.6 to 3.1)  Group with obesity:  - DW Sum 4SKF, MD (± 95% LoA)= 2.5 (2.5 to 10.3)  - BIA, MD (± 95% LoA)= 2.5 (1.7 to 9.5) | DW Sum 4SKF and BIA showed very high validity to assess %BF, compared with DXA, in the group with normal weight.  DW Sum 4SKF and BIA showed high and very high validity to assess %BF, compared with DXA, in the group with obesity. However, DXA should be chosen in the group with obesity. |
| Hodgdon & Beckett 1984^2^ | Females=214  *Healthy status not described*  %BF=27  *Race-ethnicity not described* | (18-44) | - AbC, AC, CC, ChC, FC, NC, ShC, TC, WrC  - SKF: abdominal, biceps, chest, midaxillary, subscapular, suprailiac, thigh, triceps  - %BF equations (from BD by Siri):  - %BF equations:  1. Wright et al.  2. Navy equation (present study)  Cross-validation:  3. Durnin/ Womersley (DW Sum 4SKF)  4. Berrers et al.  5. Katch & McArdle  5. Brennen | UWW | Pearson correlation coefficient (r), SEE | Correlation of equations vs %BF (from BD):  1. Wright et al.: r= 0.80, SEE= 4.19 %BF (p<0.05)  2. Navy equation: r= 0.85, SEE= 3.72 %BF (p<0.05)  Cross-validation (n=146), Navy equation: r= 0.80-0.87, SEE= 4.36-4.04 %BF (p<0.05)  Cross-correlations using other equations:  1. DW Sum 4SKF: r= 0.81, SEE= 4.13 %BF (p<0.05)  2. Berrers et al.: r= 0.78, SEE= 4.38 %BF (p<0.05)  3. Wright et al: r= 0.80, SEE= 4.19 %BF (p<0.05)  4. Katch & McArdle: r= 0.75-0.81, SEE= 4.89-4.21 %BF (p<0.05)  5. Brennen: r= 0.76, SEE= 4.56 %BF (p<0.05) | Navy equation (based on AbC + NC + HC) showed high validity to assess %BF.  In this specific population, the new equation should be chosen over the previous ones compared. |
| Sloan, Burt & Blyth 1962^3^ | Females=50  Healthy  %BF=12-37  *Race-ethnicity not described* | (17-25) | - AC, ChC, GlC, TC, WC  - SKF: back arm, chest, iliac crest, thigh  - %BF equations (from BD):  1. Brozek  2. Siri  3. Rathbun & Pace | UWW | Pearson correlation coefficient (r), SEE | Correlation of body composition measures vs %BF (from BD):  - SFK, iliac crest: r= -0.71 (p<0.05)  - SKF, back arm: r= -0.68 (p<0.05)  - SKF, iliac crest+back arm: r= -0.74 (p<0.05)  Regression equation: SEE= ±0.0082 g/ml  BD to %BF:  1. Brozek: 12.13-31.70 % (MD 20.06, SD±4.63) (p<0.05)  2. Siri:13.18-36.63 % (MD 22.91, SD±5.58) (p<0.05)  3. Rathbun & Pace:11.70-37.40% (MD 22.13, SD±7.08) (p<0.05)  Regression equations:  1. Brozek: ±3 %BF (p<0.05)  2. Siri: ±3.7 %BF (p<0.05)  3. Rathbun & Pace: ±3.5 %BF (p<0.05) | SKF, especially iliac crest and back arm, showed high validity to assess %BF, compared with UWW. |
| Durnin & Rahaman 1967^4^ | Females=45  Males=60  Healthy  %BF=6-46  *Race-ethnicity not described* | (18-29)  (18-34) | - AC, CC, TC  - Bi-acromial D, Bi-iliac D  - SKF: biceps, subscapular, suprailiac, triceps  - %BF equations (from BD):  1. Brozek  2. Siri | UWW | Pearson correlation coefficient (r) | Correlation of SKF vs %BF (from BD):  - Females: r=-0.78 (p<0.001)  - Males: r=-0.83 (p<0.001)  Regression equation (values shown only by Siri):  2. Siri: ±3.0-3.5%BF (p<0.05) | SKF showed high validity to assess %BF, compared with UWW. |
| Eston et al. 2005^5^ | Females=31  Males=21  Healthy, fit-active  BMI=23.6  Caucasian | (18-36)  20.9±2.0  22.3±5.5 | - SKF: abdominal, biceps, calf, iliac crest, subscapular, thigh, triceps  SKF by Durnin/ Womersley (DW Sum 4SKF) | DXA | Forward stepwise regression, SEE | Regression analysis of SKF vs DXA:  Females:  - Calf, R^2^= 0.04, SEE=2.1 (p<0.001)  - Iliac crest, R^2^= 0.03, SEE=2.1 (p<0.001)  - Thigh, R^2^= 0.82, SEE=2.1 (p<0.001)  - DW Sum 4SKF + Sum calf+thigh, R^2^= 0.75, SEE=2.4 (p<0.001)  Males:  - Abdomen, R^2^= 0.03, SEE=1.8 (p<0.001)  - Iliac crest, R^2^= 0.09, SEE=1.8 (p<0.001)  - Thigh, R^2^= 0.79, SEE=1.8 (p<0.001)  - DW Sum 4SKF + Sum calf+thigh, R^2^= 0.80, SEE=1.9 (p<0.001) | Lower limb SKF showed high validity to assess %BF, compared with DXA.  Conventional DW Sum 4SKF is improved by adding thigh and calf SKF, independently or in combination. |
| Friedl et al. 1997^6^ | Males=496  Healthy  BMI=24.8  *Race-ethnicity not described* | (<40) | - AbC, NC  - %BF equations:  1. Marine Corps (Wright, Dotson, and Davis)  2. Navy (Hodgdon/ Beckett)  3. Army (Vogel et al.)  4. New regression equation:  %BF = (0.771 x AbC-NC) (0.132 x height) + 4.29 | DXA | Linear regression, SEE | Regression analysis of equations vs %BF:  - Marine Corps equation: R^2^= 0.80, SEE= 3.27 (p<0.01)  - Navy equation: R^2^= 0.82, SEE= 3.13 (p<0.01)  - Army equation: R^2^= 0.81, SEE= 3.15 (p<0.01)  - New regression equation: R^2^= 0.82, SEE= 3.10 (p<0.01) | All equations showed very high validity to assess %BF, being the new equation (based on AbC + NC) the most valid and accurate.  It is recommended to add AbC in the equations because it has a good relationship with the %BF. |
| Haisman 1970^7^ | Males=55  Healthy  %BF=12.5  *Race-ethnicity not described* | 22.6±2.2 | - SKF: abdominal, biceps, chest, subscapular, suprailiac, triceps  - %BF equations (from BD):  1. Siri  - %BF equations:  1. Edwards & Whyte  2. Adam et al.  3. Durnin & Rahaman  4. Chinn & Allen  5. Pascale et al.  6. Brozek & Keys  7. Best  8. Equation of the present study | UWW | Pearson correlation coefficient (r), SEE | Correlation of SKF vs %BF (from BD):  - Single/combination SKF: r= 0.63-0.77, SEE= 0.008-0.006 (p<0.05)  SKF equations:  1. Edwards & Whyte: r= 0.71 (p<0.05)  2. Adam et al.: r= 0.65 (p<0.05)  3. Durnin & Rahaman: r= 0.76 (p<0.05)  4. Chinn & Allen: r= 0.71 (p<0.05)  5. Pascale et al.: r= 0.76 (p<0.05)  6. Brozek & Keys: r= 0.69 (p<0.05)  7. Best: r= 0.75 (p<0.05)  8. Equation of the present study: r= -0.81, SEE= 0.007 (p<0.05) | SKF (Sum 4SKF or 9SKF), showed high validity to assess %BF, compared with UWW.  Equations by Haisman (present study, Sum 4SKF), Durnin & Rahaman, Pascale et al. and Best showed high validity to assess %BF. |
| Katch & McArdle 1973^8^ | Females=69  Males=53  Healthy  %BF=26 females; 15 males  Caucasian | 20.3±1.8  19.3±1.5 | - AbC, AC, AkC, CC, ChC, FC, GlC, HdC, KC, ShC, TC, WrC  - AkD, Bi-acromial D, Bi-iliac D, Bitrochanteric D, ChD, ED, KD, WrD  - SKF: subscapular, suprailiac, thigh, triceps, umbilicus  - %BF equations (from BD, by Brozek) | UWW | Pearson correlation coefficient (r), SEE | Correlation of equations vs %BF (from BD):  Females:  - SKF, r= 0.77 (p<0.05)  - Circumferences, r= 0.80 (p<0.05)  - SKF+circumferences, r= 0.78 (p<0.05)  - SKF (scapula and iliac)+ED+TC, r= 0.84, SEE= ± 3.6%BF (p<0.05)  Males:  - SKF, r= 0.86 (p<0.05)  - Circumferences: r= 0.86 (p<0.05)  - SKF (triceps and scapula)+AbC+FC, r= 0.89; SEE= ± 2.9%BF (p<0.05) | All measures showed high validity to assess total %BF, compared with UWW.  In females, equation based on SKF (scapula and iliac) + ED + TC best predicted %BF.  In males, equation based on SKF (triceps and scapula) + AbC + FC best predicted %BF. |
| Katch & McArdle 1975^9^ | Males=53  Healthy  %BF=26 females; 15 males  *Race-ethnicity not described* | (17-24)  19.3±1.5 | - AbC, ChC, NC  - Bi-iliac D  - SKF: midanterior, subscapular, suprailiac, thigh, triceps, umbilicus  - %BF equations (from BD, by Brozek):  1. Sloan  2. Wilmore & Behnke  - %BF equations:  1. Steinkamp et al.  2. Yuhasz | UWW | Pearson correlation coefficient (r), SEE, CE | Correlation of equations vs %BF (from BD):  1. Sloan: r=0.81, SEE=0.008 (p<0.05)  2. Wilmore & Behnke: r=0.83, SEE=0.008 (p<0.05)  Equation correlations:  - Steinkamp et al.: r= 0.81, SEE= 3.27, CE= -0.71 (p<0.01)  - Yuhasz: r= 0.84, SEE= 3.02, CE= 1.12 (p<0.01) | Both equations (based on AbC + ChC +NC + bi-iliac D + abdomen, thigh and scapula SKF) showed high validity to assess total %BF. |
| Wilmore & Behnke 1969^10^ | Males=133  Healthy  %BF=14  *Race-ethnicity not described* | (17-37)  22.0±3.1 | - AbC, AC, AkC, ChC, Deltoid C, FC, HC, HdC, KC, NC, ShC, TC  - AD, AkD, Biacromial D, Bideltoid D, Bi-iliac D, Bi-trochanter D, ChD, KD, ED, WrD  - Foot length, Hand length  - SKF: abdominal, midaxillary, thigh, triceps  - %BF equations (from BD):  1. Brozek  2. Siri  3. Rathbun & Pace  - %BF equation:  1. Yuhasz  2. Equation of the present study | UWW | Pearson correlation coefficient (r), SEE | Correlations of SKF vs %BF (from BD):  - Sum SKF: r= -0.78 (p<0.05)  - Diameters: r= -0.04 to -0.42 (p<0.05)  - Circumferences: r= -0.16 to -0.73 (p<0.05)  Equation correlations:  - Sum SKF: r= 0.80-0.81, SEE= 0.008-0.007 (p<0.05)  - Diameters+circumferences: r= 0.79-0.85, SEE= 0.007 (p<0.05)  - SKF+diameters+circumferences: r= 0.80-0.87, SEE= 0.007-0.006 (p<0.05)  - Yuhasz equation vs equations in this study: r= 0.78 (p<0.05) | All measures showed high validity to assess total %BF, compared with UWW.  Equations based on SKF + diameters + circumferences best predicted %BF. |
| Wilmore & Behnke 1970^11^ | Females=128  Healthy  %BF=26  *Race-ethnicity not described* | (18-48)  21.4±3.8 | - AbC, AC, AkC, ChC, Deltoid C, FC, HC, HdC, KC, NC, ShC, TC  - AD, AkD, Biacromial D, Bideltoid D, Bi-iliac D, Bi-trochanter D, ChD, KD, ED, WrD  - Foot length, Hand length  - SKF: abdominal, midaxillary, thigh, triceps  %BF equations (from BD):  1. Brozek  2. Siri  3. Rathbun & Pace  - %BF equations:  1. Sloan  2. Katch & Michael  3. Equation of the present study | UWW | Pearson correlation coefficient (r), SEE | Correlation of body composition measures vs %BF (from BD):  - SKF: r= -0.17 to -0.58 (p<0.05)  - Diameters: r= -0.07 to -0.40 (p<0.05)  - Circumferences: r= -0.02 to -0.61 (p<0.05)  Equation correlations:  - Sum SKF: r= 0.68, SEE=0.007 (p<0.05)  - Diameters+circumferences: r= 0.74, SEE=0.007 (p<0.05)  - SKF+diameters+circumferences: r= 0.75, SEE=0.007 (p<0.05)  1. Sloan equation vs equations in this study: r= 0.58 vs r= 0.74 (p<0.05)  2. Katch & Michael vs equations in this study: r= 0.64 vs r= 0.70 (p<0.05) | All measures showed very low to moderate validity to assess total %BF, compared with UWW.  Equations based on SKF + diameters + circumferences best predicted %BF. |
| Temple et al. 2014^12^ | Females=43  Healthy  BMI=26.2  Total sample, normal weight, overweight  Caucasian | (21-55)  37.3±9.7 | - SKF: abdominal, biceps, chest, midaxillary, subscapular, suprailiac, thigh, triceps  - %BF equations (from BD by Siri):  1. Jackson, Pollock and Ward, three, four and seven-SKF equations (JPW3, JPW4 and JPW7, respectively)  2. Durnin/ Womersley (DW Sum 4SKF) | ADP | Linear regression (bias change-point, %), CVRSME | Regression analysis of equations vs %BF (Bias change-point, %):  - DW Sum 4SKF: 32%, *bias=0, for total sample; 35% for group with normal weight; 34% for group with overweight.  - JPW3: 19% for total sample; 16% for group with normal weight; 29% for group with overweight.  - JPW4: 18% for total sample; 15% for group with normal weight; 27% for group with overweight.  - JPW7: 15% for total sample; 14% for group with normal weight; 25% for group with overweight.  **Bias data is only shown for this outcome.*  Accuracy:  - DW Sum 4SKF: CVRSME= 0.16 for total sample; 0.29 for group with normal weight; and 0.35 for group with overweight.  - JPW3: CVRSME= 0.29 for total sample; 0.24 for group with normal weight; and 0.31 for group with overweight.  - JPW4: CVRSME= 0.35 for total sample; 0.26 for group with normal weight; and 0.37 for group with overweight.  - JPW7: CVRSME= 0.35 for total sample; 0.30 for group with normal weight; and 0.36 for group with overweight. | All measurements showed a positive bias, which decreased as %BF increased.  The DW Sum 4SKF equation is the most accurate equation for the estimation of %BF in both females with normal-weight and with overweight. |
| ***High quality studies*** | | | | | | | |
| Aandstad et al. 2014^13^ | Females=26  Males=39  Healthy  BMI=23.3  Caucasian | (18-30)  21±4  (19-27)  22±2 | - SKF: abdominal, biceps, chest, subscapular, suprailiac, thigh, triceps, in males.  - 6 sites in females, except chest.  - %BF equations (from BD by Siri), SKF:  1. Durnin/ Womersley (DW Sum 4SKF)  2. Jackson/Pollock (JP Sum 3SKF), in males  3. Jackson, Pollock and Ward (JPW Sum 3SKF), in females  4. Lohman, in females and males (Sum 5SKF)  5. Slaughter et al., in females and males (Sum 6SKF)  - %BF equations, BIA:  1. Deurenberg et al., in females and males  2. Gray et al., in females and males  3. Kotler et al., in females and males  4. Kyle et al., in females and males  5. Lohman, in females and males  6. Lukaski et al., in females and males  7. Segal et al. generalized equation (GEN), in females and males  8. Segal et al. fatness specific equation (FSE)  9. Sun et al., in females and males  10. van Loan et al., in females and males  - %BF equations (SFK and BIA)  1. Guo et al., in males  2. Yannakoulia et al., in females | DXA | Pearson correlation coefficient (r), ICC, Bland-Altman method | Correlation of equations vs %BF:  Females:  - SKF equations: r= 0.77 to 0.88; ICC (95% CI)= 0.73 to 0.88 (0.49-0.95)  - BIA equations: r = 0.84 to 0.92; ICC (95% CI)= 0.80 to 0.92 (0.60-0.96)  - SKF and BIA equation: r= 0.86; ICC (95% CI)= 0.86 (0.71-0.93)  Males:  - SKF equations: r= 0.86 to 0.88; ICC (95% CI)= 0.82 to 0.87 (0.69-0.93)  - BIA equations: r= 0.76 to 0.87; ICC (95% CI)= 0.66 to 0.84 (0.43-0.91)  - SKF and BIA equation: r= 0.92; ICC (95% CI)= 0.69 (0.48-0.82)  Agreement:  Females:  - SKF equations: MD (± 95% LoA)= 0.4 to 6.6% (4.7 to 6.6)  - BIA equations: MD (± 95% LoA)= -3.2 to 3.2% (4.0 to 8.0)  Males:  - SFK equations: MD (± 95% LoA)= -4.8 to 3.4% (3.5 to 5.2)  - SKF-BIA equations: MD (± 95% LoA)= -2.2 to 4.4% (4.4 to 8.5) | Both, SKF equations and SKF-BIA equations showed high/very high validity to assess %BF. |
| Aristizabal et al. 2008^14^ | Females=52  Healthy  BMI=22.3  Colombian | (18-40)  28.0±6.6 | - SKF: abdominal, biceps, iliocrestal, subscapular, supraspinal, thigh, triceps  - %BF equations (from BD by Siri):  1. Durnin/ Womersley (DW Sum 4SKF)  2. Jackson, Pollock and Ward (JPW Sum 3SKF)  3. Ramirez/Torun ((%BF=-15,471 + (tricipital SKF*0,332) + (subescapular SKF*0,154) + (abdominal SKF*0,119) + (HC*0,356)) | UWW | Pearson correlation coefficient (r), Bland-Altman method | Correlation of equations vs %BF:  - DW Sum 4SKF equation: r= 0.62 (p<0.001)  - JPW Sum 3SKF equation: r= 0.71 (p<0.001)  - Ramirez/Torun equation: r= 0.67 (p<0.001)  Agreement:  - DW Sum 4SKF equation: MD (± 95% LoA)= -4.67 (-13.25 to 3.90)  - JPW Sum 3SKF equation: MD (± 95% LoA)= 3.81 (-4.44 to 12.06)  - Ramirez/Torun equation: MD (± 95% LoA)= -2.49 (-11.00 to 6.03) | The DW Sum 4SFK and Ramirez/Torun equations showed moderate validity to predict %BF, while JPW Sum 3SKF equation showed high validity. |
| Aristizabal et al. 2018^15^ | Females=151  Healthy  BMI= 23.6±3.2  Colombian  Development group, validation group | (18-59) | - AbC, AC, CC, HC, TC, WC  - SKF: abdominal, axillar, biceps, calf, iliocrestal, subscapular, thigh, triceps  - %BF equations (from BD by Siri):  1. Durnin/ Womersley (DW Sum 4SKF)  2. Jackson, Pollock and Ward (JPW Sum 3SKF)  3. Ramirez/Torun  4. Equations of the present study (3 equations) | UWW | Pearson correlation coefficient (r), regression analysis, SEE, ICC | Equations development:  - Equation 1: R^2^= 0.72, SEE= 3.12 (p<0.001)  - Equation 2: R^2^= 0.72, SEE= 3.08 (p<0.001)  - Equation 3: R^2^= 0.66, SEE= 3.44 (p<0.001)  Equations validation:  - Equation 1: R^2^= 0.71, SEE= 2.81 (p<0.001)  - Equation 2: R^2^= 0.67, SEE= 3.06 (p<0.001)  - Equation 3: R^2^= 0.55, SEE= 3.55 (p<0.001)  Correlation of equations vs %BF:  - DW Sum 4SFK equation: r= 0.75, ICC= 0.51 (p<0.001)  - JPW Sum 3SFK equation: r= 0.77, ICC= 0.53 (p<0.001)  - Ramirez/Torun equation: r= 0.77, ICC= 0.75 (p<0.001)  - Equation 1: r= 0.81, ICC= 0.77 (p<0.001)  - Equation 2: r= 0.79, ICC= 0.76 (p<0.001)  - Equation 3: r= 0.77, ICC= 0.73 (p<0.001) | All new equations showed high validity to assess total %BF.  Equation 1 (based on triceps and thigh SKF + AbC) was the most accurate to assess total %BF.  DW Sum 4SFK and Ramirez/Torun equations overestimated %BF, while JPW Sum 3SKF equation underestimated %BF in this population. |
| Balas-Nakash et al. 2010^16^ | Females=86  Healthy  BMI=30.7  Mexican | (42-72)  54.8±6.9 | - BIA  - %BF equations:  1. NHANES III  2. Macias, et al. | DXA | Pearson correlation coefficient (r), CE, SEE, TE Bland-Altman method | Correlation of equations vs %BF:  - BIA- NHANES III equations: r= 0.80 (p=0.001), CE= -2.61, SEE= 3.75, TE= 94.98  - BIA-Macias, et al. equations: r= 0.80 (p=0.001), CE=- 2.61, SEE= 3.75, TE= 94.98  Agreement:  - BIA- NHANES III equations: MD (± 95% LoA)= -2.0 (-8.0 to 4.0)  - BIA-Macias, et al. equations: MD (± 95% LoA)= -2.0 (-8.5 to 4.5) | Both BIA equations showed high validity to assess %BF. |
| Ball et al. 2004^17^ | Females=150  Healthy  BMI=23.0  Caucasian, Hispanic, African American, Asian | (18-55)  28.7±8.6 | - SKF: abdominal, chest, midaxillary, subscapular, suprailiac, thigh, triceps  - %BF equations (from BD by Siri and Brozek):  1. Jackson, Pollock and Ward, seven-SKF (JPW 7SKF)  2. Jackson, Pollock and Ward, three-SKF (JPW 3SKF)  4. DXA criterion | DXA | Pearson correlation coefficient (r), regression analysis, SEE, Bland-Altman method | Correlation of equations vs %BF:  - JPW 7SKF: r= 0.92 (p<0.01)  - JPW 3SKF: r= 0.92 (p<0.01)  - JP 3SKF: r= 0.89 (p<0.01)  Regression analysis for DXA criterion equation:  - R^2^= 0.86, SEE= 2.5 (p<0.01)  Cross-validation of DXA criterion equation:  - R^2^= 0.93, SEE= 2.1 (p<0.01)  Agreement:  - MD (± 95% LoA)= 0.2 (-4.8 to 4.9) | The DXA criterion equation, JPW and JP (Sum 7SKF and Sum 3SKF) equations showed very high validity to assess %BF. |
| Brozek & Keys 1951^18^ | Males=255  Healthy  %BF=10 college, 24 middle-aged  *Race-ethnicity not described*  2 age groups (college students and middle-aged men) | (18-55)  20.3±1.9  49.0±2.8 | - AbC, ChC  - SKF: abdominal, chest, scapular, thigh, triceps  - %BF equations (from BD):  1. Rathbun & Pace | UWW | Pearson correlation coefficient (r), SEE | Correlation of SKF vs %BF:  - Abdominal: r= -0.84, SEE= 0.008 in college; r= -0.60, SEE= 0.010 in middle-aged (both, p<0.05)  - Chest: r= -0.86, SEE= 0.007 in college; r= -0.68, SEE= 0.009 in middle-aged (both, p<0.05)  - Scapular: r= -0.81, SEE= 0.009 in college; r= -0.68, SEE= 0.009 in middle-aged (both, p<0.05)  - Triceps: r= -0.83, SEE= 0.008 in college; r= -0.65, SEE= 0.010 in middle-aged (both, p<0.05)  - Thigh: r= -0.75, SEE= 0.009 in college; r= -0.63, SEE= 0.010 in middle-aged  Best equations:  - College students, Equation 3: r= 0.87, SEE= 0.007 (p<0.05)  - Middle-aged men, Equation 4: r= 0.74, SEE= 0.008 (p<0.05) | Chest SKF was higher correlated with %BF (from BD), compared with UWW.  Both SKF equations showed high validity to assess %BF. |
| Cui et al. 2014^19^ | Females=4666  Males=5268  Healthy  BMI=26.2  Normal weight (NW), overweight (OW) and obese (OB)  Mexican Americans (MA), Non-Hispanic Whites (NHW), Non-Hispanic Blacks (NHB) | (20-84)  46.6±0.4  44.3±0.3 | - WC, WHtR  - SKF: subscapular, triceps  - BMI  - %BF equations (from BD by Siri):  1) SKF: Durnin/Womersley (DW Sum 4SKF); Hassager et al.; Slaughter et al.  2) Height and weight and/or BMI: Gallagher et al.; Gomez-Ambrosi et al.; Heitmann; Jackson/Pollock; Noppa et al. (females); Pasco et al.; Rush et al. (females); Smith and Boyce (females); Visser et al.  3) WC or WHtR: Kagawa et al.; Lean et al.  4) Multiple anthropometric variables: Chapman et al.; Ramirez-Zea et al.; Svendsen et al.; Wilmore & Behnke | DXA | Regression analysis, RMSE, MSD | Regression analysis of equations vs %BF:  Females (entire sample):  1) SKF, R^2^= 0.63 to 0.66 (p<0.05); RMSE= 3.6 to 4.0; MSD= -4.5 to -3.1  2) BMI, R^2^= 0.55 to 0.72 (p<0.05); RMSE= 3.3 to 4.3; MSD= -8.5 to 3.3  3) WC or WHtR, R^2^= 0.55 to 0.56 (p<0.05); RMSE= 4.4; MSD= 0.2 to 0.5  4) Multiple, R^2^= 0.46 to 0.69 (p<0.05); RMSE= 3.4 to 4.2; MSD= -9.9 to 1.3  Males (entire sample):  1) SKF, R^2^= 0.61 to 0.77 (p<0.05); RMSE= 3.0 to 3.4; MSD= -5.3 to -0.9  2) BMI, R^2^= 0.47 to 0.60 (p<0.05); RMSE= 3.4 to 3.7; MSD= -4.1 to 3.7  3) WC or WHtR, R^2^= 0.68 (p<0.05); RMSE= 3.1; MSD= 0.5 to 1.1  4) Multiple, R^2^= 0.46 to 0.76 (p<0.05); RMSE= 2.7 to 3.4; MSD= -7.7 to 1.1  BMI status (not sex distinction):  1) SKF, R^2^= 0.33 to 0.60 for NW; R^2^= 0.03 to 0.42 for OW; R^2^= 0.02 to 0.36 for OB (all, p<0.05)  2) BMI, R^2^= 0.18 to 0.38 for NW; R^2^= 0.02 to 0.20 for OW; R^2^= 0.08 to 0.30 for OB (all, p<0.05)  3) WC or WHtR, R^2^= 0.21 to 0.51 for NW; R^2^= 0.04 to 0.39 for OW; R^2^= 0.12 to 0.41 for OB (all, p<0.05)  4) Multiple, R^2^= 0.30 to 0.60 for NW; R^2^= 0.05 to 0.50 for OW; R^2^= 0.04 to 0.51 for OB  Sex-race/ethnicity-specific (not sex distinction):  1) SKF, R^2^= 0.46 to 0.63 for MA; R^2^= 0.53 to 0.68 for NHW; R^2^= 0.55 to 0.73 for NHB (all, p<0.05)  2) BMI, R^2^= 0.49 to 0.62 for MA; R^2^= 0.51 to 0.70 for NHW; R^2^= 0.52 to 0.67 for NHB (all, p<0.05)  3) WC or WHtR, R^2^= 0.47 to 0.66 for MA; R^2^= 0.52 to 0.68 for NHW; R2= 0.48 to 0.71 for NHB (all, p<0.05)  4) Multiple, R^2^= 0.43 to 0.69 for MA; R^2^= 0.56 to 0.73 for NHW; R^2^= 0.54 to 0.75 for NHB (all, p<0.05) | In females, equations using BMI, showed higher validity to assess %BF than the other equations.  In males, equations using SFK and WC, showed higher validity to assess %BF than the other equations.  Equations using BMI or WC underestimated %BF in adults with normal weight but slightly overestimate it in adults with obesity.  Equations using SKF underestimated %BF more in females with obesity than in females with non-obesity.  These equations did not perform better in one race-ethnicity than that in others. |
| Davidson et al. 2011^20^ | Females=1002  Males=673  Healthy  BMI=23.2 (C), 27.3 (AA), 27.8 (H), 21.9 (A)  Caucasian (C), African, American (AA), Hispanic (H), Asian (A) | (18-110)  50.7±19.1  47.7±18.8 | - WC  - SKF: biceps, subscapular, suprailiac, triceps  - %BF equations (from BD by Siri):  1. Durnin/ Womersley (DW Sum 4SKF), age-specific  2. DW all-age  3. Sex and race: DW equation+age, height, weight and WC | DXA | Linear regression, SEE | Females:  - Logarithm of Sum SKF: 35.884 logSF - 33.709, R^2^= 0.75, SEE= 4.56 (p<0.05)  - New DW equation sex-race/ethnicity-specific prediction equations:  R^2^= 0.80, SEE= 3.94 (p<0.05) for C  R^2^= 0.80, SEE= 3.98 (p<0.05) for AA  R^2^= 0.73, SEE= 3.71 (p<0.05) for H  R^2^= 0.73, SEE= 3.43 (p<0.05) for A  Males:  - Logarithm of Sum SKF: 30.729 logSF - 31.122, R^2^= 0.71, SEE= 4.12 (p<0.05)  - New DW equation sex-race/ethnicity-specific prediction equations:  R^2^= 0.77, SEE= 3.53 (p<0.05) for C  R^2^= 0.79, SEE= 4.00 (p<0.05) for AA  R^2^= 0.80, SEE= 3.43 (p<0.05) for H  R^2^= 0.64, SEE= 3.45 (p<0.05) for A | New sex-race/ethnicity-specific prediction equations (based on Durnin/ Womersley Sum 4SKF + WC, age, height and weight) showed high validity. However, overestimated body fat by DXA in all but C males and AA females. |
| Demura et al. 2002^21^ | Males=50  Healthy  %BF=*see results*  Japanese | (18-27) | - SKF: subscapular, triceps  - BIA  -%BF equations (from BD by Brozek):  1. BIA, hand-foot method (H-F)  2.BIA, foot-foot method (F-F)  3. SKF method | UWW | Linear regression, Bland-Altman method | %BF values:  - BIA, H-F= 17.8±3.8  - BIA, F-F= 17.7±4.8  - SKF= 12.6±2.7  - UWW= 14.5±5.5  Prediction equations vs %BF:  - BIA, H-F: R^2^= 0.92 (p<0.05)  - BIA, F-F: R^2^= 0.50 (p<0.05)  - SKF: R^2^= 0.51 (p<0.05)  Agreement:  - H-F, MD (± 95% LoA)= 3.3 (-0.03 to 1.58)  - F-F, MD (± 95% LoA)= 3.2 (0.23 to 1.84)  - SKF, MD (± 95% LoA)= 1.9 (-1.61 to 0.63) | Hand-foot BIA analysis and SKF showed very high validity to assess %BF, compared with UWW. |
| Dioum et al. 2005^22^ | Females=196  Healthy  BMI=22.5  African | (18-56)  29.5±8.7 | - SKF: biceps, subscapular, suprailiac, triceps  - %BF equations (from BD by Siri):  1. Durnin/ Womersley (DW Sum 4SKF) equation coupled with Siri equation (DWS)  2. Durnin/ Womersley (DW Sum 4SKF) equation coupled with Black-specific equation (DWB) | ADP | Pearson correlation coefficient (r), Pure Error (PE), Bland Altman method | Correlation of equations vs %BF:  - DWS equation: r= 0.50, PE= 7.9 (p<0.00001)  - DWB equation: r= 0.53, PE= 6.5 (p<0.00001)  Agreement:  - DWS equation, MD (± 95% LoA)= 6.5 (-2.3 to 15.3)  - DWB equation, MD (± 95% LoA)= 4.6 (-4.2 to 13.4) | Both DW Sum 4SKF equations showed moderate validity and underestimate %BF. |
| Durnin & Womersley 1974^23^ | Females=272  Males=209  Healthy  %BF=14-52 females; 7-50 males  *Race-ethnicity not described*  Age-groups: 16-19, 20-29, 30-39, 40-49 years | (16-72) | - AC, CC, TC  - SKF: biceps, subscapular, suprailiac, triceps  - %BF equations (from BD):  1. Brozek  2. Siri  Different Sum SKF | UWW | Regression analysis, SEE | SKF vs %BF (from BD):  - Females: R^2^= 0.70-0.90, SEE= 0.012-0.009 (p<0.05)  - Males: R^2^= 0.70-0.90, SEE= 0.010-0.007 (p<0.05) | Durnin/ Womersley equations showed high to very high validity to assess %BF, compared with UWW.  Although there is little error between even one single measured SKF or two and three SKF, the likelihood of a large error can be reduced by using the Sum 4SKF. In addition, the best-fit regression is derived from Sum 4SKF. |
| Friedl et al. 2001^24^ | Females=150  Healthy  BMI=23.3  Black (B), Hispanic (H), Non-Hispanic White (NHW) | (17-33)  21.4±3.6 | - AC, AbC, CC, ChC, FC, NC, TC, WC  - SKF: abdominal, biceps, calf, chest, midaxillary, subscapular, suprailiac, thigh, triceps  - %BF equations (from BD by Siri):  1. Marines  2. Navy  3. Army  4. Durnin/ Womersley (DW Sum 4SKF)  5. Jackson, Pollock and Ward (JPW Sum 7SKF) | DXA | ME (residual SD), F ratio of PE, sensitivity, specificity | Correlation of body composition measures vs %BF:  - Marines: ME= 6.7 (2.8) B, 8.3 (3.0) H, 7.1 (2.8) NHW; PE (all combined)= 8.1%; F ratio (all combined)= 8.08 (p<0.05)  - Navy: ME= 1.3 (2.1) B, 0.9 (1.9) NHW, 2.4 (1.9) H; PE (all combined)= 3.9%; F ratio (all combined)= 5.95 (p<0.05)  - Army: ME= 2.0 (2.3) B, 3.0 (2.1) H, 2.8 (2.2) NH; PE (all combined)= 4.8%; F ratio (all combined)= 4.80 (p<0.05)  - DW Sum 4SKF: ME= -0.8 (2.0) B, -0.1 (1.8) H, -0.1 (1.8) NHW; PE (all combined)= 3.5%; F ratio (all combined)= 3.90 (p<0.05)  - JPW Sum 7SKF: ME= 5.0 (2.4) B, 5.9 (2.5) H, 5.1 (2.4) NHW; PE (all combined)= 5.9%; F ratio (all combined)= 3.49 (p<0.05)  Sensitivity: 12% to 55%  Specificity: 48% to 86% | All the equations had relatively low sensitivity and specificity.  The Navy and DW Sum 4SKF equations had the lowest error in the prediction of %BF. |
| Gallagher et al. 1996^25^ | Females=394  Males=312  Healthy  Whites (females, BMI=23.3; males, BMI=25.2)  Blacks (females, BMI=27.0; males, BMI=25.8) | (20-94)  50.1±17.1  47.7±17.5 | - HC, WC, WHR  - Tibia/total body length  - BMI  - BF equation:  2.513 x body volume - 0.739 x total body water + 0.947 x total body bone mineral mass - 1.79 x body weight | DXA, D_2_O, ^3^H_2_O, UWW (4C) | Pearson correlation coefficient (r), multiple regression analysis | Correlation of age and body composition measures correlations vs BMI:  - Females, Whites: r= 0.21-0.87 (all, p<0.05); Blacks: r= 0.23-0.89 (all, p<0.05), height, r= -0.07 (p>0.05)  - Males, Whites: r= 0.44-0.84 (all, p<0.05), age and height, r= -0.04 to 0.07 (p<0.05); Blacks: r= 0.25-0.85 (all, p<0.05), height, r= 0.02 (p>0.05)  Regression analysis, BF vs age and BMI:  - Females, Whites: R^2^= 0.56 (p<0.001); Blacks: R^2^= 0.58 (p<0.05)  - Males, Whites: R^2^= 0.44 (p<0.001); Blacks: R^2^= 0.52 (p<0.05)  Regression analysis, BF vs age, sex and BMI:  - Whites: R^2^= 0.64 (p<0.001)  - Blacks: R^2^= 0.72 (p<0.001)  Regression analysis, BF vs age, sex, ethnicity and BMI: R^2^= 0.67 (p<0.001) | BMI showed moderate to high validity to assess BF. Nevertheless, BMI is age and sex dependent as an indicator of BF, but is ethnicity independent in White and Black adults. |
| Gallagher et al. 2000^26^ | Females=1013  Males=613  Healthy  White (BMI=25.5), African American (BMI=23.1), and Asian (BMI=26.5) | 48.1±16.7  48.7±17.4 | - BMI  - %BF equations:  1. Equation for White and African American  2. Equations for Asian females and Asian males | DXA, D_2_O, ^3^H_2_O, UWW (4C) | Pearson correlation coefficient (r), multiple regression analysis | Correlation of BMI vs %BF:  - Whole sample: r= 0.68-0.89 (all, p<0.001)  Regression analysis of %BF equations vs DXA/ 4C:  - Asian, females: R^2^= 0.88 (p<0.05), SEE= 2.91 %BF  - Asian, males: R^2^= 0.77 (p<0.05), SEE= 3.49 %BF  - White and African American  adults: R^2^= 0.86 (p<0.05), SEE= 4.98 %BF | BMI showed moderate to high validity to assess %BF.  BMI is ethnicity dependent, especially in Asian population. |
| Goel et al. 2008^27^ | Females=76  Males=95  Healthy  BMI=22.9  Asian Indians | (18-50)  32.2±9.5 | - WC, HC, Mid-thigh C, CC  - SKF: anterior axillary, biceps, calf, subscapular, suprailiac, thigh, triceps  - %BF equation= 42.42 + 0.003 × age + 7.04 × gender + 0.42 × triceps SKF + 0.29 × WC − 0.22 × weight − 0.42 × height | DXA | Pearson correlation coefficient (r), multiple regression analysis, ME | Correlation of body composition measures vs %BF:  - Triceps: r= 0.82 (p<0.05)  - Thigh: r= 0.78 (p<0.05)  - Calf: r= 0.69 (p<0.05)  - WC: r= 0.53 (p<0.05)  Regression analysis of the predictive equation:  - R^2^= 0.86 (p<0.05), ME= -0.60, CI= -9.60 to 7.82 | This equation (based on triceps SKF + WC) showed very high validity to assess %BF. |
| Gómez-Ambrosi 2011^28^ | Females=4356  Males=2154  Healthy  BMI=32.0  White | (18-80)  44±14  47±14 | - WHR, WHtR  - BMI  - BAI  - CUN-BAE, Rohrer index  - New equation (CUN-BAE):  -44.988 + (0.503xage) + (10.689xsex) + (3.1723BMI) – (0.026x  BMI2) + (0.181 x BMI x sex) – (0.02 x  BMI x age) – (0.005 x BMI2 x sex) + (0.00021 3 BMI2 x age) | ADP | Pearson correlation coefficient (r), SEE, Bland-Altman method | Correlation of body composition measures correlations vs %BF:  Females (all, p<0.001):  - WHR: r= 0.82  - WHtR: r= 0.49  - BAI: r= 0.81  - BMI: r= 0.84  - CUN-BAE: r= 0.89, SEE= 4.36 %BF  Males (all, p<0.001):  - WHR: r= 0.84  - WHtR: r= 0.47  - BAI: r= 0.35  - BMI: r= 0.77  - CUN-BAE: r= 0.81, SEE= 5.20 %BF  Agreement, %BF CUN-BAE vs ADP:  MD (± 95% LoA)= -0.64 (-9.86 to 8.58) | The CUN-BAE showed high validity to assess %BF.  In females, all measures (except for WHtR) showed high validity to assess %BF.  In males, WHR and BMI high validity, while WHtR and BAI low validity to assess %BF. |
| Gomez et al. 2017^29^ | Females=3359  Males=3354  Healthy  BMI=23.9  Chilean | (17-27)  20.7±2.0  20.6±2.4 | - WC, WHtR  - Equations for fat mass (FM):  Females:  1. FM= −13,216,917 + 461,302 x weight + 91,898 x WC  2. FM= −14,144,220 + 464,061 x weight + 16189,297 x WHtR  Males:  1. FM= −35,997,486 + 232,285 x weight + 432,216 x WC  2. FM= −37,671,303 + 309,539 x weight + 66028,109 x WHtR | DXA | Regression analysis, SEE, Bland-Altman method | Regression analysis of equations:  Equations for females:  - 1. R^2^= 0.70, SEE= 4.6, p<0.05)  - 2. R^2^= 0.70, SEE= 4.6, p<0.05)  Equations for males:  - 1. R^2^= 0.73, SEE= 4.1, p<0.05)  - 2. R^2^= 0.76, SEE= 3.8, p<0.05)  Agreement:  - Females:  1. MD (± 95% LoA)= 0.1 (-7.3 to 7.0)  2. MD (± 95% LoA)= -0.1 (-7.1 to 6.8)  - Males:  1. MD (± 95% LoA)= 0.0 (-9.0 to 9.1)  2. MD (± 95% LoA)= 0.0 (-9.0 to 9.0) | The four equations (based on WC and WHtR) showed high validity to assess fat mass in young population. |
| Jackson & Pollock 1978^30^ | Males=308  Healthy  %BF=17.8  *Race-ethnicity not described* | (18-61)  32.6±10.8 | - FC, WC  - SKF: abdomen, axilla, triceps, chest, subscapula, supra-iliac, thigh  - %BF equations (from BD):  1. Brozek  2. Siri  Different Sum SKF | UWW | Regression analysis, SEE | Correlation between Sum 7SKF and Sum 3SKF: r= 0.98 (p<0.01)  Regression analysis of equations vs %BF (from BD):  - Sum 7SKF: r= 0.92, SEE= 0.007 g/ml (p<0.01)  - Sum 3SKF: r= 0.91, SEE= 0.007 g/ml (p<0.01)  Cross-validation of these equations:  - All equations: r=0.90, SEE<0.0900 g/ml (p<0.01) | Both equations (composed by Sum 3SKF or Sum 7SKF) showed very high validity to assess %BF. |
| Jackson, Pollock & Ward 1980^31^ | Females=249  Healthy  %BF=24.1  *Race-ethnicity not described* | (18-55)  31.4±10.8 | - GlC  - SKF: abdomen, axilla, chest, subscapula, suprailiac, thigh, triceps  - %BF equations (from BD):  1. Brozek  2. Siri  Different Sum SKF | UWW | Regression analysis, SEE | Correlation between Sum 7SKF, Sum 4SKF and Sum 3SKF: r= 0.97 (p<0.01)  Regression analysis of equations vs %BF (from BD):  - Sum 7SKF: r= 0.87, SEE= 0.007 g/ml (p<0.01)  - Sum 4SKF: r= 0.87, SEE= 0.008 g/ml (p<0.01)  - Sum 3SKF: r= 0.85, SEE= 0.008 g/ml (p<0.01)  Cross-validation of these equations:  - All equations: r=0.80, SEE<0.0100 g/ml (p<0.01) | All equations (composed by Sum 3SKF, Sum 4SKF or Sum 7 SKF) showed very high validity to assess %BF. |
| Johnson et al. 2011^32^ | Females=332  Males=291  Healthy  BMI=25.7  European-American | (20-50)  ±34.9 | - HC, WC  - %BF equation:  1. BAI= HC (cm)/height (m)1.5−18  2. BAIFels= 1.26 × (HC (cm)/height (m)1.4)−32.85  3. BMI | DXA | Pearson correlation coefficient (r), bias correction factor (c), LCCC ((Pc(SD)) | Correlations of BAI equation vs %BF:  - Females: r= 0.75, c= 0.89 (p<0.05),  LCCC ((Pc(SD))= 0.676 (0.026)  - Males: r= 0.72, c= 0.77 (p<0.05),  LCCC ((Pc(SD))= 0.559 (0.028)  Correlation of BAIFels equation vs %BF:  - Females: r= 0.76, c= 0.98 (p<0.05), LCCC ((Pc(SD))= 0.748 (0.024)  - Males: r= 0.73, c= 0.91 (p<0.05),  LCCC ((Pc(SD))= 0.668 (0.028)  Correlation of BMI equation vs %BF:  - Females: r= 0.74, c= 0.54 (p<0.05), LCCC ((Pc(SD))= 0.406 (0.026)  - Males: r= 0.72, c= 0.76 (p<0.05),  LCCC ((Pc(SD))= 0.561 (0.030) | BAI and BMI equations showed high validity to assess %BF. |
| Ketel et al. 2007^33^ | Females=200  Males=176  Healthy  BMI=24.0  Caucasian | (36-37)  36.1±0.7  (35-36)  36.0±0.8 | - HC, WC, WHR  - SKF: subscapular, suprailiac  - BMI  - Central fat mass equations:  1. Females:  a) 18.53 + 0.20 WC + 0.51BMI  b) 12.90 + 0.13 WC + 0.36BMI + 0.23 subscapular SKF  c) 17.67 + 0.13WC + 0.16 BMI + 0.11HC + 0.20 subscapular SKF  2. Males:  a) 33.00+0.34WC +0.48BMI  b) 25.40 + 0.25WC + 0.32 BMI + 0.31 subscapular SKF  c) 22.80 + 0.22WC + 0.31 BMI + 0.16 (subscapular + suprailiac SKF) | DXA | Pearson correlation coefficient (r), Linear regression | Correlation of body composition measures vs %BF:  Females:  - HC, r= 0.78 (p<0.001)  - WC, r= 0.82 (p<0.001)  - WHR, r= 0.21 (p>0.05)  - BMI, r= 0.82 (p<0.001)  - Subscapular+suprailiac, r= 0.82 (p<0.001)  - Subscapular, r= 0.82 (p<0.001)  - Suprailiac, r= 0.72 (p<0.001)  Males:  - HC, r= 0.81 (p<0.001)  - WC, r= 0.85 (p<0.001)  - WHR, r= 0.27 (p<0.001)  - BMI, r= 0.81 (p<0.001)  - Subscapular+suprailiac, r= 0.84 (p<0.001)  - Subscapular, r= 0.80 (p<0.001)  - Suprailiac, r= 0.78 (p<0.001)  Regression analysis of equations vs %BF:  Females (all, p<0.05):  a) R^2^= 0.75  b) R^2^= 0.79  c) R^2^= 0.82  Males (all, p<0.05):  a) R^2^= 0.77  b) R^2^= 0.82  c) R^2^= 0.83 | All measures (except for WHR) showed high validity to assess central fat mass, compared with DXA.  All equations (based on WC + BMI + SKF) showed high validity to assess central fat mass. |
| Lahav et al. 2018^34^ | Females=113  Males=111  Healthy  BMI=27.2  *Race-ethnicity not described* | (20-62)  38.3±10.5  (21-60)  38.7±9.8 | - AbC, NC, WC  - SKF: biceps, triceps, subscapular, suprailiac  - %BF equations:  1. Females, %BFcal= 19.197(±7.439)-0.239(±0.035) x height +0.808(±0.29) x AbC -0.518(±0.128) x NC  2. Males, %BFcal= 10.111(±6.541)-0.239(±0.035) x height +0.808(±0.029) x AbC-0.518(±0.128) x NC  - SKF, %BFSKF by Durnin/ Womersley (DW Sum 4SKF) | DXA | Linear regression, SEE, Bland-Altman method | Regression analysis of %BFcal vs %BF:  - Females: R^2^= 0.65, SEE= 4.85 (p<0.05)  - Males: R^2^= 0.78, SEE= 4.66 (p<0.05)  Agreement:  - Females, MD (± 95% LoA)= 0.1 (-8.4 to 8.4)  - Males, MD (± 95% LoA)= 0.0 (-7.8 to 7.8)  Accuracy of %BFcal vs %BFSKF:  - Females, %BFcal (MD)= 0.24; %BFSKF (MD)= -3.01  - Males, %BFcal (MD)= -0.93; %BFSKF (MD)= -2.48 | Both equations (based on AbC + NC) showed high validity to assess %BF.  DW Sum 4SKF equation was less accurate for prediction of %BF than %BFcal. |
| Lanham et al. 2001^35^ | Females=40  Healthy  BMI=21.7  Chinese Australian | (18-45)  32.5±8.0 | - SKF: biceps, subscapular, suprailiac, triceps  - %BF equations:  1. Durnin/Womersley (DW Sum 4SKF)  2. BIA | D_2_O | Pearson correlation coefficient (r), ANOVA, Bland-Altman method | Correlation of equations vs %BF:  - DW Sum 4SKF: r= 0.71, t= -9.53 (p<0.001)  - BIA equation: r= 0.76, t= 10.58 (p<0.001)  Agreement:  - DW, MD (SD): -7.6 (4.5)  - BIA, MD (SD): -6.7 (4.1) | Both equations, DW Sum 4SKF and BIA, showed high validity to assess %BF.  However, both equations suggest an overestimation of body fat levels for leaner individuals and under estimation for overfat individuals. |
| Lee et al. 2017^36^ | Females=2015  Males=2292  Healthy  BMI=26.1  White, Black, Mexican American, Hispanic, Other | (≥18)  45.3±24.2  42.7±22.4 | - AC, CC, TC, WC  - SKF: subscapular, triceps  - BMI  - %BF equations:  1. Based on: Age, height and weight.  2. Based on: Age, height, weight and WC  3. Based on: Age, height, weight, WC, AC, CC and TC  4. Based on: Age, height, weight, WC, AC, CC, TC, subscapular and triceps SKF | DXA | Linear regression, SEE | Regression analysis of equations vs %BF:  Females:  1) R^2^= 0.66, SEE= 3.87 (p<0.01)  2) R^2^= 0.66, SEE= 3.85 (p<0.01)  3) R^2^= 0.69, SEE= 3.69 (p<0.01)  4) R^2^= 0.74, SEE= 3.36 (p<0.01)  Males:  1) R^2^= 0.62, SEE= 3.57 (p=0.16)  2) R^2^= 0.74, SEE= 3.01 (p<0.01)  3) R^2^= 0.74, SEE= 3.31 (p<0.01)  4) R^2^= 0.80, SEE= 2.66 (p<0.01) | All the equations (based on age, height, weight + WC, AC, CC, TC + subscapular and triceps SKF) showed high validity to assess %BF. |
| Lukaski et al. 1986^37^ | Females=67  Males=47  Healthy  %BF=25.1 females; 16.2 males  *Race-ethnicity not described* | (19-50)  27±7 | - SKF: biceps, scapula, suprailiac, triceps  - BIA  - %BF equation (from BD):  1. Brozek  - SKF by Durnin/ Womersley (DW Sum 4SKF) | UWW | Pearson correlation coefficient (r), regression analysis, SEE | Correlation of BIA vs UWW:  - Females, r= 0.95 (p<0.05)  - Males, r= 0.98 (p<0.05)  Regression analysis of %BF:  - R^2^= 0.98, SEE= 2.20 (p<0.001)  Accuracy between body composition methods and %BF:  - DW Sum 4SFK, r= 0.88, SEE= 3.89 (p<0.05)  - BIA, r= 0.93, SEE= 0.66 (p<0.05) | BIA showed very high validity to assess %BF, compared with UWW.  DW Sum 4SFK showed very high to assess %BF, compared with UWW. |
| Noppa et al. 1979^38^ | Females=153  BMI=24.6  Swedish | (44-66) | - AC, GlC, WC  - SKF: subscapular, triceps  - BMI  - BF equations:  Based on GlC and Sum subscapular and triceps SKF | K40, Isotope dilution | Pearson correlation coefficient (r), multiple regression analysis, SEE | Correlation of body composition measures vs BF (all, p<0.05):  - AC: r= 0.77  - GlC: r= 0.86  - WC: r= 0.84  - Sum 2SKF: r= 0.77  - Triceps SKF: r= 0.74  - Subscapular SKF: r= 0.71  - BMI: r= 0.88  Regression analysis of equations (all, p<0.05):  - 44 years, R^2^= 0.95, SEE= 2.77; 52 years, R^2^= 0.93, SEE= 2.37; 56 years, R^2^= 0.95, SEE= 2.40; 60 years, R^2^= 0.90, SEE= 2.92; 66 years, R^2^= 0.91, SEE= 3.78; all sample, R^2^= 0.92, SEE= 2.96 | All body composition measures showed high validity to predict BF, compared with K40/Isotope dilution.  All equations (based on GlC + subscapular and triceps SKF) showed very high validity to predict BF. |
| Pasco et al. 2012^39^ | Females=1076  Males=1467  Healthy  BMI, 3 groups: <25.0, 25.0-29.9, ≥30)  White | (20-96) | - BMI | DXA | Multiple regression analysis, SEE, CI | Correlation of BMI vs BF (kg):  Females:  - BMI 25 kg/m^2^: BF= 24.0 kg (95%CI 23.7–24.3); BMI 30 kg/m^2^: BF= 33.5 kg (95%CI 33.2–33.8)  Males:  - BMI 25.0 kg/m^2^: BF= 17.7 kg (95%CI 17.5–17.9); BMI 30 kg/m^2^: BF= 27.2 kg (95%CI 27.0–27.5)  Correlation of BMI vs %BF:  Females:  - BMI 25 kg/m^2^: %BF= 36.7% (95%CI  36.4–37.0); BMI 30 kg/m^2^: %BF= 44.2% (95%CI 43.8–44.6)  Males:  - BMI 25.0 kg/m^2^: %BF= 22.7% (95%CI 22.4–23.0); BMI 30 kg/m^2^: %BF= 29.9% (95%CI 29.6–30.2)  Prediction equation:  - Sex+age+BMI: R^2^= 0.82, SEE= 4.07 (p<0.001) | BMI equation showed very high to assess total BF, compared with DXA.  Sex and age should be considered when BMI is used to indicate %BF. |
| Pollock et al. 1975^40^ | Females=143  Healthy  %BF=24.8 young; 29.8 middle-aged  *Race-ethnicity not described*  Two age groups: young (18-22) and middle-aged (33-50) | (18-50)  20.2±1.2  44.7±5.7 | - AbC, AC, AkC, CC, ChC, FC, GlC, ShC, TC, WC, WrC  - Biacromial D, bi-iliac D, bitrochanter D, ChD, KD, WrD  - Cup size  - SKF: abdomen, axilla, chest, subscapula, suprailium, thigh, triceps  - %BF equation (from BD):  1. Brozek  2. Siri | UWW | Pearson correlation coefficient (r), regression analysis, SEE | Correlation of BMI vs %BF (from BD):  Young (all, p<0.05):  - Circumferences, r= 0.19-0.58  - Diameters, r= 0.03-0.65  - SKF, r= 0.50-0.73  Middle-aged (all, p<0.05):  - Circumferences, r= 0.40-0.83  - Diameters, r= 0.18-0.71  - SKF, r= 0.49-0.82  Best prediction equations:  - Young:  SKF+circumferences+diameters, R^2^= 0.84, SEE= 0.008 (p<0.05)  - Middle-aged:  SKF+circumferences+diameters+cup size, R^2^= 0.91, SEE= 0.006 (p<0.05) | In young women, thigh SKF showed high validity to validity to assess total %BF (from BD), compared with UWW.  In middle-aged women, all SKF (except for thigh and knee), ChC, AbC, WC, GlC, ChD, bi-iliac D, bitrochanter D, and cup size showed high validity to validity to assess total %BF (from BD), compared with UWW.  Equations combining SKF + circumferences + diameters for young women and SKF + circumferences + diameters + cup size for middle-aged women, best predicted %BF. |
| Pollock et al. 1976^41^ | Males=84  Healthy  %BF=13.4 young; 24.7 middle-aged  *Race-ethnicity not described*  Two age groups: young (18-22) and middle-aged (40-55) | (18-55)  19.7±1.5  44.9±4.8 | - AbC, AC, AkC, CC, ChC, FC, GlC, ShC, TC, WC, WrC  - Biacromial D, bi-iliac D, bitrochanter D, ChD, KD, WrD  - SKF: abdomen, axilla, chest, subscapula, suprailium, thigh, triceps  - %BF equation (from BD):  1. Brozek  2. Siri | UWW | Pearson correlation coefficient (r), regression analysis, SEE | Correlation of BMI vs %BF (from BD by UWW):  Young (all, p<0.05):  - Circumferences, r= 0.02-0.59  - Diameters, r= 0.12-0.47  - SKF, r= 0.73-0.77  Middle-aged (all, p<0.05):  - Circumferences, r= 0.15-0.70  - Diameters, r= 0.18-0.71  - SKF, r= 0.05-0.67  Best prediction equations:  - Young:  SKF+circumferences+diameters, R^2^= 0.88, SEE= 0.007 (p<0.05)  - Middle-aged:  SKF+circumferences, R^2^= 0.84, SEE= 0.007 (p<0.05) | In both, young and middle-aged men, SKF showed high validity to validity to assess total %BF (from BD), compared with UWW.  In middle-aged men, AbC, GlC, TC, WC and bi-ilium D also showed high validity to validity to assess total %BF (from BD), compared with UWW.  Equations combining SKF + circumferences + diameters in young men; and SKF + circumferences in middle-aged men, best predicted %BF. |
| Pongchaiyakul et al. 2005^42^ | Females=130  Males=83  Healthy  BMI=23.3  Rural Thai | (20-84) | - HC, WC  - SKF: biceps, subscapular, suprailiac, triceps  - %BF equations:  1. Females= 0.417 x HC +0.172 x suprailiac SKF +0.46 x BMI -23.748  2. Males= 0.417 x subscapular SKF+ 0.621 x BMI -0.276 x bíceps SKF  +0.166 x WC -18.466 | DXA | Linear regression, Bland-Altman method | Regression analysis of body composition measures vs %BF:  - Females, R^2^= 0.68 (p<0.01)  - Males, R^2^= 0.68 (p<0.01)  Agreement:  - Females, MD (± 95% LoA)= -0.1% (-9.0 to 9.0)  - Males, MD (± 95% LoA)= 0.03% (-8.2 to 8.3) | Both equations showed high validity to assess %BF.  In females, the equation was based on HC + suprailiac SKF + BMI.  In males, the equation was based on WC + subscapular SKF + BMI. |
| Simoes et al. 2015^43^ | Females=205  Males=186  Healthy  %BF=37 females; 27 males  Portuguese | (24-64)  47.4±11.1  48.8±10.8 | - AbC, AC, WC, HC, mid-thigh C  - SKF: abdominal, biceps, subscapular, suprailiacal, thigh, triceps  - %BF equations:  1. Model 1:  Females: -23.14+0.25 WC+0.28 HC+0.72 AC -0.27 Weight+0.12 Age.  Males: 9.61+0.29 WC+0.19 HC -0.16 Height.  2. Model 2:  Females: 36.12+0.16 thigh SKF+0.14 subscapularSKF+0.28 tricepsSKF -0.18 Height+0.17 Weight+0.10 Age.  Males: 32.83+0.11 abdominalSKF+0.17 suprailiacSKF+0.27 tricepsSKF – 0.16 Height+0.11 Weight+0.08 Age.  3. Model 3:  Females: -8.00+0.20 WC+0.21 HC+0.26 tricepsSKF +0.13 subscapularSKF+0.15 thighSKF –0.16 Weight +0.11 Age.  Males: 20.91+0.21 WC+0.22 suprailiacSKF+0.23 tricepsSKF -0.12 Height. | DXA | Multiple linear regression, SEE, RRSE | Regression analysis of equations vs %BF:  - Model 1:  Females: R^2^= 0.66, SEE= 4.0 (p<0.05)  Males: R^2^= 0.57, SEE= 3.8 (p<0.05)  - Model 2:  Females: R^2^= 0.69, SEE= 3.8 (p<0.05)  Males: R^2^= 0.69, SEE= 3.2 (p<0.05)  - Model 3:  Females: R^2^= 0.71, SEE= 3.7 (p<0.05)  Males: R^2^= 0.68, SEE= 3.2 (p<0.05)  Agreement:  Females: RRSE (SD)= 0.51 (0.14)  Males: RRSE (SD)= 0.59 (0.11) | All model equations (based on circumferences and SKF) showed high validity to predict %BF. |
| Smith & Boyce 1977^44^ | Females=61  Healthy  %BF=25.1  *Race-ethnicity not described* | (25-37)  29.1±3.2 | - AbC, AC, AkC, CC, ChC, FC, HC, ShC, TC  - Biacromial D, Bi-iliac D, Bi-trochanteric D, ChD, KD, WrD  - SKF: abdominal, knee, midaxillary,  scapula, suprailiac, thigh, triceps  - %BF equation (from BD):  1. Brozek | UWW | Pearson correlation coefficient (r), regression analysis, SEE | Correlation of equations vs %BF (from BD):  - Circumferences: r= 0.82, SEE= 0.008 (p<0.05)  -SKF+circumferences: r= 0.82, SEE= 0.008 (p<0.05)  - SKF+diameters+circumferences: r= 0.82, SEE= 0.008 (p<0.05)  - Heith+weight: r= 0.65, SEE= 0.010 (p<0.05) | All measures, except for height + weight, showed high validity to assess total %BF, compared with UWW.  Equation based on weight + SKF + circumferences best predicted %BF. |
| Steinkamp et al. 1965^45^ | Females=817  Males=1236  Healthy  BF (kg)=19.8  Caucasian/ White, Black (males)  Age-groups: 25-34 and 35-44 Whites, 25-44 Blacks | (25-44) | - AC, AkC, ChC, TC, WC, WrC  - Biacromial D, Bi-iliac D, Chest D  - SKF: abdomen, arm, chest, scapular | K40, Isotope dilution | Pearson correlation coefficient (r), regression analysis, SEE | Correlation of body composition measures vs BF (kg):  - Female, White: r= 0.38-0.88, circumferences; r= 0.41-0.64, diameters; r= 0.80-0.85, SKF in 25-34 years. r= 0.23-0.94, circumferences; r= 0.35-0.74, diameters; r= 0.71-0.88, SKF in 35-44 years (all, p<0.05)  - Male, White: r= 0.61-0.91, circumferences; r= 0.54-0.73, diameters; r= 0.70-0.85, SKF in 25-34 years; r= 0.29-0.85, circumferences. r= 0.23-0.50, diameters; r= 0.34-0.60, SKF in 35-44 years (all, p<0.05)  - Male, Black (25-44 years): r= 0.53-0.87, circumferences; r= 0.30-0.56, diameters; r= 0.56-0.88, SKF (all, p<0.05)  Regression equations:  - R^2^= 0.92-0.98, SEE= 2.044-3.665 (p<0.05) | In White females aged 25-44 years and White males aged 25-34 years, SKF showed high validity to assess total BF.  In Blacks, chest and abdomen SKF also showed high validity to assess total BF.  All prediction equations (based on circumferences + diameters + SKF) showed very high validity to assess total BF, regardless of sex and race. |
| Thomas et al. 1998^46^ | Females=54  Healthy,  BMI=22.2 normal weight, 26.6 overweight, 34.6 obese 1, 46.4 obese 2  *Race-ethnicity not described* | (18-45)  32.5±2.19 | - SKF: biceps, subscapular, suprailiac, triceps  - BIA  - %BF (from BD):  1. Brozek  2. Siri  - SKF by Durnin/ Womersley (DW Sum 4SKF) | MRI | Pearson correlation coefficient (r), Bland-Altman method | Field test correlations vs DXA:  Total sample:  - DW Sum 4SFK: r= 0.88 (p<0.01)  - BIA: r= 0.93 (p<0.01)  Group with normal weight:  - DW Sum 4SFK: r= 0.79 (p<0.01)  - BIA: r= 0.54 (p<0.02)  Group with overweight:  - DW Sum 4SFK: r= 0.59 (p<0.02)  - BIA: r= 0.84 (p<0.01)  Group with obesity 1:  - DW Sum 4SFK: r= 0.28 (p<0.14)  - BIA: r= 0.91 (p<0.01)  Group with obesity 2:  - DW Sum 4SFK: r= 0.28 (p<0.28)  - BIA: r= 0.90 (p<0.01)  Agreement:  - DW Sum 4SFK vs MRI, MD (± 95% LoA)= 13.0 (-2.5 to 28.0)  - BIA vs MRI, MD (± 95% LoA)= 9.8 (2.5 to 19.6) | In the group with normal weight (and total sample), DW Sum 4SKF showed high validity; in the group with overweight, it showed moderate validity; and in the groups with obesity, low validity, to assess %BF, compared with MRI.  BIA showed high validity in the group with overweight, and very high validity in the groups with obesity, to assess %BF, compared with MRI. |
| Tucker et al. 2001^47^ | Males=154  Healthy  BMI=23.8  White | (18-26)  22.2±2.1 | - AC, AbC, CC, ChC, FC, HC, NC, TC, WC  - Diet questions  - Fitness and exercise questions  - %BF equation:  + 0.00267 * HC2 (cm)  + 0.34142 * WC  + 1.47496 * Fitness question [possible  scores 1-7]  - 0.65736 * Weight question [possible  scores 1-6]  - 0.40478 * Exercise question [possible  scores 1-8]  - 0.23323 * Cereal question [possible  scores 1-9]  - 0.10898 * Weight in pounds  - 20.88722 (constant) | UWW | Multiple regression, SEE | Regression analysis of proposed equation vs %BF:  - R^2^= 0.80, SEE= 2.66 (p<0.05) | This equation (based on HC, WC, and diet + exercise variables) showed high validity to assess %BF. |
| Vogel et al. 1988^48^ | Females=1194  Males=319  Healthy  BMI <22.9; 22.9-24.9; 25.0-26.9; >26.9  White, Hispanic, Black | (≥17)  24.1±4.5  30.2±8.9 | - AbC, AC, AkC, CC, ChC, HC, HdC, NC, ShC, TC, WrC  - AkD, Biacromial D, Bi-iliac D, Bi-trochanteric D, ChD, DD, ED, KD, WrD  - %BF equations (from BD by Siri):  1. Females: -35.601 - (0.515 x height)+(0.173 x HC) - (1.574 x FC)-(0.533 x NC)-(0.200 x WC)+(105.328 x Loglo weight)  2. Males: 46.892 - (68.678 x Loglo height)+(76.462 x Loglo (AbC-NC) | UWW | Pearson correlation coefficient (r), multiple regression, SEE | Regression equations:  Females (all, p<0.05):  - Total sample, R^2^= 0.82, SEE= 3.598  - Whites, R^2^= 0.82, SEE= 3.598  - Blacks, R^2^= 0.73, SEE= 4.076  - Hispanics, R^2^= 0.85, SEE= 3.332  Males (all, p<0.05):  - Total sample, R^2^= 0.82, SEE= 4.020  - Whites, R^2^= 0.78, SEE= 3.914  - Blacks, R^2^= 0.82, SEE= 4.012  - Hispanics, R^2^= 0.80, SEE= 4.027  Accuracy %BF equations:  - Females: r= 0.82  - Males: r= 0.82  Cross-validation of equations:  - Females: r= 0.79, SEE= 4.4  - Males: r= 0.89, SEE= 3.7 | These equations showed very high validity to assess %BF in all groups, regardless of ethnicity.  In females, the equations were based on HC + FC + NC + WC.  In males, the equations were based on AbC + NC. |
| Wang et al. 1994^49^ | Females=390  Males=297  Healthy  BMI=15-38  White, Asian | (18-94)  51±19 | - AC, ChC, IC, TC, WC  - SKF: abdomen, biceps, chest, suprailiac, subscapular, thigh, triceps, umbilicus  - BMI  - %BF equations:  - Females, White: based on BMI, age, abdominal, thigh and triceps SKF  - Females, Asian: based on BMI, age, subscapular, suprailiac, thigh and triceps SKF  - Males, White: based on BMI, age, abdominal-umbilicus, subscapular and thigh SKF  - Males, Asian: based on BMI, age, abdominal, thigh and triceps SKF | DXA | Regression analysis, SEE | Regression analysis of BMI vs %BF:  Females (both, p<0.05):  - Whites, R^2^= 0.56, SEE= 5.7  - Asian, R^2^= 0.55, SEE= 4.4  Males (both, p<0.05):  - Whites, R^2^= 0.35, SEE= 5.2  - Asian, R^2^= 0.40, SEE= 4.9  Equations for predicting %BF:  Females (both, p<0.05):  - Whites, R^2^= 0.75, SEE= 4.4  - Asian, R^2^= 0.73, SEE= 3.5  Males (both, p<0.05):  - Whites, R^2^= 0.71, SEE= 3.5  - Asian, R^2^= 0.67, SEE= 3.7 | Equations including age, BMI + SKF showed high validity to assess %BF, compared with DXA in all groups.  BMI alone is less accurate to assess %BF. |
| Wang et al. 1996^50^ | Females=124  Males=81  Healthy  BMI=22.4  Chinese | (18-67)  34.2±11.1  32.1±11.6 | - SKF: biceps, subscapular, suprailiac, triceps  - BIA  - BMI  - %BF (from BD by Siri):  1. SKF by Durnin/ Womersley (DW Sum 4SKF) | UWW | Pearson correlation coefficient (r), Bland-Altman method | Field test correlations vs UWW:  Females:  - DW Sum 4SFK: r= 0.82 (p<0.0001)  Agreement: MD (± 95% LoA)= 0.59 (-14.5 to 10.5)  - BIA: r= 0.83 (p<0.0001)  Agreement: MD (± 95% LoA)= 0.63 (-12.0 to 10.0)  - BMI: r= 0.82 (p<0.0001)  Agreement: MD (± 95% LoA)= 0.66 (-12.5 to 14.8)  Males:  - DW Sum 4SFK: r= 0.70 (p<0.0001)  Agreement: MD (± 95% LoA)= 0.36 (-10.0 to 11.3)  - BIA: r= 0.69 (p<0.0001)  Agreement: MD (± 95% LoA)= 0.39 (-11.0 to 13.5)  - BMI: r= 0.62 (p<0.0001)  Agreement: MD (± 95% LoA)= 0.63 (-10.3 to 14.7) | In females, all field measurements showed high validity to assess %BF, compared with UWW.  In males, BIA and BMI showed moderate validity and DW Sum 4SFK high validity to assess %BF, compared with UWW. |
| Womersley & Durnin 1977^51^ | Females=324  Males=245  Healthy  %BF=23.2-32.6 females; 2.8-23.8 males  *Race-ethnicity not described*  Age groups: 17-19, 20-29, 30-39, 40-49, ≥50 | (17-72) | - SKF: biceps, subscapula, suprailiac, triceps  - %BF equations (from BD):  1. Siri | UWW | Spearman correlation, linear regression, SEE | Correlation of SKF vs %BF:  - Females: r= 0.77 in 17-19 years, 0.83 in 20-29 years, 0.92 in 30-39 years, 0.75 in 40-49 years, 0.85 in ≥50 years (all, p<0.05)  - Males: r= 0.74 in 17-19 years, 0.76 in 20-29 years, 0.70 in 30-39 years, 0.78 in 40-49 years, 0.81 in ≥50 years (all, p<0.05)  Regression analysis of SKF equations vs %BF:  - Females: SEE= 4.70 in 17-19 years, 5.21 in 20-29 years, 3.67 in 30-39 years, 4.40 in 40-49 years, 4.07 in 50-68 years  - Males: SEE= 3.89 in 17-19 years, 3.94 in 20-29 years, 4.12 in 30-39 years, 3.62 in 40-49 years, 4.37 in 50-76 years | In females, SKF showed high to very high validity to assess the %BF, compared with UWW, especially in 30-39 years old.  In males, SKF showed high validity to assess the %BF, compared with UWW, especially in ≥50 years old.  SKF equations are especially accurate in 30-39 years old females and 40-49 years old males. |
| Al-Bachir et al. 2016^52^ | Males=213  Healthy  %BF=*see results*  Syrian | (18-19) | - SKF: triceps  - HC, WC, WHR  - %BF equations:  1. %BF= 0.5221 x WC(cm) -18.569  2. %BF= 0.6442 x HC(cm) – 40.071  3. %BF=39.123 x WHR –10.009  4. %BF=88.041 x WHtR -17.794 | D_2_O | Linear regression, SEE, Bland-Altman method | %BF values:  - WC equation=13.0±5.6  - WC+SKF equation=14.5±8.6  - D_2_O=21.3±6.4  Regression analysis of equations vs %BF:  - WC: R^2^= 0.55, SEE= 4.30 (p<0.01)  - HC: R^2^= 0.62, SEE= 3.98 (p<0.01)  - WHR: R^2^= 0.11, SEE= 6.07 (p<0.01)  - WHtR: R^2^= 0.52, SEE= 4.45 (p<0.01)  Agreement between WC equations vs %BF:  - MD (± 95% LoA)= -5.0 (-12.5 to 1.7) | All the equation but the equation using WHR (low validity) showed high validity to assess the %BF.  Equations based on WC, HC and WHtR may accurately predict %BF in Syrian men. |
| Al-Gindan et al. 2015^53^ | Females=110  Males=94  Healthy  BMI=25.3  Caucasian, African-American, Hispanic, Asian | (18-86)  44.1±16.4  43.3±15.9 | - AC, CC, HC, TC, WC  - %BF equations:  1. Females: 0.789 weight (kg) + 0.0786 age (years) – 0.342 height (cm) + 24.5  2. Males: 0.198 weight + 0.478 WC− 0.147 height −12.8 | MRI | Linear regression, SEE, Bland-Altman method | Regression analysis of equations vs %BF:  Females:  - R^2^= 0.84, SEE= 3.0 (p<0.001)  Males:  - R^2^= 0.80, SEE= 3.7 (p<0.001)  Agreement:  Females:  - MD (± 95% LoA)= 0.20 (-4.9 to 5.2)  Males:  - MD (± 95% LoA)= -1.3 (-8.6 to 6.0) | Equation for females (based on weight, height and age) showed high validity and equation for males (based on weight, height + WC) showed very high validity to assess %BF. |
| Benito et al. 2019^54^ | Females=48  Males=36  Healthy, sedentary  BMI=28.2 (overweight)  *Race-ethnicity not described* | (18-50)  37.3±8.1 | - SKF: biceps, subscapular, suprailiac, triceps  - BIA  - %BF (from BD by Siri)  1. SKF by Durnin/ Womersley (DW Sum 4SKF) | DXA | Pearson correlation coefficient (r), Bland-Altman method | Field test correlations vs DXA:  Females:  - DW Sum 4SKF: r= 0.63 (p<0.01)  - BIA: r= 0.82 (p<0.01)  Males:  - DW Sum 4SKF: r= 0.77 (p<0.01)  - BIA: r= 0.81 (p<0.01)  Agreement:  - SKF vs DXA, MD (± 95% LoA)= 0.13 (-9.5 to 9.8)  - BIA vs DXA, MD (± 95% LoA)= 0.25 (-8.9 to 9.4) | In males, DW Sum 4SKF showed high validity to assess %BF, compared with DXA.  In females, DW Sum 4SKF showed moderate validity to assess %BF, compared with DXA.  In both sexes, BIA showed high validity to assess %BF, compared with DXA. |
| Bergman et al. 2011^55^ | Females=123  Males=97  Healthy  BMI=30.0  African-Americans, Mexican-American | (20-50) | - HC, WC  - BAI: (HC/Height1.5)-18 | DXA | Pearson correlation coefficient (r), LCCC (Pc), Bland-Altman method | Correlation of equations vs %BF:  - r= 0.85 (p<0.001)  Concordance (bias correction factor):  - Pc= 0.95 (p<0.001)  Agreement:  - MD (± 95% LoA)= 0.3 (-10.0 to 11.5) | The BAI equation showed high validity to assess %BF. |
| Bhat et al. 2005^56^ | Males=141  Healthy  BMI=21.9  Indian | (30-50)  39.3±76.2 | - SKF: biceps, subscapular, suprailiac, triceps  - BIA  - %BF (from BD by Siri)  1. SKF by Durnin/ Womersley (DW Sum 4SKF) | D_2_O | Pearson correlation coefficient (r), Bland-Altman method | Field test correlations vs D_2_O:  - DW Sum 4SKF: r= 0.92 (p<0.001)  - BIA: r= 0.92 (p<0.001)  Agreement:  - DW Sum 4SKF vs D_2_O, MD (± 95% LoA)= -1.2 (-8.0 to 5.6)  - BIA vs D_2_O, MD (± 95% LoA)= 1.0 (-4.0 to 6.0) | Both measures, DW Sum 4SKF and BIA, showed very high validity to assess %BF, compared with D_2_O method. |
| Deurenberg et al. 1991^57^ | Females=708  Males=521  Healthy  BMI=21.0-40.9  Age group 21-25, 26-35, 36-45, 46-55, 56-65  *Race-ethnicity not described* | (≥21) | - BMI  - %BF equations (from BD by Siri):  - %BF equation:  1.20 x BMI+0.23 x age - 10.8 x sex - 5.4 | UWW | Pearson correlation coefficient (r), Linear regression, SEE, ANOVA | Correlation of BMI vs %BF:  - Females: r= 0.51 in 21-25 years, 0.89 in 26-35 years, 0.81 in 36-45 years, 0.75 in 46-55 years, 0.50 in 56-65 years (all, p<0.01)  - Males: r= 0.47 in 21-25 years, 0.92 in 26-35 years, 0.74 in 36-45 years, 0.80 in 46-55 years, 0.72 in 56-65 years (all, p<0.01)  Regression analysis of age- and sex-specific BMI equations vs %BF:  - R^2^= 0.79, SEE = 4.1% | In females aged 26-45 years, BMI showed high validity; in females aged 21-25 years and 56-65 years, moderate validity to assess %BF, compared with UWW.  In males ≥26 years, BMI showed high to very high validity; in males aged 21-25 years, moderate validity to assess %BF, compared with UWW.  The age-sex specific BMI equation showed high validity to assess %BF. |
| Eston et al. 1995^58^ | Females=9  Males=47  Healthy  %BF=*see results*  Chinese | (27-33)  30.3±2.0  (24-43)  34.9±4.6 | - SKF: abdominal, biceps, calf, chest, subscapular, suprailiac, thigh, triceps  - %BF equations (from BD by Brozek):  1. Durnin/ Womersley (DW Sum 4SKF)  2. Jackson/Pollock (JP Sum 3SKF), in males  3. Jackson, Pollock and Ward (JPW Sum 3SKF), in females | UWW | Pearson correlation coefficient (r), SEE, ANOVA | %BF values:  - DW Sum 4SKF =20.1±1.8 females; 15.9±0.6 males  - JP Sum 3SKF=11.7±0.6  - JPW Sum 3SKF=18.5±2.0  - UWW=25.9±1.5 females; 17.9±0.6 males  Correlation of equations vs %BF:  - DW Sum 4SKF: r= 0.89, SEE= 2.5 (p<0.01) females; r= 0.73, SEE= 2.9 (p<0.01) males  - JP/JPW Sum 3SKF: r= 0.93, SEE= 2.0 (p<0.01) females; r= 0.67, SEE= 3.1 (p<0.01) males  ANOVA between methods:  - UWW: mean (SD): 20.0 (1.8), p<0.01 females; 15.9 (0.61) p<0.01 males  - DW Sum 4SKF: mean (SD): 25.9 (1.5), p<0.01 females; 17.9 (0.58) p<0.01 males  - JP/JPW Sum 3SKF: mean (SD): 18.5 (2.0) p<0.01 females; 11.7 (0.61) p<0.01 males | The DW Sum 4SKF equation showed high validity to assess %BF. However, it seems to overestimate %BF in males.  JP Sum 3SKF and JPW Sum 3SKF equations showed very high validity to assess %BF in females but moderate in males. |
| Fedewa et al. 2018^59^ | Females=91  Males=97  Healthy  BMI=24.4  Caucasian, African-American, Hispanic | (18-40)  21.8±4.8 | - WC  - BAI  - %BF equations:  1. BMI  2. WC  3. BAI | DXA | Pearson correlation coefficient (r), linear regression, SEE | Correlation of equations vs %BF:  - BMI: r= 0.60 (p<0.001)  - WC: r= 0.72 (p<0.001)  - BAI: r= 0.64 (p<0.001)  Regression analysis of equations:  Females:  - BMI: R^2^= 0.30, SEE= 5.08 (p<0.001)  - WC: R^2^= 0.41, SEE= 4.64 (p<0.001)  - BAI: R^2^= 0.32, SEE= 5.00 (p<0.001)  Males:  - BMI: R^2^= 0.37, SEE= 4.87 (p<0.001)  - WC: R^2^= 0.53, SEE= 4.21 (p<0.001)  - BAI: R^2^= 0.41, SEE= 4.70 (p<0.001) | In females, all equations showed moderate validity to assess %BF.  In males, only WC equation showed high validity to assess %BF. BMI and BAI showed moderate validity to assess %BF.  BAI and WC should be used over BMI alone. |
| Garcia et al. 2005^60^ | Females=71  Males=46  Healthy  BMI=27.8  White (German) | (20-66)  46.7±14.1  49.1±11.9 | - ChC, HC, TC, WC  - SKF: abdominal, biceps, calf, chin, chest, hip, knee, subscapular, thigh, triceps  - Bone breadths: ankle, chest, elbow, knee, wrist  - Chest depth  - %BF equations:  1. SKF by Durnin/ Womersley (DW Sum 4SKF)  2. Females, BFMNew (kg)= -77.538 + {(0.424  x HC) + [8.777 x (log chin + log triceps SKF + log subscapular SKF)] + (3.128 x knee breadth)}  3. Males, BFMNew (kg)= -40.750 + (0.397 x WC) + [6.568 x (log triceps SKF + log subscapular SKF + log abdominal SKF)] | DXA | Pearson correlation coefficient (r), multiple linear regression, RMSE, Bland-Altman method | Correlation of equations vs %BF:  Females:  - r= 0.95 (p<0.001)  - R^2^= 0.70, RMSE= 3.44 kg (p<0.001)  Males:  - r= 0.94 (p<0.001)  - R^2^= 0.74, RMSE= 2.56 kg (p<0.001)  Agreement:  - Females, MD (± 95% LoA)= 0.007 kg (-6.5 to 6.5)  - Males, MD (± 95% LoA)= 0.046 kg (-6.5 to 6.5)  Comparison new equations vs DW Sum 4SKF:  - %BF-DW Sum 4SKF vs %BF-DXA: r= -0.86 (p<0.001)  - %BF-New vs %BF-DXA:  r= -0.25 (p<0.001) | Both equations showed very high validity to assess %BF.  Adding circumferences to the SKF equations provide a more precise prediction of %BF in comparison with classical DW Sum 4SKF equation. |
| Hassager et al. 1986^61^ | Females=130  Males=98  Healthy  *Fat status and race-ethnicity not described* | (20-72) | - Height, weight  - SKF: triceps, subscapular  - %BF: fat mass/body weight x 100 | ADP | Multiple linear regression, SEE, ANOVA | Regression analysis of equations vs %BF:  - Height+weight+age:  Females: r= 0.95, SEE= 3.4% (p<0.05)  Males: r= 0.87, SEE= 3.7% (p<0.05)  - Height+weight+age+Sum 2SKF:  Females: r= 0.80, SEE= 3.4%8 (p<0.05)  Males: r= 0.83, SEE= 3.2% (p<0.05) | Both equations showed high to very high validity to assess %BF.  Including Sum 2SKF has not advantage in predicting %BF. |
| Hicks et al. 2000^62^ | Females=150  Healthy  BMI=25.5  American Indian | (18-60)  34.3±10.4 | - HC, WC  - SKF: abdominal, axillar, chest, subscapular, suprailium, thigh, triceps  - %BF equations (from BD by Siri):  1. Jackson, Pollock and Ward (JPW Sum 7SKF)  2. Jackson, Pollock and Ward (JPW Sum 3SKF)  3. Heyward’s NIR equations  4. Futrex-5000 NIR equation  5. Ethnic-specific equations:  a) SKF, BD= 1.06198316–0.00038496(Sum 3SKF)-0.00020362(age)  b) NIR, BD= Prediction Equation:  1.08275966-0.00090439 (HC) 0.0369861(Sum2ʌOD2)+ 0.0004167(height)+ 0.0000866(FIT index) –0.0001894(age) | UWW | Linear regression, SEE, Bland-Altman method | Cross-validation of existing equations:  - JPW Sum 7SKF: R^2^= 0.69, SEE= 0.008 (p<0.05); MD: 4.0  - JPW Sum 3SKF: R^2^= 0.68, SEE= 0.008 (p<0.05); MD: -2.1  - Heyward’s NIR: R^2^= 0.62, SEE= 0.009 (p<0.05); MD: -7.7  - Futrex-5000 NIR: R^2^= 0.66, SEE= 5.50 (p<0.05); MD: 9.7  Ethnic-specific equations, validation:  - SKF, R^2^= 0.67, SEE= 0.0084 (p<0.05); MD: 0.6  - NIR, R^2^= 0.74, SEE= 0.00798 (p<0.05); MD: 2.5  Ethnic-specific equations, cross-validation:  - SKF, R^2^= 0.77, SEE= 0.0068 (p<0.05)  - NIR, R^2^= 0.72, SEE= 0.0076 (p<0.05) | Ethnic-specific SKF and NIR (including HC) equations showed high validity to assess %BF. These equations should be chosen over the classical JPW SKF and NIR equations for American Indian female population. |
| Jackson et al. 2002^63^ | Females=359  Males=296  Healthy  BMI=27.1  Black, White | (17-65)  35.1±12.6 | - BMI  - %BF equations (from BD by Siri):  1. Gallagher et al.  2. Deurenberg et al.  3. Jackson/Pollock (JP Sum 3SKF), in males  4. Jackson, Pollock and Ward (JPW Sum 3SKF), in females | UWW | Multiple regression analysis, SEE, ANOVA | Regression analysis of BMI vs %BF:  - Females: R^2^= 0.78, SEE= 4.63% (p<0.05)  - Males: R^2^= 0.68, SEE= 4.90% (p<0.05)  Regression equations:  - Model I (log transformed BMI and %BF):  Females: R^2^= 0.78, SEE= 4.7; males: R^2^= 0.67, SEE= 4.9  - Model II (+age):  Females: R^2^= 0.80, SEE= 4.4; males: R^2^= 0.72, SEE= 4.6  - Model III (+race):  Females: R^2^= 0.81, SEE= 4.3; males: R^2^= 0.72, SEE= 4.6  Mean (±SD) between measured and estimated %BF, by BMI and interaction terms:  - Females, interaction BMI <25kg/m2 and race: Black, 2.0±4.1; White -0.8±4.8  - Males, interaction BMI >30kg/m2 and age: <30 years, 3.3±4.3; 30-45 years, -0.1±3.9; ≥45 years, -1.3±3.7  Cross-validation of published equations:  - Females, Black: r= 0.89 (all), SEE= 4.2-4.8 (p<0.05)  - Females, White: r= 0.88 (all), SEE= 4.3-5.4 (p<0.05)  - Males, Black: r= 0.79-0.80, SEE= 4.5-4.9 (p<0.05)  - Males, White: r= 0.85-0.86, SEE= 4.7-4.8 (p<0.05) | BMI showed high validity to assess body fat, compared with UWW.  BMI is dependent of age and sex, and of race in females. |
| Jackson et al. 2008^64^ | Females=706  Males=423  Healthy  BMI=26.8  White, Hispanic, African-American | (17-35)  21.8±3.1 | - SKF: chest, abdominal and thigh (males), triceps, supra-ilium and thigh (females)  - %BF equation:  1. BF%-GEN (%BF from Siri’s two-component percentage fat equation) | DXA | GLM, regression coefficients (r), Bland-Altman method | Regression analysis of BF%-GEN vs BF%-DXA:  - Females: r= 0.85, GLM= 0.88 (p<0.001)  - Males: r= 0.93, GLM= 0.78 (p<0.001)  Agreement:  - Females, MD (± 95% LoA)= -0.17 (-4.5 to 11.4)  - Males, MD (± 95% LoA)= 0.03 (-5.3 to 8.5) | The equation BF%-GEN showed high validity in females and very high validity in males to assess %BF. |
| Kagawa, Byrne & Hills 2008^65^ | Females=121  Males=95  Healthy  BMI=27.3  Australian | (20-64)  44.2±10.9  47.1±10.5 | - AbC, HC, WC, WHtR  - BMI | DXA | Spearman’s correlation coefficients, multiple linear regression, SEE | Correlation of body composition measures vs %BF:  Females:  - WHtR (WC): r= 0.78 (p<0.01)  - WHtR (AbC): r= 0.77 (p<0.01)  - BMI: r= 0.87 (p<0.01)  Males:  - WHtR (WC): r= 0.74 (p<0.01)  - WHtR (AbC): r= 0.82 (p<0.01)  - BMI: r= 0.78 (p<0.01)  Regression equations:  - WHtR (WC): R^2^= 0.69, SEE= 5.39  - WHtR (AbC): R^2^= 0.72, SEE= 5.12  - BMI: R^2^= 0.75, SEE= 4.82 | WHtR and BMI equations showed high validity to assess %BF, compared with DXA.  WHtR calculated with AbC may be better than using WC. |
| Kanellakis et al. 2010^66^ | Females=196  Healthy  BMI=30.7 (overweight/ obese)  Caucasian | (50-76)  62.6±5.9 | - WC, WHR  - SKF: biceps, subscapular, suprailiac, triceps  - Fat mass (FM) equations:  1. BIA= 38.475 + 0.207×weight−0.092×resistance/height2 + 0.291×reactance/height2  2. SKF= −31.913 + 0.333 × WC + 0.840 × BMI + 0.064 × SumSKF | DXA | Multiple linear regression, SEE, Bland-Altman method | Regression analysis of BIA equation vs %BF:  - R^2^= 0.83, SEE= 2.65 (p<0.0001)  Regression analysis of SKF equation vs %BF:  - R^2^= 0.80, SEE= 1.62 (p<0.0001)  Agreement:  - BIA: MD (± 95% LoA)= 0.26 (-2.86 to 3.38)  - SKF: MD (± 95% LoA)= 0.09 (-5.32 to 5.14) | The SKF equation (including WC + BMI) showed high validity and the BIA equation very high validity to assess FM, in postmenopausal females. |
| Kanellakis et al. 2012^67^ | Females=277  Healthy  BMI=29.2  Caucasian | (50-75)  62±6.2 | - HC, WC  - SKF: biceps, triceps  - %BF equations (from BD by Siri and Brozek):  1. %BF Martarelli et al. (BMI)  2. %BF Visser et al. (SKF, Siri equation)  3. %BF Visser et al. (BMI, Siri equation)  4. %BF Visser et al. (SKF, Brozek equation)  5. %BF Visser et al. (BMI, Brozek equation)  6. %BF Lean et al. (WC)  7. %BF Lean et al. (BMI) | DXA | Pearson correlation coefficient (r), ICC, SEE, Bland-Altman method | Correlation of equations vs %BF:  - Model 1: r= 0.71 (p<0.001), SEE= 4.86, ICC= 0.823  - Model 2: r= 0.52 (p<0.001), SEE= 4.81, ICC= 0.651  - Model 3: r= 0.79 (p<0.001), SEE= 3.42, ICC= 0.883  - Model 4: r= 0.52 (p<0.001), SEE= 4.77, ICC= 0.636  - Model 5: r= 0.79 (p<0.001), SEE= 3.38, ICC= 0.878  - Model 6: r= 0.56 (p<0.001), SEE= 5.76, ICC= 0.718  - Model 7: r= 0.66 (p<0.001), SEE= 2.60, ICC= 0.789  Agreement:  - Model 1: MD (± 95% LoA)= 1.96 (-11.68 to 7.76)  - Model 2: MD (± 95% LoA)= 8.49 (-1.13 to 18.11)  - Model 3: MD (± 95% LoA)= 3.56 (-3.29 to 10.41)  - Model 4: MD (± 95% LoA)= 5.77 (-3.77 to 15.34)  - Model 5: MD (± 95% LoA)= 1.21 (-5.56 to 7.98)  - Model 6: MD (± 95% LoA)= 4.29 (-7.23 to 15.81)  - Model 7: MD (± 95% LoA)= 4.43 (-5.96 to 14.82) | Model 1, 3 and 5 (all based on BMI) showed high validity to assess %BF, in postmenopausal females.  Models based on SKF and WC showed moderate validity to assess %BF, in postmenopausal females. |
| Kanellakis et al. 2017^68^ | Females=408  Males=234  Healthy  BMI=25.8  Caucasian | (18-80)  41.3±15.3 | - CC, FC, HC, NC, WC  - SKF: abdominal, biceps, calf, subscapular, suprailiac, thigh, triceps  - BMI  - %BF equations:  1. %BF=-0.615-10.948 x sex + 0.321 x WC x 0.502 x HC x -0.39 x FC -19.768 x height(m)  2. %BF=-27.787 -5.515 x sex -8.419 x height +0.145 x WC +0.270 x HC +7.509 x log of thigh SKF +20.090 x log of sum of SKF (bicep + tricep + suprailiac + subscapular)-0.445 x FC | DXA | Multiple linear regression, ICC, LCCC (Pc), Bland-Altman method | Regression analysis of models vs %BF:  - Model 1: R^2^= 0.85, SEE= 0.185 (p<0.0001), ICC= 0.95, Pc= 0.91  - Model 2: R^2^= 0.92, SEE= 0.208 (p<0.0001), ICC= 0.97, Pc= 0.95  Agreement:  - Model 1: MD (± 95% LoA)= 0.052 (-8.10 to 8.04)  - Model 2: MD (± 95% LoA)= 0.148 (-6.20 to 5.90) | Model 1 (based on WC + HC + FC) showed high validity and Model 2 (based on WC + HC + FC + SKF) very high validity to assess %BF, compared with DXA. |
| Kholi et al. 2009^69^ | Females=103  Males=105  Healthy  BMI equally distributed between normal weight, overweight and obesity  South Asians | (30-65)  45.0±0.8 | - FC, HC, WC  - SKF: biceps, calf, subscapular, suprailiac, triceps  - %BF equations:  1. Females, NEW= -25.26 -17.36 x (height) + 0.44 x (mass) + 0.34 x (HC) + 8.87 x (log-tricepsSKF) + 0.14 x (age)  2. Males, NEW= -15.57 + 0.30 x (weight) + 0.23 x (WC) – 3.38 x (FC) + 8.83 x (log-tricepsSKF) + 6.70 x (log-bicepsSKF) + 0.13 x (age)  3. Durnin/ Womersley (DW Sum 4SKF, BD by Siri) | DXA | Multiple linear regression (r), SEE, PE, RSME, Bland-Altman method | Correlation of new equations vs %BF:  - Females: r= 0.97, SEE= 2.30 (p<0.001), PE= 2.30, RSME= 3.27  - Males: r= 0.94, SEE= 2.80 (p<0.001), PE= 2.80, RSME= 4.25  Agreement:  - New, females, MD (± 95% LoA)= -0.26 (-7.42 to 7.36)  - New, males, MD (± 95% LoA)= -0.36 (6.88 to 6.15)  - DW Sum 4SKF, females, MD (± 95% LoA)= -5.13 (-14.96 to 4.69)  - DW Sum 4SKF, males, MD (± 95% LoA)= -4.05 (-11.75 to 3.65) | Both ethnic-specific equations (based on circumferences + SKF) showed very high validity to assess %BF.  These equations should be chosen over the classical DW Sum 4SKF equations for South Asian population. |
| Lam et al. 2013^70^ | Females=52  Males=53  Healthy  BMI=28.1  Chinese | (>21)  39.3±11.6 | - HC, WC  - BMI  - %BF equation:  BAI= (hip/heightx1.5) - 18 | DXA | Pearson correlation coefficient (r), multiple linear regression, Bland-Altman method | Correlation of BMI vs %BF:  - Females: r= 0.87 (p<0.01)  - Males: r= 0.81 (p<0.01)  Correlations of BAI vs DXA:  - Females: r= 0.82 (p<0.01)  - Males: r= 0.74 (p<0.01)  Regression models of BMI vs DXA-derived adiposity:  - Adjusted for gender: R^2^= 0.76 (p<0.05)  - Adjusted for age and gender: R^2^= 0.75 (p<0.05)  Regression models of BAI vs DXA-derived adiposity:  - Adjusted for gender: R^2^= 0.69  - Adjusted for age and gender: R^2^= 0.69  Agreement:  - BAI: MD (± 95% LoA)= 5.77 (-14.15 to 2.61) | Both, the BAI and BMI equations showed high validity to assess %BF, compared with DXA. However, the BMI equation showed higher validity than the BAI equation to assess %BF. |
| Leahy et al. 2012^71^ | Females=618  Males=518  Healthy  BMI=25.1  Caucasian | (18-81)  39.5±17.0  31.7±14.6 | - AbC, AC, CC, FC, HC, TC  - SKF: abdominal, biceps, chest, forearm, iliac crest, medial calf, midaxilla, subscapular, supraspinal, thigh, triceps  - %BF equations:  1. Females= (age) x 0.1 + (log AbC x 41.8) + (log bicepsSKF x 11.9) + (log subscapularSKF x 5.6) + (log medialcalfSKF x 8.3) – 79.0  2. Males= (age) x 0.3 + (log abdominalSKF x 5.6) - (log subscapularSKF x 6.5) + (log medialcalfSKF x 3.9) + 6.6  3. Durnin/ Womersley (DW Sum 4SKF, BD by Siri) | DXA | Spearman’s Rank correlations, SEE, Bland-Altman method | Correlation of equations vs %BF:  - New, females: rs= 0.92, SEE= 3.0 (p<0.05)  - New, males: rs= 0.95, SEE= 2.5 (p<0.05)  - DW Sum 4SKF, females: rs = 0.86, SEE= 4.1 (p<0.05)  - DW Sum 4SKF, males: rs = 0.92, SEE= 3.4 (p<0.05)  Agreement:  - New, females, MD (± 95% LoA)= 0.1 (-5.7 to 5.9)  - New, males, MD (± 95% LoA)= 0.0 (-4.8 to 4.9)  - DW Sum 4SKF, females, MD (± 95% LoA)= 0.8 (-7.1 to 8.5)  - DW Sum 4SKF, males, MD (± 95% LoA)= 1.0 (-5.3 to 7.4) | These new equations (based on SKF and adding also AbC in females) showed very high validity to assess %BF, compared with DXA.  The DW Sum 4SKF equation showed high to very high validity, but appear to become less accurate as %BF increases. Therefore, the new equations should be chosen. |
| Lean, Han & Deurenberg 1996^72^ | Females=84  Males=63  Healthy  BMI=24.9  White | (17-65)  40±13.5 | - AC, HC, TC, WC, WHR  - Arm span, leg length  - SKF: biceps, subscapular, suprailiac, triceps  - BMI  - %BF:  12 equations combining anthropometrics + age + sex (including DW Sum 4SKF, BD by Siri; JPW Sum 7SKF; JP Sum 7SKF) | UWW | Multiple linear regression, SEE, Bland-Altman method | Correlation of body composition measures vs %BF  - Females: r= -0.54 to -0.79 (all, p<0.001)  - Males= r= -0.32 to -0.88 (all, p<0.001)  Best regression equations:  - Best, females: BMI + triceps-SKF + age: R^2^= 0.79, SEE= 4.0  - Best, males: WC + triceps-SKF + age: R^2^= 0.86, SEE= 3.2  Other published equations:  - DW Sum 4SKF, females: R^2^= 0.81, SEE= 4.6  - DW Sum 4SKF, males: R^2^= 0.81 SEE= 3.8  - JPW Sum 7SKF, females: R^2^= 0.75, SEE= 3.6  - JP Sum 7SKF, males: R^2^= 0.84, SEE= 3.2 | Both new equations (based on BMI + triceps SKF and age in females; WC + triceps SKF and age in males) showed very high validity to assess %BF.  The classical DW Sum 4SKF, JPW Sum 7SKF, and JP Sum 7SKF showed also very high validity to assess %BF.  The new equations require lees time/measures to assess %BF. |
| Lee et al. 2021^73^ | Females=10009  Males=7599  Healthy  BF (kg)=19.0 females; 15.4 males  Korean | (>19)  49.6±16.0  49.1±15.6 | - WC  - BMI  - %BF equations:  1. age, height, weight, and WC  2. age, height, weight, WC and serum creatinine  3. age, height, weight, WC, serum creatinine level and physical activity  4. age, height, weight, WC, serum creatinine level, physical activity, smoking habit and alcohol use | DXA | Linear regression, SEE, ICC, Bland-Altman method | Regression analysis of equation 4 vs %BF:  Females:  - R^2^= 0.74, SEE= 2.69, ICC= 0.95 (p<0.001)  Males:  - R^2^= 0.83, SEE= 2.23, ICC= 0.91 (p<0.001)  Agreement:  - Females, MD (± 95% LoA)= -0.00 (-4.42 to 4.42)  - Males, MD (± 95% LoA)= 0.01 (-5.44 to 5.46) | Equation 4 (based on age, height, weight + WC and other variables) showed very high validity in males and high validity in females to assess %BF. |
| Macias et al. 2007^74^ | Females=82  Males=73  Healthy  BMI=25.9  Mexican | (21-48)  34.3±7.6  33.9±7.3 | - HC, WC, WHR  - BIA  - %BF equation:  0.7374 * (Ht2 /R) + 0.1763 * (body weight) - 0.1773 * (Age) + 0.1198 * (Xc) - 2.4658 | ADP | Multiple linear regression, SEE, PE, Bland-Altman method | New equation: R^2^= 0.97, PE= 2.96 (p<0.05)  Mean±SD values BIA vs %BF:  - Females, BIA= 36.1±6.1; ADP= 36.2±6.5  - Males, BIA= 25.8±5.9; ADP= 25.9±6.9  Agreement:  - MD (± 95% LoA)= -0.87 (-6.56 to 4.82) | This BIA equation showed very high validity to assess %BF. |
| Manios et al. 2012^75^ | Females=276  Healthy  BMI=20.5-42.0  White | (47-79)  62±6.2 | - WC, GlC  - SKF: biceps, suprailiac, subscapular, triceps  - %BF equation:  FM= 0.069 x bicepsSKF +0.553 x weight -14.655 x height + 0.218 x GlC -9.830 | DXA | Multiple linear regression, ICC, SEE, Bland-Altman method | Regression analysis of equation vs %BF:  - R^2^= 0.93, SEE= 2.14, ICC= 0.983 (p<0.0001)  Agreement:  - MD (± 95% LoA)= 0.16 (-4.05 to 4.36) | This equation (based on weight, height + GlC + biceps SKF) showed very high validity to assess %BF, in postmenopausal females. |
| Nickerson et al. 2016^76^ | Females=63  Males=67  Healthy  %BF=*see results*  Caucasian | (18-37)  22±5 | - SKF: abdominal, chest, mid-axilla, suprailium, subscapular, thigh, triceps  - BMI-%BF equations:  1. Jackson et al. (BMIJA)  2. Deurenberg et al. (BMIDE)  3. Gallagher et al. (BMIGA)  4. Zanovec at al. (BMIZA)  5. Womersley and Durnin (BMIWO)  - SKF-%BF equation (from BD by Brozek):  6. Jackson/Pollock (JP Sum 7SKF), in males; Jackson, Pollock and Ward (JPW Sum 7SKF), in females | 4C model:  - UWW  - DXA  - BIA | Pearson correlation coefficient (r), CE, SEE, TE, Bland-Altman method | %BF values:  - BMIJA=23.9 6.9  - BMIDE=23.3±6.2  - BMIGA=23.8±7.5  - BMIZA=21.4±8.9  - BMIWO=24.8±5.9  - JP and JPW Sum 7SKF =16.8±7.1  - 4C=21.6±7.8  Equation correlations vs %BF:  - BMIJA: r= 0.56, SEE= 6.4, TE= 7.3, CE= 2.4 (p<0.001)  - BMIDE: r= 0.67, SEE= 5.8, TE= 6.1, CE= 1.8 (p<0.001)  - BMIGA: r= 0.64, SEE= 5.9, TE= 6.8, CE= 2.2 (p<0.001)  - BMIZA: r= 0.73, SEE= 5.3, TE= 6.2, CE= -0.2 (p=0.745)  - BMIWO: r= 0.66, SEE= 4.4, TE= 6.7, CE= 3.2 (p<0.001)  - JP and JPW Sum 7SKF: r= 0.88, SEE= 3.4, TE= 6.0, CE= -4.8 (p<0.001)  Agreement:  - BMIJA: MD (± 95% LoA)= 1.5 (-12.0 to 17.0)  - BMIDE: MD (± 95% LoA)= 1.5 (-10.0 to 12.0)  - BMIGA: MD (± 95% LoA)= 1.5 (-10.0 to 17.0)  - BMIZA: MD (± 95% LoA)= 0.0 (-12.0 to 11.0)  - BMIWO: MD (± 95% LoA)= 1.5 (-8.0 to 15.0)  - JP and JPW Sum 7SKF: MD (± 95% LoA)= -5.0 (-11.0 to 2.0) | JP and JPW Sum 7SKF equations showed high validity to assess %BF and it is recommended over BMI-based equations (which showed moderate validity). |
| O’Connor et al. 2010^77^ | Females=705  Males=428  Healthy  BMI=26.6  Non-Hispanic White (NHW), Hispanic, African-American (AA) | (17-35)  21.4±3.1 | - SKF:  1. iliac crest, thigh, triceps, for females  2. abdominal, chest, thigh, for males  -BMI  - SKF by: Jackson, Pollock and Ward (JPW Sum 3SKF), in females; Jackson/Pollock (JP Sum 3SKF), in males  - %BF equations:  1. Model Sum 3SKF  2. Model Sum 3SKF+BMI | DXA | Regression coefficients estimates, SE, Bland-Altman method | Regression analysis of equations vs %BF:  - Sum 3SKF: females, EST= 0.169, SE= 4.01% (95% CI= 3.62%-4.36%) (p<0.001); males, EST= 0.195, SE= 3.07% (95% CI= 2.77%-3.40%) (p<0.001)  - Sum 3SKF+BMI: females, EST= -0.0007, SE= 3.64% (95% CI= 3.41%-3.89%) (p<0.001); males, EST= -0.0005, SE= 3.12% (95% CI= 2.82%-3.44%) (p<0.001)  Agreement:  - Females, MD (± 95% LoA)= 1.2 (-1.3 to 1.1)  - Males, MD (± 95% LoA)= 1.4 (-1.5 to 1.2) | These ethnic-specific generalized equations (based on Sum 3SKF + BMI) are valid and provide accurate prediction to assess %BF. |
| Pascale et al. 1956^78^ | Males=88  Healthy  BD (g/cc)=1.1  Caucasian | (17-25)  22.1±1.95 | - SKF: abdominal, chest, subscapular, triceps  - %BF equations (from BD):  1. Brozek | UWW | Pearson correlation coefficient (r), multiple linear regression, SEE | Correlation of SKF vs %BF (from BD):  - Chest: r= -0.79 to -0.83 (p<0.05)  - Abdominal: r= -0.72 to -0.77 (p<0.05)  - Triceps: r= -0.77 (p<0.05)  - Subscapular: r= -0.74 (p<0.05)  Regression equation:  - R^2^= 0.85, SEE= 0.006  Brozek equation:  - R^2^= 0.87, SEE= 0.007 | All SKF showed high validity, to assess %BF, compared with UWW.  Equation including chest and triceps SKF showed high validity, to assess %BF. This equation showed a slightly improvement, compared with those of Brozek (although the SKF included were not the same). |
| Ramirez-Zea et al. 2006^79^ | Females=123  Males=114  Healthy  BMI=23.5  Guatemalan | (18-56)  26.5±6 | - AbC, AC, CC, HC, TC, WC  - SKF: abdominal, subscapular, suprailiac, triceps  - %BF equations (from BD by Siri):  1. Females: 19.420  - (weight x 0.385) - (height x 0.215) - (AbC  x 0.265)  2. Males:  -48.472 -(weight x 0.257) - (AbC  x 0.989) | UWW | Multiple linear regression, RMSE, PE | Regression equations (model-building):  - Females: R^2^= 0.78, RMSE= 3.77  - Males: R^2^= 0.74, RMSE= 3.46  Regression equations (validation):  - Females: R^2^= 0.79, PE= 3.53  - Males: R^2^= 0.77, PE= 3.70 | Equations including AbC showed high validity, to assess %BF in Guatemalan population. |
| Ramos-Jiménez et al. 2018^80^ | Females=142  Males=142  Healthy  BMI=24.9  Mexican | (18-35)  21.5±3.9  22.1±3.4 | - WC  - SKF: abdominal, biceps, iliac crest, subscapular, medial calf, midaxilla, supraspinal, thigh, triceps  - %BF equations (from BD by Siri):  1. Females= 0.20 x WC + 0.25 x supraspina SKF + 0.21 x thigh SKF + 0.25 x midaxila SKF – 0.13 x biceps SKF  2. Males= 26.02 + 0.26 x WC + 0.17 x abdominal SKF + 0.31 x thigh + 0.13 x subscapular SKF – 23.59 x height | DXA | Multiple linear regression, SEE, Bland-Altman method | Regression analysis of equations vs %BF:  - Females: R^2^= 0.98, SEE = 0.04 (p<0.001)  - Males: R^2^= 0.78, SEE = 0.03 (p<0.001)  Agreement:  - Females, MD (± 95% LoA)= 0.1 (-8.0 to 10.5)  - Males, MD (± 95% LoA)= 0.1 (-9.8 to 10.3) | Equations (based on WC + SKF) showed high to very high validity to assess %BF. |
| Rush et al. 1997^81^ | Females=82  Healthy  BMI=30  White (New Zealand European), Polynesian | (18-27)  22±2 | - AbC, HC, WC, WHR  - SKF: biceps, subscapular, suprailiac, triceps  - BMI  - %BF equations (from BD by Brozek):  1. SKF by Durnin/ Womersley (DW Sum 4SKF)  2. White= 0.313 AbC + 0.246 HC - 0.3 13 height + 37.62  3. Polynesians= 0.188 AbC + 0.265 HC -7.916 | Isotope dilution | Pearson correlation coefficient (r), multiple linear regression, SEE, Bland-Altman method | Correlation of DW Sum 4SKF vs %BF:  - Whites: r= -0.47 (p=0.002)  - Polynesian: r= -0.39 (p=0.01)  Regression analysis of body composition measures vs %BF:  - Circumferences: R^2^= 0.87, SEE= 3.65%, for Whites; R^2^= 0.83, SEE= 3.16%, for Polynesian (p<0.05)  - DW Sum 4SKF: R^2^= 0.84, SEE= 3.98%, for Whites; R^2^= 0.72, SEE= 4.01%, for Polynesian (p<0.05)  Best equations:  - White: R^2^= 0.86, SEE= 3.83% (p<0.05)  - Polynesian: R^2^= 0.81, SEE= 3.40% (p<0.05) | DW Sum 4SKF showed high validity, to assess %BF, compared with Isotope dilution, in Polynesian.  Both, new race-specific (based on AbC + HC) and DW Sum 4SKF equations showed very high validity, to assess %BF, compared with Isotope dilution, in Whites and Polynesian. |
| Segheto et al. 2016^82^ | Females=395  Males=311  Healthy  BMI=25.3  Brazilian | (20-59)  36.0±12.8 | - WC, WHR  - SKF: abdomen, iliac crest, triceps, in females; chest, subscapular, triceps, in males  - BAI  - BIA  - BMI  - % BF (from BD by Siri):  1. Jackson, Pollock & Ward, in females (JPW Sum 3SKF)  2. Jackson/ Pollock, in males (JP Sum 3SKF) | DXA | Pearson correlation coefficient (r), Bland-Altman method | Field test correlations vs DXA:  Females:  - WC: r= 0.82 (p<0.001)  - WHR: r= 0.55 (p<0.001)  - JPW Sum 3SKF: r= 0.86 (p<0.001)  - BAI: r= 0.78 (p<0.001)  - BIA: r= 0.83 (p<0.001)  - BMI: r= 0.82 (p<0.001)  Males:  - WC: r =0.83 (p<0.001)  - WHR: r= 0.65 (p<0.001)  - JP Sum 3SKF: r= 0.89 (p<0.001)  - BAI: r= 0.72 (p<0.001)  - BIA: r= 0.75 (p<0.001)  - BMI: r= 0.76 (p<0.001)  Agreement:  - Females: BAI vs DXA, MD (± 95% LoA)= 4.1 (-12.4 to 11.6)  - Males: BAI vs DXA, MD (± 95% LoA)= -1.0 (-10.4 to 12.5) | All the measurements (except for WHR) showed high validity to assess %BF, compared with DXA. |
| Shafer et al. 2010^83^ | Females=68  Males=63  Healthy  BMI=22.6 normal weight, 27.3 overweight, 33.7 obese  *Race-ethnicity not described* | (≥18)  35.6±13.3  44.9±15.2  47.2±12.4 | - SKF: biceps, subscapular, suprailiac, triceps  - %BF (from BD by Siri)  1. Durnin/ Womersley (DW Sum 4SKF) | ADP | Linear regression, RMSE, ANOVA, Bland-Altman method | Regression analysis:  - Group with normal weight: R^2^= 0.07 (p<0.06), RMSE= 0.01  - Group with overweight: R^2^= 0.15 (p<0.009), RMSE= 0.00  - Group with obesity: R^2^= 0.00 (p= 0.93), RSME= 0.01  ANOVA:  - Group with normal weight: mean±SD= -2.57±5.11 (p<0.05)  - Group with overweight: mean±SD= 0.85±4.28 (p<0.05)  - Group with obesity: mean±SD= 6.72±5.39 (p<0.05)  Agreement:  - Group with normal weight, MD (± 95% LoA)= 0.00 (-0.01 to 0.02)  - Group with overweight, MD (± 95% LoA)= 0.00 (-0.02 to 0.02)  - Group with obesity, MD (± 95% LoA)= -0.01 (-0.03 to 0.00) | DW Sum 4SFK showed very low to low validity to assess %BF, compared with ADP. |
| Skoufas et al. 2018^84^ | Females=437  Males=272  Healthy  BMI=25.7  Caucasian | (18-80)  40.4±15.3 | - AbC, FC, NC, WC  - SKF: abdominal, biceps, subscapular, suprailiac, triceps  1. Females equation:  %Abdominal FM=-121.368 - (17.322 x Height(m)) + (76,452 x logAbC(cm)) + (18.341 x logSuprailiac SKF(mm)) + (8802 x logAbdominal SKF(mm))  2. Males equation:  %Abdominal FM=-174.117 - (15.247 x Height (m)) + (104.503 x logAbC(cm)) + (9907 x logSuprailiac SKF(mm)) + (7971 x logAbdominal SKF(mm)) | DXA | Correlation coefficient, ICC, Bland-Altman method | Regression analysis of abdominal fat mass equations vs %BF:  - Females:  R^2^= 0.89 (p<0.001), ICC= 0.940  - Males:  R^2^= 0.94 (p<0.001), ICC= 0.957  Agreement:  - Females, MD (± 95% LoA)= 0.20 (-9.15 to 9.56)  - Males, MD (± 95% LoA)= 0.41 (-8.37 to 9.19) | Equations (based on AbC + SKF) showed high to very high validity, to assess %BF. |
| Slaughter et al. 1988^85^ | Females=32  Males=36  Healthy  %BF=15.8 females; 27.0 males  Black and White | 22.6±2.7 | - SKF: abdominal, anterior suprailiac, biceps, subscapula, medial calf, midaxillary, mid-thigh, suprailiac, triceps  - %BF equations (from BD):  1. Siri | D_2_O, photon absorptiometry, UWW | Regression analysis, SEE | Regression analysis of SKF vs %BF:  - Sum SKF triceps+calf: R^2^= 0.82, SEE= 3.6 (p<0.05)  - Sum SKF triceps +subscapular: R^2^= 0.82, SEE= 3.5 (p<0.05)  Prediction equations:  - Sum SKF triceps+calf: B= 0.61, SEE= 3.8 in females; B= 0.73, SEE= 3.8 in males (p<0.05)  - Sum SKF triceps +subscapular: B= 1.33, SEE= 3.9 in females; B= 1.21, SEE= 3.6 in males (p<0.05) | These equations, based on Sum 2SKF showed very high validity to assess %BF. |
| Stout et al. 1994^86^ | Males=57  Healthy  %BF=*see results*  Caucasian | (18-33)  22±3 | - SKF: abdominal, chest, thigh  - %BF equation (from BD, by Brozek)  1. Jackson/Pollock (JP Sum 3SKF)  2. BIA | UWW | Correlation coefficient (r), CE, TE, SEE | %BD values:  - JP Sum 3SKF=12.6±5.2  - BIA=17.8±4.6  - UWW=15.1±6.2  Correlation of equations vs %BF:  - JP Sum 3SKF, r= 0.90 (p=0.05), CE= -2.5, TE= 3.6, SEE= 2.7  - BIA, r= 0.74 (p=0.05), CE= 2.8, TE= 5.0, SEE= 4.2 | The JP Sum 3SKF equation and BIA showed very high validity to assess %BF. |
| Sun et al. 2005^87^ | Females=491  Males=100  Healthy  BMI=26.3  Canadian | (19-60)  42.1±10.3 | - WHtR  - BMI  - BIA | DXA | Pearson correlation coefficient (r), ANOVA, Bland-Altman method | Correlation of BIA vs %BF:  - Whole group: r= 0.88 (p<0.05)  - Females: r= 0.85 (p<0.05)  - Males: r= 0.78 (p<0.05)  Agreement BIA vs %BF:  - All sample: MD (± 95% LoA)=  -1.83±4.10%  - Females: MD (± 95% LoA)=  -1.77±4.00%  - Males: MD (± 95% LoA)=  -2.16±4.56%  ANOVA BIA vs %BF:  - All sample: %BF<20 (MD±SD)= 3.56% (p<0.01); %BF 20-30 (MD±SD)= 0.50% (p<0.01); %BF>30 (MD±SD)= 2.65% (p<0.01)  - Females: %BF<25 (MD±SD)= 4.40% (p<0.01); %BF 25-33 (MD±SD)= 0.50% (p<0.01); %BF>33 (MD±SD)= 2.71% (p<0.01)  - Males: %BF<15 (MD±SD)= 3.03% (p<0.01); %BF 15-25 (MD±SD)= 0.5% (p<0.01); %BF>25 (MD±SD)= 4.32% (p<0.01) | BIA showed high validity to assess %BF, compared with DXA.  BIA overestimates %BF in lean adults and underestimates %BF in obese adults. |
| Wattanapenpaiboon et al. 1998^88^ | Females=130  Males=66  Healthy  BMI=25.4 (entire sample) non-obese, obese  Anglo-Celtic Australian | (26-86)  56.5±14.1 | - SKF: biceps, subscapular, suprailiac, triceps  - BIA:  1. Manufacturer  2. Lukaski 1986  3. Segal et al. 1988  - %BF:  1. SKF by Durnin/ Womersley (DW Sum 4SKF) | DXA | Spearman's rank correlations, ANOVA, Bland-Altman method | Field test correlations vs DXA:  - DW Sum 4SKF: rs= 0.44 (p<0.0001)  - BIA: rs= 0.56 (p<0.0001)  ANOVA:  Females:  - DW Sum 4SKF= 37.4 vs 34.2% (p<0.0001)  - BIA= 35.1 vs 37.4% (p<0.01)  Males:  - DW Sum 4SKF= 24.6 vs 24.7% (p>0.05)  - BIA= 28.2 vs 24.6 % (p<0.001)  Agreement:  Females:  - DW Sum 4SKF vs DXA, MD (± 95% LoA)= 3.0 (-6.0 to 12.0)  - BIA vs DXA, MD (± 95% LoA)= 3.9 (-5.0 to 9.6)  Males:  - DW Sum 4SKF vs DXA, MD (± 95% LoA)= -0.6 (-9.8 to 7.3)  - BIA vs DXA, MD (± 95% LoA)= -0.6 (-15.0 to 14.8) | BIA showed moderate validity to assess %BF (using the Segal et al. 1988 equation), compared with DXA.  DW Sum 4SKF showed low validity to assess %BF, compared with DXA. |
| Yao et al. 2002^89^ | Females=38  Males=33  Healthy  BMI=24.9  Chinese | (35-49)  42.8±0.6  43.1±0.7 | - SKF: abdominal, biceps, mid-thigh, subscapular, suprailiac, triceps  - BMI  - %BF equations:  1. Durnin/ Womersley (DW Sum 4SKF), BD by Siri and by Brozek  2. Wang et al. | D_2_O | Pearson correlation coefficient (r), regression analysis, SEE, Bland-Altman method | Regression analysis of equations vs %BF:  - DW Sum 4SKF, Siri: R^2^= 0.88, SEE= 2.71 (p<0.001)  - DW Sum 4SKF, Brozek et al.: R^2^= 0.88, SEE= 2.71 (p<0.001)  - Wang et al.: R^2^= 0.87, SEE= 2.88 (p<0.001)  Agreement:  - DW Sum 4SKF, Siri: MD (± 95% LoA)= 0.23 (-4.4 to 6.6)  - DW Sum 4SKF, Brozek et al.: MD (± 95% LoA)= 0.00 (-5.5 to 5.3)  - Wang et al.: MD (± 95% LoA)= 0.70 (-8.1 to 7.9) | All equations showed very high validity to assess %BF. |
| Zanovec et al. 2009^90^ | Females=145  Males=133  Healthy  BMI=17-37  Black, White | (18-24)  20.4±1.5 | - BMI  - Physical activity levels | DXA | Pearson correlation coefficients (r), multiple linear regression analysis, RMSE, Bland-Altman method | Correlation of BMI vs %BF:  - Females, White: r= 0.77 (p<0.001)  - Females, Black: r= 0.90 (p<0.001)  - Males, White: r= 0.71 (p<0.001)  - Males, Black: r= 0.87 (p<0.001)  Regression analysis:  - Sex+BMI+race: R^2^= 0.81, RMSE= 4.07  - Sex+BMI+race+physical activity: R^2^= 0.83, RMSE= 3.87  Agreement (sex+BMI+race):  MD (± 95% LoA)= 0.0 (-8.09, 8.09) | BMI equations showed very high validity to assess %BF.  Including physical activity implies greater accuracy. |

AbC, abdominal circumference; AC, arm circumference; AD, arm diameter; ADP, air-displacement plethysmography; AkC, ankle circumference; AkD, ankle diameter; BAI,

body adiposity index; BD, body density; BIA, bioelectrical impedance analysis; BMI, body mass index; C, circumference; CC, calf circumference; ChC, chest circumference; ChD, chest diameter; D, diameter; DD, deltoid diameter; DXA, dual energy x-ray absorptiometry; D_2_O, deuterium oxide; ED, elbow diameter; FC, forearm circumference; FMM, fat free mass; GlC, gluteal circumference; HC, hip circumference; HdC, head circumference; ^3^H_2_O, tritium space; IC, iliac circumference; KC, knee circumference; KD, knee diameter; K^40^, Potassium-40; MRI, magnetic resonance imaging; NC, neck circumference; ShC, shoulder circumference; SKF, skinfolds; TC, thigh circumference; UWW, under water weighing; WC, waist circumference; WHR, waist to hip ratio; WHtR, waist to height ratio; WrC, wrist circumference; WrD, wrist diameter; %BF, body fat percentage.

ANOVA, indicates analysis of variance; CE, constant error; CI, coefficient of interval; CV_RSME,_ CV of the root-mean-square error; EST, regression coefficients estimates; GLM, general linear models; ICC, intra-class correlation coefficient; LCCC, Lin’s concordance coefficient; LoA, limits of agreement; ME, mean error; MD, mean difference; MSD, mean signed difference; PE, pure error; RMSE, root mean square error; RRSE, root relative square error; SD, standard deviation; SE, standard error; SEE, standard error estimate; TE, total error.

**References**

1. Erselcan T, Candan F, Saruhan S, Ayca T. Comparison of body composition analysis methods in clinical routine. *Annals of Nutrition and Metabolism*. Sep-Dec 2000;44(5-6):243-248. doi:10.1159/000046691

2. Hodgdon JA, Beckett MB. *Prediction of percent body fat for US Navy women from body circumferences and height*. 1984.

3. Sloan A, Burt J, Blyth C. Estimation of body fat in young women. *Journal of applied physiology*. 1962;17(6):967-970.

4. Durnin J, Rahaman MM. The assessment of the amount of fat in the human body from measurements of skinfold thickness. *British journal of Nutrition*. 1967;21(3):681-689.

5. Eston RG, Rowlands AV, Charlesworth S, Davies A, Hoppitt T. Prediction of DXA-determined whole body fat from skinfolds: importance of including skinfolds from the thigh and calf in young, healthy men and women. *European Journal of Clinical Nutrition*. May 2005;59(5):695-702. doi:10.1038/sj.ejcn.1602131

6. Friedl KE, Vogel JA. Validity of percent body fat predicted from circumferences: classification of men for weight control regulations. *Mil Med*. Mar 1997;162(3):194-200.

7. Haisman M. The assessment of body fat content in young men from measurements of body density and skinfold thickness. *Human biology*. 1970:679-688.

8. Katch FI, McArdle WD. Prediction of body density from simple anthropometric measurements in college-age men and women. *Human biology*. 1973:445-455.

9. Katch FI, McArdle WD. VALIDITY OF BODY COMPOSITION PREDICTION EQUATIONS FOR COLLEGE MEN AND WOMEN. *American Journal of Clinical Nutrition*. 1975 1975;28(2):105-109.

10. Wilmore JH, Behnke AR. An anthropometric estimation of body density and lean body weight in young men. *Journal of Applied Physiology*. 1969;27(1):25-31.

11. Wilmore JH, Behnke AR. An anthropometric estimation of body density and lean body weight in young women. *The American journal of clinical nutrition*. 1970;23(3):267-274.

12. Temple D, Denis R, Walsh MC, Dicker P, Byrne AT. Comparison of anthropometric-based equations for estimation of body fat percentage in a normal-weight and overweight female cohort: validation via air-displacement plethysmography. *Public Health Nutr*. Feb 2015;18(3):446-52. doi:10.1017/s1368980014000597

13. Aandstad A, Holtberget K, Hageberg R, Holme I, Anderssen SA. Validity and reliability of bioelectrical impedance analysis and skinfold thickness in predicting body fat in military Pearsonnel. *Mil Med*. Feb 2014;179(2):208-17. doi:10.7205/milmed-d-12-00545

14. Aristizabal JC, Restrepo MT, Amalia L. Validation by hydrodensitometry of skinfold thickness equations used for female body composition assessment. *Biomedica*. Sep 2008;28(3):404-13. Validacion por hidrodensitometria de ecuaciones de pliegues cutaneos utilizadas para estimar la composicion corporal en mujeres.

15. Aristizabal JC, Estrada-Restrepo A, García AG. Desarrollo y validación de ecuaciones antropométricas para estimar la composición corporal en mujeres adultas. *Revista Colombia Médica*. 2018;49(2):154-159.

16. Balas-Nakash M, Legorreta-Legorreta J, Rodriguez-Cano A, Aguilera-Perez R, Perichart-Perera O. Validation of body composition estimation equations by bioelectric impedance in postmenopausic women with metabolic syndrome. *Rev Invest Clin*. Nov-Dec 2010;62(6):538-45. Validacion del uso de ecuaciones para estimar la composicion corporal por analisis de impedancia bioelectrica en mujeres postmenopausicas con sindrome metabolico.

17. Ball S, Swan PD, DeSimone R. Comparison of anthropometry to dual energy X-ray absorptiometry: a new prediction equation for women. *Res Q Exerc Sport*. Sep 2004;75(3):248-58. doi:10.1080/02701367.2004.10609158

18. Brožek J, Keys A. The evaluation of leanness-fatness in man: norms and interrelationships. *British Journal of Nutrition*. 1951;5(2):194-206.

19. Cui Z, Truesdale KP, Cai J, Stevens J. Evaluation of anthropometric equations to assess body fat in adults: NHANES 1999-2004. *Med Sci Sports Exerc*. Jun 2014;46(6):1147-58. doi:10.1249/mss.0000000000000213

20. Davidson LE, Wang J, Thornton JC, et al. Predicting Fat Percent by Skinfolds in Racial Groups: Durnin and Womersley Revisited. *Medicine and Science in Sports and Exercise*. Mar 2011;43(3):542-549. doi:10.1249/MSS.0b013e3181ef3f07

21. Demura S, Yamaji S, Goshi F, Kobayashi H, Sato S, Nagasawa Y. The validity and reliability of relative body fat estimates and the construction of new prediction equations for young Japanese adult males. *J Sports Sci*. Feb 2002;20(2):153-64. doi:10.1080/026404102317200864

22. Dioum A, Gartner A, Maire B, Delpeuch F, Wade S. Body composition predicted from skinfolds in African women: a cross-validation study using air-displacement plethysmography and a Black-specific equation. *Br J Nutr*. Jun 2005;93(6):973-9. doi:10.1079/bjn20051426

23. Durnin JV, Womersley J. Body fat assessed from total body density and its estimation from skinfold thickness: measurements on 481 men and women aged from 16 to 72 years. *British journal of nutrition*. 1974;32(1):77-97.

24. Friedl KE, Westphal KA, Marchitelli LJ, Patton JF, Chumlea WC, Guo SS. Evaluation of anthropometric equations to assess body-composition changes in young women. *American Journal of Clinical Nutrition*. Feb 2001;73(2):268-275. doi:doi.org/10.1093/ajcn/73.2.268

25. Gallagher D, Visser M, Sepulveda D, Pierson RN, Harris T, Heymsfield SB. How useful is body mass index for comparison of body fatness across age, sex, and ethnic groups? *American journal of epidemiology*. 1996;143(3):228-239.

26. Gallagher D, Heymsfield SB, Heo M, Jebb SA, Murgatroyd PR, Sakamoto Y. Healthy percentage body fat ranges: an approach for developing guidelines based on body mass index. *The American journal of clinical nutrition*. 2000;72(3):694-701.

27. Goel K, Gupta N, Misra A, et al. Predictive equations for body fat and abdominal fat with DXA and MRI as reference in Asian Indians. *Obesity (Silver Spring)*. Feb 2008;16(2):451-6. doi:10.1038/oby.2007.55

28. Gómez-Ambrosi J, Silva C, Catalán V, et al. Clinical usefulness of a new equation for estimating body fat. *Diabetes Care*. 2012;35(2):383-388.

29. Gomez Campos R, Pacheco Carrillo J, Almonacid Fierro A, Urra Albornoz C, Cossio-Bolanos M. Validation of equations and proposed reference values to estimate fat mass in Chilean university students. *Endocrinol Diabetes Nutr*. Mar 2018;65(3):156-163. Validacion de ecuaciones y propuesta de valores referenciales para estimar la masa grasa de jovenes universitarios chilenos. doi:10.1016/j.endinu.2017.11.008

30. Jackson AS, Pollock ML. Generalized equations for predicting body density of men. *British journal of nutrition*. 1978;40(3):497-504.

31. Jackson AS, Pollock ML, Ward A. Generalized equations for predicting body density of women. *Medicine and science in sports and exercise*. 1980;12(3):175-181.

32. Johnson W, Chumlea WC, Czerwinski SA, Demerath EW. Concordance of the recently published body adiposity index with measured body fat percent in European-American adults. *Obesity (Silver Spring)*. Apr 2012;20(4):900-3. doi:10.1038/oby.2011.346

33. Ketel IJ, Volman MN, Seidell JC, Stehouwer CD, Twisk JW, Lambalk CB. Superiority of skinfold measurements and waist over waist-to-hip ratio for determination of body fat distribution in a population-based cohort of Caucasian Dutch adults. *Eur J Endocrinol*. Jun 2007;156(6):655-61. doi:10.1530/eje-06-0730

34. Lahav Y, Epstein Y, Kedem R, Schermann H. A novel body circumferences-based estimation of percentage body fat. *Br J Nutr*. Mar 2018;119(6):720-725. doi:10.1017/s0007114518000223

35. Lanham DA, Stead MA, Tsang K, Davies PSW. The prediction of body composition in Chinese Australian females. *International Journal of Obesity*. Feb 2001;25(2):286-291. doi:10.1038/sj.ijo.0801473

36. Lee DH, Keum N, Hu FB, et al. Development and validation of anthropometric prediction equations for lean body mass, fat mass and percent fat in adults using the National Health and Nutrition Examination Survey (NHANES) 1999-2006. *Br J Nutr*. Nov 2017;118(10):858-866. doi:10.1017/s0007114517002665

37. Lukaski HC, Bolonchuk WW, Hall CB, Siders WA. Validation of tetrapolar bioelectrical impedance method to assess human body composition. *Journal of applied physiology*. 1986;60(4):1327-1332.

38. Noppa H, Andersson M, Bengtsson C, Bruce Å, Isaksson B. Body composition in middle-aged women with special reference to the correlation between body fat mass and anthropometric data. *The American journal of clinical nutrition*. 1979;32(7):1388-1395.

39. Pasco JA, Nicholson GC, Brennan SL, Kotowicz MA. Prevalence of obesity and the relationship between the body mass index and body fat: cross-sectional, population-based data. *PloS one*. 2012;7(1):e29580.

40. Pollock ML, Laughridge EE, Coleman B, Linnerud A, Jackson A. Prediction of body density in young and middle-aged women. *Journal of applied physiology*. 1975;38(4):745-749.

41. Pollock ML, Hickman T, Kendrick Z, Jackson A, Linnerud A, Dawson G. Prediction of body density in young and middle-aged men. *Journal of Applied Physiology*. 1976;40(3):300-304.

42. Pongchaiyakul C, Kosulwat V, Rojroongwasinkul N, et al. Prediction of percentage body fat in rural thai population using simple anthropometric measurements. *Obes Res*. Apr 2005;13(4):729-38. doi:10.1038/oby.2005.82

43. Simoes M, Severo M, Oliveira A, Ferreira I, Lopes C. Predictive equations for estimating regional body composition: a validation study using DXA as criterion and associations with cardiometabolic risk factors. *Ann Hum Biol*. May 2016;43(3):219-28. doi:10.3109/03014460.2015.1054427

44. Smith DP, Boyce RW. Prediction of body density and lean body weight in females 25 to 37 years old. *The American Journal of Clinical Nutrition*. 1977;30(4):560-564.

45. Steinkamp R, Cohen N, Gaffey W, et al. Measures of body fat and related factors in normal adults—II: A simple clinical method to estimate body fat and lean body mass. *Journal of chronic diseases*. 1965;18(12):1291-1307.

46. Thomas EL, Saeed N, Hajnal JV, et al. Magnetic resonance imaging of total body fat. *Journal of Applied Physiology*. Nov 1998;85(5):1778-1785.

47. Tucker LA, Greenwell SD. Using simple measures to estimate body fat percentage in college men. *American Journal of Health Behavior*. Sep-Oct 2001;25(5):460-467. doi:10.5993/ajhb.25.5.3

48. Vogel JA, Kirkpatrick J, Fitzgerald P, Hodgdon JA, Harman E. *Derivation of anthropometry based body fat equations for the Army's weight control program*. 1988.

49. Wang J, Thornton JC, Russell M, Burastero S, Heymsfield S, Pierson Jr RN. Asians have lower body mass index (BMI) but higher percent body fat than do Whites: comparisons of anthropometric measurements. *The American journal of clinical nutrition*. 1994;60(1):23-28.

50. Wang J, Deurenberg P. The validity of predicted body composition in Chinese adults from anthropometry and bioelectrical impedance in comparison with densitometry. *Br J Nutr*. Aug 1996;76(2):175-82. doi:10.1079/bjn19960023

51. Womersley J, Durnin J. A comparison of the skinfold method with extent of ‘overweight’and various weight-height relationships in the assessment of obesity. *British journal of nutrition*. 1977;38(2):271-284.

52. Al-Bachir M, Ahmad H. VALIDITY OF USING WAIST AND HIP CIRCUMFERENCE MEASUREMENTS TO DETERMINE BODY COMPOSITION OF YOUNG SYRIAN MEN. *J Biosoc Sci*. Sep 2016;48(5):647-57. doi:10.1017/s0021932015000413

53. Al-Gindan YY, Hankey CR, Govan L, Gallagher D, Heymsfield SB, Lean MEJ. Derivation and validation of simple anthropometric equations to predict adipose tissue mass and total fat mass with MRI as the reference method. *British Journal of Nutrition*. Dec 14 2015;114(11):1852-1867. doi:10.1017/s0007114515003670

54. Benito PJ, Gomez-Candela C, Dolores Cabanas M, Szendrei B, Aparecida Castro E, Grp PS. COMPARISON BETWEEN DIFFERENT METHODS FOR MEASURING BODY FAT AFTER A WEIGHT LOSS PROGRAM. *Revista Brasileira De Medicina Do Esporte*. Nov-Dec 2019;25(6):474-479. doi:10.1590/1517-869220192506149743

55. Bergman RN, Stefanovski D, Buchanan TA, et al. A better index of body adiposity. *Obesity (Silver Spring)*. May 2011;19(5):1083-9. doi:10.1038/oby.2011.38

56. Bhat DS, Yajnik CS, Sayyad MG, et al. Body fat measurement in Indian men: comparison of three methods based on a two-compartment model. *Int J Obes (Lond)*. Jul 2005;29(7):842-8. doi:10.1038/sj.ijo.0802953

57. Deurenberg P, Weststrate JA, Seidell JC. Body mass index as a measure of body fatness: age-and sex-specific prediction formulas. *British journal of nutrition*. 1991;65(2):105-114.

58. Eston RG, Fu F, Fung L. VALIDITY OF CONVENTIONAL ANTHROPOMETRIC TECHNIQUES FOR PREDICTING BODY-COMPOSITION IN HEALTHY CHINESE ADULTS. *British Journal of Sports Medicine*. Mar 1995;29(1):52-56. doi:10.1136/bjsm.29.1.52

59. Fedewa MV, Nickerson BS, Esco MR. Associations of body adiposity index, waist circumference, and body mass index in young adults. *Clinical Nutrition*. Apr 2019;38(2):715-720. doi:10.1016/j.clnu.2018.03.014

60. Garcia AL, Wagner K, Hothorn T, Koebnick C, Zunft HJ, Trippo U. Improved prediction of body fat by measuring skinfold thickness, circumferences, and bone breadths. *Obes Res*. Mar 2005;13(3):626-34. doi:10.1038/oby.2005.67

61. Hassager C, Gotfredsen A, Jensen J, Christiansen C. Prediction of body composition by age, height, weight, and skinfold thickness in normal adults. *Metabolism*. 1986;35(12):1081-1084.

62. Hicks VL, Stolarczyk LM, Heyward VH, Baumgartner RN. Validation of near-infrared interactance and skinfold methods for estimating body composition of American Indian women. *Med Sci Sports Exerc*. Feb 2000;32(2):531-9. doi:10.1097/00005768-200002000-00041

63. Jackson AS, Stanforth PR, Gagnon J, et al. The effect of sex, age and race on estimating percentage body fat from body mass index: The Heritage Family Study. *International journal of obesity*. 2002;26(6):789-796.

64. Jackson AS, Ellis KJ, McFarlin BK, Sailors MH, Bray MS. Cross-validation of generalised body composition equations with diverse young men and women: the Training Intervention and Genetics of Exercise Response (TIGER) Study. *Br J Nutr*. Mar 2009;101(6):871-8. doi:10.1017/s0007114508047764

65. Kagawa M, Byrne NM, Hills AP. Comparison of body fat estimation using waist: height ratio using different ‘waist’measurements in Australian adults. *British Journal of Nutrition*. 2008;100(5):1135-1141.

66. Kanellakis S, Kourlaba G, Moschonis G, Vandorou A, Manios Y. Development and validation of two equations estimating body composition for overweight and obese postmenopausal women. *Maturitas*. Jan 2010;65(1):64-8. doi:10.1016/j.maturitas.2009.10.012

67. Kanellakis S, Manios Y. Validation of five simple models estimating body fat in White postmenopausal women: use in clinical practice and research. *Obesity (Silver Spring)*. Jun 2012;20(6):1329-32. doi:10.1038/oby.2011.403

68. Kanellakis S, Skoufas E, Khudokonenko V, et al. Development and Validation of Two Equations Based on Anthropometry, Estimating Body Fat for the Greek Adult Population. *Obesity*. Feb 2017;25(2):408-416. doi:10.1002/oby.21736

69. Kohli S, Gao M, Lear SA. Using simple anthropometric measures to predict body fat in South Asians. *Appl Physiol Nutr Metab*. Feb 2009;34(1):40-8. doi:10.1139/h08-128

70. Lam BC, Lim SC, Wong MT, et al. A method comparison study to validate a novel parameter of obesity, the body adiposity index, in Chinese subjects. *Obesity (Silver Spring)*. Dec 2013;21(12):E634-9. doi:10.1002/oby.20504

71. Leahy S, O'Neill C, Sohun R, Toomey C, Jakeman P. Generalised equations for the prediction of percentage body fat by anthropometry in adult men and women aged 18-81 years. *Br J Nutr*. Feb 28 2013;109(4):678-85. doi:10.1017/s0007114512001870

72. Lean M, Han TS, Deurenberg P. Predicting body composition by densitometry from simple anthropometric measurements. *The American journal of clinical nutrition*. 1996;63(1):4-14.

73. Lee G, Chang J, Hwang SS, Son JS, Park SM. Development and validation of prediction equations for the assessment of muscle or fat mass using anthropometric measurements, serum creatinine level, and lifestyle factors among Korean adults. *Nutrition Research and Practice*. Feb 2021;15(1):95-105. doi:10.4162/nrp.2021.151.95

74. Macias N, Alemán-Mateo H, Esparza-Romero J, Valencia ME. Body fat measurement by bioelectrical impedance and air displacement plethysmography: a cross-validation study to design bioelectrical impedance equations in Mexican adults. *Nutrition Journal*. 2007;6(1):1-7.

75. Manios Y, Kanellakis S, Androutsos O, et al. Development and validation of a simple model based on anthropometry: estimating fat mass for White postmenopausal women. *Menopause*. Apr 2012;19(4):467-70. doi:10.1097/gme.0b013e31823110db

76. Nickerson BS, Esco MR, Bishop PA, et al. Validity of BMI-Based Body Fat Equations in Men and Women: A 4-Compartment Model Comparison. *J Strength Cond Res*. Jan 2016;32(1):121-129. doi:10.1519/jsc.0000000000001774

77. O'Connor DP, Bray MS, McFarlin BK, Sailors MH, Ellis KJ, Jackson AS. Generalized Equations for Estimating DXA Percent Fat of Diverse Young Women and Men: The TIGER Study. *Medicine and Science in Sports and Exercise*. Oct 2010;42(10):1959-1965. doi:10.1249/MSS.0b013e3181dc2e71

78. Pascale LR, Grossman MI, Sloane HS, Frankel T. Correlations between thickness of skinfolds and body density in 88 soldiers. *Human Biology*. 1956;28(2):165.

79. Ramirez-Zea M, Torun B, Martorell R, Stein AD. Anthropometric predictors of body fat as measured by hydrostatic weighing in Guatemalan adults. *The American journal of clinical nutrition*. 2006;83(4):795-802.

80. Ramos-Jimenez A, Hernandez-Torres RP, Murguia-Romero M. Anthropometric equations for calculating body fat in young adults. *Archivos Latinoamericanos De Nutricion*. Jun 2018;68(2):111-121.

81. Rush EC, Plank LD, Laulu MS, Robinson SM. Prediction of percentage body fat from anthropometric measurements: comparison of New Zealand European and Polynesian young women. *The American journal of clinical nutrition*. 1997;66(1):2-7.

82. Segheto W, Coelho FA, Guimaraes da Silva DC, et al. Validity of body adiposity index in predicting body fat in Brazilians adults. *American Journal of Human Biology*. Jan-Feb 2017;29(1)e22901. doi:10.1002/ajhb.22901

83. Shafer KJ, Siders WA, Johnson LK, Lukaski HC. Body density estimates from upper-body skinfold thicknesses compared to air-displacement plethysmography. *Clin Nutr*. Apr 2010;29(2):249-54. doi:10.1016/j.clnu.2009.09.002

84. Skoufas E, Kanellakis S, Apostolidou E, et al. Development and validation of two anthropometric models estimating abdominal fat percentage in Greek adult women and men. *Clin Nutr ESPEN*. Dec 2018;28:239-242. doi:10.1016/j.clnesp.2018.07.010

85. Slaughter MH, Lohman TG, Boileau R, et al. Skinfold equations for estimation of body fatness in children and youth. *Human biology*. 1988:709-723.

86. Stout JR, Eckerson JM, Housh TJ, Johnson GO, Betts NM. Validity of percent body fat estimations in males. *Med Sci Sports Exerc*. May 1994;26(5):632-6.

87. Sun G, French CR, Martin GR, et al. Comparison of multifrequency bioelectrical impedance analysis with dual-energy X-ray absorptiometry for assessment of percentage body fat in a large, healthy population. *The American journal of clinical nutrition*. 2005;81(1):74-78.

88. Wattanapenpaiboon N, Lukito W, Strauss BJG, Hsu-Hage BH, Wahlqvist ML, Stroud DB. Agreement of skinfold measurement and bioelectrical impedance analysis (BIA) methods with dual energy X-ray absorptiometry (DEXA) in estimating total body fat in Anglo-Celtic Australians. *International Journal of Obesity*. Sep 1998;22(9):854-860. doi:10.1038/sj.ijo.0800672

89. Yao M, Roberts SB, Ma G, Pan H, McCrory MA. Field methods for body composition assessment are valid in healthy chinese adults. *J Nutr*. Feb 2002;132(2):310-7. doi:10.1093/jn/132.2.310

90. Zanovec M, Johnson L, Marx B, Keenan M, Tuuri G. Self-reported physical activity improves prediction of body fatness in young adults. *Medicine+ Science in Sports+ Exercise*. 2009;41(2):328.
