# Supplementary material for: Criterion-Related Validity of Field-Based Methods and Equations for Body Composition Estimation in Adults: A Systematic Review
Source: Curr Obes Rep. 2022 Nov 11;11(4):336–49. doi: 10.1007/s13679-022-00488-8 (PMC9729144; doi:10.1007/s13679-022-00488-8)
Supplement: Supplementary file 9 — Supplementary file9 (DOCX 26 KB) [file 13679_2022_488_MOESM9_ESM.docx]

**Supplementary Table S6.** Advantages and limitations of the “gold-standard” methods, and valid field-based methods and equations used for the assessment of body composition in adults.

| **GOLD-STANDARD METHODS^1-3^** | | |
| --- | --- | --- |
| **Advantages** | | **Limitations** |
| **Air displacement plethysmography** | High accuracy, relatively short time of measurement, non-invasive | High cost, large space, non-portable, specialized technicians, some contraindications (e.g. claustrophobia, anxiety), usually available only in research environments |
| **Deuterium oxide** | High accuracy, easy administration | High cost, specialized technicians, usually available only in research environments |
| **Dual-energy X-ray absorptiometry** | High accuracy, relatively short time of measurement,  assessment of regional body composition, bone mineral density | High cost, large space, long time of calibration, non-portable, specialized technicians, usually available only in research/clinical environments |
| **Magnetic resonance imaging** | High accuracy, non-invasive | High cost, large space, long time of measurement, non-portable, specialized technicians, some contraindications (e.g. metallic objects or pacemakers, claustrophobia), usually available only in research environments |
| **Underwater weighing** | High accuracy, non-invasive | High cost, large space, long time of measurement, non-portable, specialized technicians, some contraindications (e.g. claustrophobia, anxiety), usually available only in research environments |
| **FIELD-BASED METHODS**^4^***** | | |
| **Waist Circumference** | Low cost, easy of calculation, short time of measurement, non-invasive, no specialized technicians required, adaptable to different settings, implies central adiposity measurement | Superficial measurement, may be influenced by the timing of measurement (e.g., preprandial and postprandial period), require the individual to remove clothing to obtain a more accurate measurement, specific populations’ cutoffs |
| **Body Adiposity Index** | Low cost, easy of calculation, short time of measurement, non-invasive, no specialized technicians required, adaptable to different settings, implies central adiposity measurement | Superficial measurement, may be influenced by the timing of measurement (e.g., preprandial and postprandial period), require the individual to remove clothing to obtain a more accurate measurement |
| **Body Mass Index** | Low cost, easy of calculation, short time of measurement, non-invasive, no specialized technicians required, adaptable to different settings | Superficial measurement, may be influenced by the timing of measurement (e.g., preprandial and postprandial period), overall index of adiposity, misleading information about fat distribution in specific populations (e.g., athletes), specific populations’ cutoffs |
| **EQUATION/ INDEXES^2, 3^*** | | |
| **Durnin/Womersley equation** | Low cost, easy of calculation, relatively short time of measurement (depending on the number of SKF measured, four SKF may involve more time), SKF implies a subcutaneous adipose tissue measurement, non-invasive, can be extrapolated to the general population, adaptable to different settings | Medium-high cost depending on the type of caliper (plastic calipers are the most affordable), SKF requires tester experience, SKF may require the individual to remove clothing to obtain a more accurate measurement |
| **Jackson/Pollock equation** | Low cost, easy of calculation, relatively short time of measurement (depending on the number of SKF measured, seven SKF may involve more time), SKF implies a subcutaneous adipose tissue measurement, non-invasive, adaptable to different settings | Specific for the male population, medium-high cost depending on the type of caliper (plastic calipers are the most affordable), SKF requires tester experience, SKF may require the individual to remove clothing to obtain a more accurate measurement |
| **Jackson, Pollock and Ward equation** | Low cost, easy of calculation, relatively short time of measurement (depending on the number of SKF measured, seven SKF may involve more time), SKF implies a subcutaneous adipose tissue measurement, non-invasive, adaptable to different settings | Specific for the female population, medium-high cost depending on the type of caliper (plastic calipers are the most affordable), SKF requires tester experience, SKF may require the individual to remove clothing to obtain a more accurate measurement |
| **Equations combining abdominal circumference + skinfolds (SKF)** | Low cost, easy of calculation, SKF implies a subcutaneous adipose tissue measurement, non-invasive, can be extrapolated to the general population, adaptable to different settings | Medium-high cost depending on the type of caliper (plastic calipers are the most affordable), SKF requires tester experience, relatively long time of measurement (depending on the number of SKF measured), require the individual to remove clothing to obtain a more accurate measurement |
| **Equations based on waist circumference** | Low cost, easy of calculation, short time of measurement, non-invasive, can be extrapolated to the general population, no specialized technicians required, adaptable to different settings | Superficial measurements, require the individual to remove clothing to obtain a more accurate measurement |
| **Equations combining waist circumference + SKF** | Low cost, easy of calculation, SKF implies a subcutaneous adipose tissue measurement, non-invasive, adaptable to different settings | Medium-high cost depending on the type of caliper (plastic calipers are the most affordable), SKF requires tester experience, relatively long time of measurement (depending on the number of SKF measured), require the individual to remove clothing to obtain a more accurate measurement |
| **Equations combining waist circumference + SKF + Body Mass Index** | Easy of calculation, SKF implies a subcutaneous adipose tissue measurement, non-invasive, can be extrapolated to the general population, adaptable to different settings | Medium-high cost depending on the type of caliper (plastic calipers are the most affordable), SKF requires tester experience, longer time of measurement, SKF may require the individual to remove clothing to obtain a more accurate measurement, BMI implies specific populations’ cutoffs |
| **Equations combining various circumferences** | Low cost, easy of calculation, non-invasive, no specialized technicians required, adaptable to different settings | Superficial measurements, longer time of measurement (depending on the number of circumferences measured), may require the individual to remove clothing to obtain a more accurate measurement |
| **Equations combining circumferences + SKF** | Low cost, easy of calculation, SKF implies a subcutaneous adipose tissue measurement, non-invasive, can be extrapolated to the general population, adaptable to different settings | Medium-high cost depending on the type of caliper (plastic calipers are the most affordable), SKF requires tester experience, longer time of measurement , may require the individual to remove clothing to obtain a more accurate measurement |
| **Equations implying sum of SKF** | Low cost, easy of calculation, SKF implies a subcutaneous adipose tissue measurement, non-invasive, can be extrapolated to the general population, adaptable to different settings | Medium-high cost depending on the type of caliper (plastic calipers are the most affordable), SKF requires tester experience, relatively long time of measurement (depending on the number of SKF measured), SKF may require the individual to remove clothing to obtain a more accurate measurement |
| **SKF + Body Mass Index** | Low cost, easy of calculation, SKF implies a subcutaneous adipose tissue measurement, non-invasive, can be extrapolated to the general population, adaptable to different settings | Medium-high cost depending on the type of caliper (plastic calipers are the most affordable), SKF requires tester experience, longer time of measurement, SKF may require the individual to remove clothing to obtain a more accurate measurement, BMI implies specific populations’ cutoffs |
| **Body Adiposity Index equations** | Low cost, easy of calculation, short time of measurement, non-invasive, no specialized technicians required, adaptable to different settings | Different population equations, may require the individual to remove clothing to obtain a more accurate measurement |
| **Bioelectrical Impedance Analysis equations** | Easy of calculation, short time of measurement, non-invasive, can be extrapolated to the general population, adaptable to different settings | Different population equations, overestimation in body fat in individuals with severe obesity |
| **Body Mass Index** | Low cost, easy of calculation, short time of measurement, non-invasive, can be extrapolated to the general population, no specialized technicians required, adaptable to different settings | Specific populations’ cutoffs |
| **Waist to Height Ratio equations** | Low cost, easy of calculation, short time of measurement, non-invasive, can be extrapolated to the general population, no specialized technicians required, adaptable to different settings | Superficial measurements, may require the individual to remove clothing to obtain a more accurate measurement |

*Both the field-based methods and the equations/indexes summarized in this table have presented validity for estimating body composition in adults.

**REFERENCES**

1. Ceniccola GD, Castro MG, Piovacari SMF, et al. Current technologies in body composition assessment: Advantages and disadvantages. *Nutrition* 2019;**62**:25-31.

2. Fosbøl MØ, Zerahn B. Contemporary methods of body composition measurement. *Clinical physiology and functional imaging* 2015;**35**:81-97.

3. Gatterer H, Schenk K, Burtscher M. Assessment of human body composition methods and limitations. *Body composition*. CRC Press; 2017, p. 13-26.

4. Ross R, Neeland IJ, Yamashita S, et al. Waist circumference as a vital sign in clinical practice: A consensus statement from the ias and iccr working group on visceral obesity. *Nature Reviews Endocrinology* 2020;**16**:177-89.
