# Supplementary material for: Criterion-Related Validity of Field-Based Methods and Equations for Body Composition Estimation in Adults: A Systematic Review
Source: Curr Obes Rep. 2022 Nov 11;11(4):336–49. doi: 10.1007/s13679-022-00488-8 (PMC9729144; doi:10.1007/s13679-022-00488-8)
Supplement: Supplementary file 10 — Supplementary file10 (DOCX 13 KB) [file 13679_2022_488_MOESM10_ESM.docx]

**Supplementary Table S7.** Durnin and Womersley (1974) generalized equations and, Siri and Brozek converted formulas for body fat calculation in adults.

| **Durnin and Womersley 1974** | | |
| --- | --- | --- |
| **Age (years)** | **Females** | **Males** |
| < 17 | BD= 1.1369 – (0.0598 X Log) | BD= 1.1533 – (0.0643 X Log) |
| 17-19 | BD= 1.1549 – (0.0678 X Log) | BD= 1.1620 – (0.0630 X Log) |
| 20-29 | BD= 1.1599 – (0.0717 X Log) | BD= 1.1631 – (0.0632 X Log) |
| 30-39 | BD= 1.1423 – (0.0632 X Log) | BD= 1.1422 – (0.0544 X Log) |
| 40 -49 | BD= 1.1333 – (0.0612 X Log) | BD= 1.1620 – (0.0700 X Log) |
| > 50 | BD= 1.1339 – (0.0645 X Log) | BD= 1.1715 – (0.0779 X Log) |
| BD, Body Density; Log, Log of the Sum of 4 skinfolds.  Skinfolds: Bicep, Subscapular, Suprailiac, Tricep.  *Siri formula: Body Fat (%) = (4.95 / Body Density) – 4.50  **Brozek formula: Body Fat (%) = (4.57 / Body Density) – 4.142  *no significant differences arose from the use of both body density formulas* | | |
