# Supplementary material for: Criterion-Related Validity of Field-Based Methods and Equations for Body Composition Estimation in Adults: A Systematic Review
Source: Curr Obes Rep. 2022 Nov 11;11(4):336–49. doi: 10.1007/s13679-022-00488-8 (PMC9729144; doi:10.1007/s13679-022-00488-8)
Supplement: Supplementary file 11 — Supplementary file11 (DOCX 15 KB) [file 13679_2022_488_MOESM11_ESM.docx]

**Supplementary Table S8.** Jackson, Pollock and Ward (1980), and Jackson and Pollock (1978) generalized equations and, Siri and Brozek converted formulas for body fat calculation in adults.

| **Age (years)** | **Jackson, Pollock and Ward 1980 (females)** |
| --- | --- |
| 18-55 | BD= 1.0994921 – (0.0009929 x sum of 3 skinfolds) + (0.0000023 x square of the sum of 3 skinfolds) – (0.0001392 x age) |
| 18-55 | BD= (0.29669 x sum of 4 skinfolds) – (0.00043 x square of the sum of 4 skinfolds) + (0.02963 x age) + 1.4072 |
| 18-55 | BD= 1.097 – (0.00046971 x sum of 7 skinfolds) + (0.00000056 x square of the sum of 7 skinfolds) – (0.00012828 x age) |
| **Age (years)** | **Jackson and Pollock 1978 (males)** |
| 18-61 | BD= 1.10938 – (0.0008267 x sum of skinfolds) + (0.0000016 x square of the sum of 3 skinfolds) – (0.0002574 x age) |
| 18-61 | BD= (0.29288 x sum of 4 skinfolds) – (0.0005 x square of the sum of 4 skinfolds) + (0.15845 x age) – 5.76377 |
| 18-61 | BD= 1.112 – (0.00043499 x sum of 7 skinfolds) + (0.00000055 x square of the sum of 7 skinfolds) – (0.00028826 x age) |
| BD, Body Density.  Skinfolds females: Suprailiac, Thigh, Tricep (Sum 3); Abdominal, Suprailiac, Thigh, Tricep (Sum 4); Abdominal, Axila, Chest, Subscapular, Suprailiac, Thigh, Tricep (Sum 7).  Skinfolds males: Abdominal, Pectoral, Thigh (Sum 3); Abdominal, Suprailiac, Thigh, Tricep (Sum 4); Abdominal, Axila, Chest, Subscapular, Suprailiac, Thigh, Tricep (Sum 7).  *Siri formula: Body Fat (%) = (4.95 / Body Density) – 4.50  **Brozek formula: Body Fat (%) = (4.57 / Body Density) – 4.142  *no significant differences arose from the use of both body density formulas* | |
