# Supplementary material for: Criterion-Related Validity of Field-Based Methods and Equations for Body Composition Estimation in Adults: A Systematic Review
Source: Curr Obes Rep. 2022 Nov 11;11(4):336–49. doi: 10.1007/s13679-022-00488-8 (PMC9729144; doi:10.1007/s13679-022-00488-8)
Supplement: Supplementary file 12 — Supplementary file12 (PPTX 512 KB) [file 13679_2022_488_MOESM12_ESM.pptx]

## Slide 1
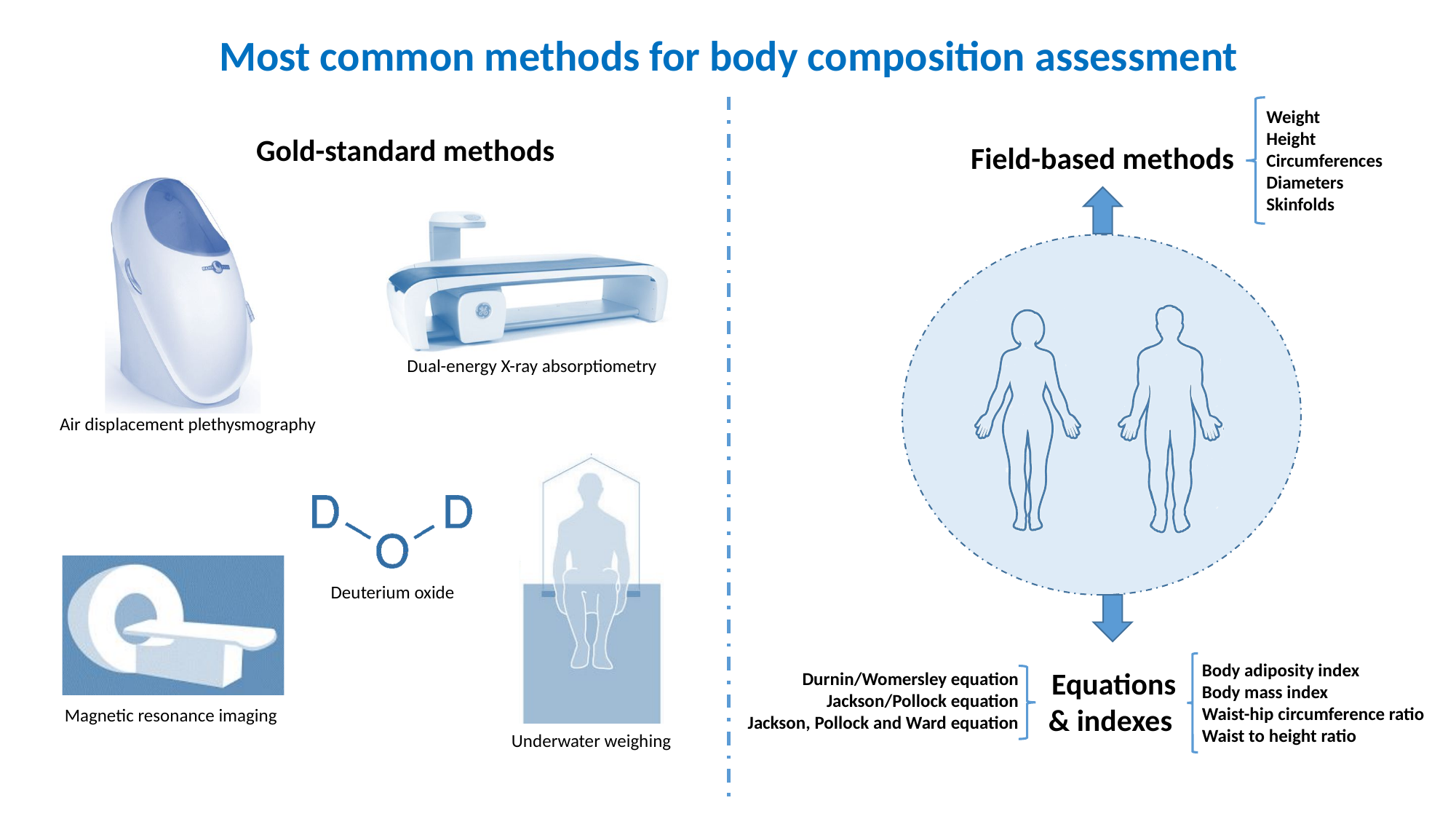

Most common methods for body composition assessment
Weight
Height
Circumferences
Diameters
Skinfolds
Gold-standard methods
Field-based methods
Dual-energy X-ray absorptiometry
Air displacement plethysmography
Deuterium oxide
Body adiposity index
Body mass index
Waist-hip circumference ratio
Waist to height ratio
Equations & indexes
Durnin/Womersley equation
Jackson/Pollock equation
Jackson, Pollock and Ward equation
Magnetic resonance imaging
Underwater weighing
